# Using Differential Scanning Calorimetry to Accelerate Polymerization Catalysis: A Toolkit for Miniaturized and Automated Kinetics Measurements

**DOI:** 10.1021/acscatal.5c01758

**Published:** 2025-04-11

**Authors:** Thomas M. McGuire, David Ning, Charlotte K. Williams

**Affiliations:** Department of Chemistry, University of Oxford, Oxford OX1 3TA, U.K.

**Keywords:** differential scanning calorimetry, polymerization, catalysis, lactones, cyclic carbonates, epoxides, anhydrides

## Abstract

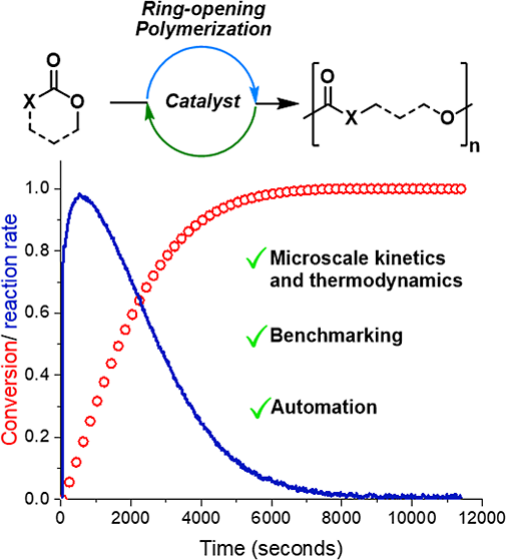

Understanding catalysts, and improving their future performances, requires quantification of their kinetic and thermodynamic parameters, including measurement of rate constants (*k*_obs_), transition state enthalpy barriers (Δ*H*^‡^) and polymerization enthalpy and entropy (Δ*H*_p_, Δ*S*_p_). This work presents miniaturized and automated methods, conducted using common differential scanning calorimetry (DSC) instruments using <10 mg sample (polymer, solvent, initiator and catalyst), to reliably, accurately, and rapidly measure all these key catalyst performance parameters. The methods are tested using known and highly successful catalyst/alcohol systems (tin(II)bis(2-ethyl hexanoate), Sn(Oct)_2_, and benzyl alcohol, BnOH) for cyclic ester or carbonate ring-opening polymerizations, and a catalyst/ionic cocatalyst ((salcy)CrCl and Bu_4_NCl) system for epoxide/cyclic anhydride ring-opening copolymerizations—two growth fields in polymerization catalysis. The DSC-measured kinetic parameters are identical and less error prone than those determined using conventional lab-scale experiments by aliquot removal. The DSC kinetics are measured using significantly smaller amounts of materials, 600x less sample, while being significantly more time-efficient. The methods are successfully demonstrated in both neat monomer (bulk) and in solution phase reactions, both of which are common in catalyst testing and application to yield highly reproducible and accurate quantification of catalyst turn over frequency values, rate constants, activation parameters, and rate-determining transition-state enthalpies. In addition to quantifying kinetic parameters, a second methodology is exemplified for two cyclic carbonates, enabling measurement of polymerization enthalpy and entropy change. The paper outlines key recommendations that should enable researchers to apply the DSC method in polymerization catalysis.

## Introduction

All advances and large-scale applications of catalysts require accurate, reproducible and efficient methods to quantify reaction rates and kinetic and thermodynamic parameters.^1^ Homogeneous cyclic monomer ring-opening polymerization (ROP) catalysis is a key technology to produce oxygenated polymers, which are viewed as leading, sustainable alternatives to current commodity hydrocarbon plastics, elastomers, and materials.^2−5^ Of particular promise among oxygenated polymers are those derived from the ROP of cyclic esters and carbonates, and ring-opening copolymerization (ROCOP) of epoxides and anhydrides.^6−10^ Reactions to produce these materials rely on successful homogeneous catalysis in both commercial and research contexts: the best catalysts should polymerize different types of monomers to make new classes of oxygenated polymers with high activity, productivity, selectivity, and control.^1,11−13^ Polymerization control refers to the facility shown by some catalysts to mediate the polymer molar mass, dispersity, chemical composition, and sequence.^14−16^ In this field of catalysis, there are a wide range of successful homogeneous polymerization catalysts including innovative inorganic or organometallic complexes with coordinated initiators, inorganic complexes used with cocatalyst salts as two-component catalyst systems ,and organic molecules either used alone or as catalyst systems with alcohols (Lewis acids/bases/anions/cations).^16,17^

Conventionally, when evaluating a new polymerization catalyst, rates are quantified using monomer concentration (conversion) vs time data. Often, it is collected by removal and analysis of aliquots from either bulk (neat monomer) or solution reactions (with organic solvents). In the last two decades, the time cost and accuracy of these measurements has been improved by conducting polymerizations with in situ spectroscopy, most commonly in situ IR or NMR spectroscopy.^18−26^ All these kinetics experiments consume multigram quantities of monomer and catalyst, particularly as repeat measurements are essential to quantify error ranges. The consequence is that collecting the minimum data required for full catalyst kinetics (rate constants and law) and Eyring analyses (activation energy) is both material and time demanding.^27^ This can raise a particular challenge in new catalyst and monomer development and for systematic structure-performance relationship studies.^6^

Differential scanning calorimetry (DSC) is very widely used in polymer and materials science to characterize thermal transitions, e.g., polymer glass transitions and melting and crystallization temperatures. In a DSC experiment, the sample’s instantaneous heat flow is accurately measured against either temperature or time. In the context of considering using DSC to monitor polymerization catalysis, it is important to appreciate that such heat flow should directly correlate with the rate of reaction. Thus, it should be possible to create the well-known conversion vs time (or conversion vs temperature) by integrating reaction heat flow vs time profiles.^28^ By fitting kinetic models to these plots, rate constants, reaction orders (rate laws), pre-exponential factors, and transition state energies may be quantified. Over the years, DSC instruments and methods have been used for the quantitative analysis, often in viscous fluids/solids, of the kinetics of polymer resin formation, e.g., epoxy curing reactions.^29^

Calorimetry is the gold-standard for studying reaction kinetics; however, the use of DSC instruments, which are generally less precise in their heat flow measurements, has not been widely explored for monitoring homogeneous polymerization catalysts. This is quite surprising considering that many researchers in polymerization catalysis would be expected to have easy access to DSC instruments. Punydom and Xia have pioneered the use of DSC to analyze the polymerization of caprolactone and l-lactide using nonisothermal methods.^30−34^ Although these are very interesting results, the work was not benchmarked against conventional methods, preventing any insight into the sensitivity or generality of using DSC to measure polymerization rates. More generally, using DSC to monitor homogeneous polymerizations, especially those using 4–7-membered heterocycles, is attractive because they usually result in significant heat release as the monomer forms polymers. These common classes of heterocycle ROP and ring-opening copolymerizations (ROCOP) are thermodynamically driven by release of ring-strain (i.e., enthalpy change) but result in a translational entropy penalty.^11,24,35^ Hence, to balance these thermodynamic and kinetic parameters, new oxygenated monomers and catalysts are generally tested at moderate temperatures (80–120 °C) and in solution (0.5–2 M).^36−38^ Nonetheless, using organic solvents is undesirable environmentally and may negatively influence both polymerization kinetics and equilibrium.^39^ Further, industrial-scale polymerizations are rarely conducted using solvent but often conducted in neat, molten monomer;^40^ therefore, the development of both catalysts and kinetic methods operable over a wide temperature range in neat monomer is important. Using DSC to assess reaction kinetics could be useful since the experimental temperature is precisely controlled from −80 to 550 °C, which may enable small-scale measurements at temperatures that would be hard to maintain using standard lab equipment. In addition, when using a DSC, the entire reaction vessel (pan/crucible) is heated to the target temperature, preventing unwanted monomer evaporation or sublimation, which can complicate some lab-scale measurements.

**Figure 1:**
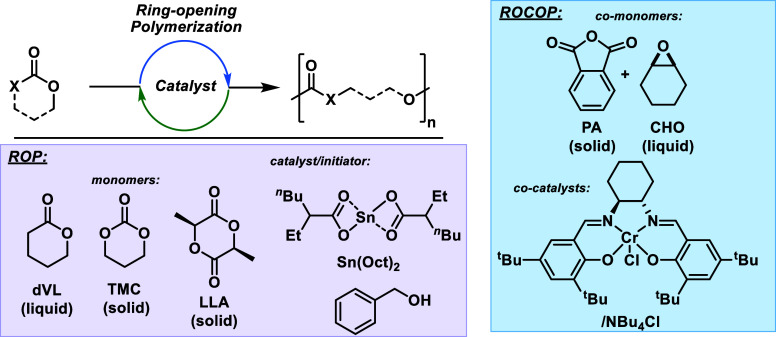
Monomers, polymerizations, and catalysts investigated in this work. For ROP, delta-Valerolactone (dVL), trimethylene carbonate (TMC), and l-lactide (L-LA) are each polymerized using the commercial tin(II) bis(2-ethylhexanoate) (Sn(Oct)_2_) and benzyl alcohol catalyst system. For cyclohexene oxide (CHO) and phthalic anhydride (PA) ROCOP, 1:1 molar quantities of (salcy)Cr(III)Cl complex: tetrabutyl ammonium chloride form the catalyst system.

We hypothesized that using the DSC instrument could be suitable for some types of kinetic and thermodynamic measurement, which are common to studying most catalysts and polymers. To test its ability to monitor reactions, it is important to establish its sensitivity, time resolution, temperature response, high-throughput capability, and overall material demand. The target cyclic monomer ROP and ROCOP reactions are highly selective equilibrium polymerizations; the objective is to test the potential for DSC methodologies to measure widely used catalytic kinetic parameters, like rate, activation enthalpy, and pre-exponential factor, and to measure reliably the monomer/polymer thermodynamic parameters, including the extent (conversion) of the reaction under specified conditions. The experiments are deliberately designed to use already known, successful, and widely used polymerization catalysts (Figure 1). These catalysts are selected to enable fair and generally understood comparisons against conventional measurements of polymerization kinetics. It is important to properly calibrate the measurement against alternative data collection methods, and this is best achieved using known catalysts. Naturally, if the methods are successful, they should be applicable, in future, to new catalyst investigation and development: understanding the optimum conditions for such measurements is also important to allow others to implement the methods more broadly.

## Methods

For detailed procedures regarding aliquot and in situ IR methods for monitoring polymerization; instrumentation; product quantification by size exclusion chromatography (SEC), ^1^H NMR and in situ IR spectroscopy; reaction profiles and kinetic treatments; Arrhenius plots and equilibrium monomer concentrations, please refer to the Supporting Information. Below, a representative method for the polymerization of delta-valerolactone, dVL, using the DSC instrument is outlined.

N.B. For polymerizations with dVL, stock solutions of catalyst and alcohol were prepared in pure monomer (dVL). No reaction was observed between the monomer and Sn(Oct)_2_ or between the monomer and benzyl alcohol at room temperature. The DSC crucibles used in all the kinetics and thermodynamics experiments were sealed (there was no piercing of the lid) to ensure constant mass and concentrations throughout the reaction.

In a glovebox, stock solutions of Sn(Oct)_2_ (1.0 M, 40.5 mg diluted to 0.1 mL in dVL) and benzyl alcohol (0.1 M, 10.8 mg diluted to 0.1 mL in dVL) were prepared. The two stock solutions (10 μL of each solution, 0.01 mmol, 1 equiv) were each added to a vial containing dVL (88 mg) to bring the total mass of dVL to 100 mg (1.0 mmol, 100 equiv) and the final loading of Sn(Oct)_2_: BnOH: dVL to 1:1:100. A DSC pan and hermetic lid were then tared, and the dVL reaction solution was transferred to the DSC pan via a micropipette (*ca* 3 μL). The pan and lid were sealed, reweighed, and the exact mass of the sample recorded. The pan was then transferred to a DSC instrument for polymerization. For isothermal measurements, the DSC cell was heated to the reaction temperature, before automated loading of the reference pan and sample pan. For dynamic measurements, the sample and reference pan were loaded at 40 °C. All measurements were carried out under a N_2_ flow (50 mL min^–1^). Once the reaction was complete, the pan was recovered and reweighed to confirm no mass loss had occurred during the reaction. Using a needle, the pan was carefully pierced and soaked in deuterated chloroform containing benzoic acid (catalyst quenching agent, *ca* 10 equiv) and mesitylene (NMR spectroscopic standard, 10.0 μL, 0.071 mmol). After recording the NMR spectrum, the sample was dried and diluted in THF (ca. 1 mL) for SEC analysis. For determination of activation parameters via isothermal methods, isotherms were held at temperatures between 80 and 150 °C until completion of the reaction.

## Results and Discussion

Given the lack of stirring and small masses used in DSC measurements, it was first essential to understand whether these changes to the usual polymerization scale and reaction setup would impact the reaction profile, observed rate constants (*k*_obs_), or polymer structure. In these first experiments, polymerizations were conducted with reaction rates compared using the DSC measurements vs equivalent reactions conducted in a vial, with regular aliquot removal, or in a Schlenk tube equipped with an in situ IR spectroscopy probe (REACTIR instrument). In the investigation, four monomers and two catalyst systems were selected to explore the influences of different chemistries, ring-strain, and monomer states (solid/liquid) on the kinetic measurements. For the cyclic ester or carbonate ROP, the catalyst system was tin(II)bis(2-ethyl hexanoate) (Sn(Oct)_2_) and benzyl alcohol (BnOH), which is selected as it is among the most widely used and successful in this field of polymerization catalysis.^13,41^ The tin/alcohol catalyst system shows field-leading rates, particularly at the higher temperatures used commercially to make these polymers, operates effectively at very low loadings, tolerates a wide temperature range, shows excellent polymerization control, and is used commercially to produce PLLA. The true catalyst is a tin-alkoxide species which forms in situ by equilibration between the tin carboxylate precursor and the alcohol additive.^42,43^ All the experiments used 1:1:100 molar loadings of [Sn(Oct)_2_]_0_/[BnOH]_0_/[monomer]_0_, with monomers including δ-valerolactone (dVL) used neat and l-lactide (LA) and trimethyl carbonate (TMC) both used in solution.^13,44^ These monomers and conditions were selected to probe different ring-strain values, different polymer chemistries, and different monomer states (crystalline solid vs liquid). For the cyclohexene oxide (CHO) and phthalic anhydride (PA) ring-opening copolymerization (ROCOP), the catalyst system was a Cr(III) complex ((salcy)CrCl) used with an ammonium chloride (Bu_4_NCl) cocatalyst. It was selected for its excellent rates, high temperature stability, and high selectivity for polyester formation—it is also quite widely used as the catalyst is commercial and straightforward to synthesize.^45,46^ It was tested at 1:1:50:2000 molar loadings of [Cr(salcy)Cl]_0_/[Bu_4_NCl]_0_/[PA]_0_/[CHO]_0_. In both polymerizations, the selected monomers and catalysts will allow testing of different polymerization enthalpies and temperatures (vide infra). Where catalysts were tested in solution, 1,2-dimethoxybenezene was selected as the solvent due to its wide operating temperature window (melting point ≈ 22 °C, boiling point ≈ 206 °C), which allows for both isothermal and dynamic temperature experiments and its moderate heat capacity (221 J mol^–1^). Since DSC experiments measure the change in heat flow during the reaction, both the fundamental polymerization enthalpy change and the material’s heat capacity affect the measurement sensitivity (which is different from spectroscopic analysis). 1,2-Dimethoxybenezene is also classified as a green solvent in some registers as it is bioderived and much less toxic than similar heat capacity and low volatility solvents such as 1,2-dichlorobenzene (*bp* = 148 °C) or 1,1,2,2-tetrachlorethane (*bp* = 148 °C), which are acute toxins and, in the case of 1,1,2,2-tetrachloroethane, fatal on inhalation or skin contact. Thus, it is a good choice for characterizing solution-phase catalysis—such experiments are very widely used in the academic community, although solvent selection is rarely rationalized. The DSC measurements were conducted using a general-level instrument, specifically using a TA Discovery DSC25 equipped with an autosampler, with temperature accuracy of ± 0.1 °C, temperature precision of ± 0.01 °C, enthalpy precision of ± 0.1% and baseline noise of <0.2 μW. It would be expected that other commercial, entry-level, DSC instruments with comparable parameters would function equivalently for these types of measurements.

To perform the DSC reactions, separate stock solutions of the metal complex catalyst, the cocatalyst or alcohol, and the monomer were prepared (see the Supporting Information for details). The components were mixed, under a nitrogen atmosphere, in the requisite molar ratio before being loaded via micropipette (*ca* 2–10 mg) into a DSC pan, which was then sealed. The sample mass was recorded before and after the reaction to ensure a quantitative mass balance. As benchmarks, equivalent anaerobic reactions were also set up for reaction conversion vs time monitoring in either Schlenk tubes equipped with magnetic stirrers and in situ IR spectroscopy probes or in a sealed vial equipped with magnetic stirrers, with aliquot removal and quenching at fixed time intervals, followed by monomer concentration determination using ^1^H NMR spectroscopy (*ca* 2 g of material per run, see the Supporting Information for details). Both DSC and lab-scale kinetic measurements were repeated in triplicate using freshly prepared batches of the stock solutions (monomer, catalyst, cocatalyst, or alcohol). For the DSC kinetics measurements, the data were analyzed using a common protocol for all polymerizations. These methods for data fitting and kinetic modeling are exemplified using Sn(II)-catalyzed δ-valerolactone ROP, and the outputs are discussed for other polymerizations (all data and kinetic fits are available in the Supporting Information).

**Figure 2:**
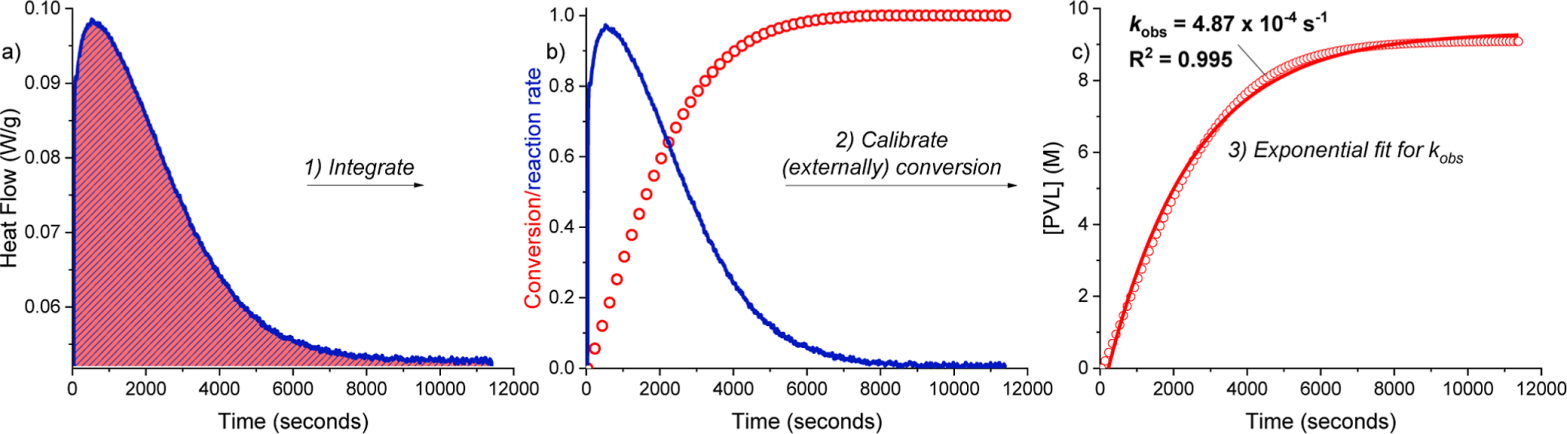
Key data collection and work-flow used to determine the polymerization pseudo-first-order rate constant, *k*_obs_, for δ-valerolactone (d-VL) ring opening polymerization (ROP) by a Sn(Oct)_2_/BnOH catalyst system. Polymerization Conditions: [Sn(Oct)_2_]_0_/[BnOH]_0_/[dVL]_0_ = 1:1:100, neat dVL, [dVL]_0_ = 9.98 M, 100 °C. (a) The DSC instrument produces data for plots of reaction heat flow vs time data (blue) line from 0 to 12,000 s; the red shaded area is the integral used to generate the conversion vs time data. (b) Plot showing both the normalized PVL conversion vs time (red circles) and the PVL polymerization rate vs time (blue line). (c) Plot showing the reaction [PVL] vs time, experimental data = red open circles and the exponential fit to the data (red line). The normalized dVL and PVL conversions were determined by analysis of the crude product (at 12,000 s) using ^1^H NMR spectroscopy where [PVL] = [dVL]_0_ – [dVL]_t_. The polymerization is highly selective, i.e., only product is PVL and quantitative mass balance is confirmed by weighing the sample before/after reaction. The DSC experiments were repeated, in every case, at least three times using freshly prepared batches of catalyst, monomer, and additives (cocatalyst/alcohol): in every case, there were very good fits to the experimental data and high reproducibility (as assessed by the error range for *k*_*obs*_).

The DSC method was tested using δ-valerolactone (dVL) ROP, with the Sn(Oct)_2_/BnOH catalyst system, at 100 °C and in the neat monomer ([dVL]_0_ = 9.98 M). The DSC instrument collects heat flow against time data, which shows a very sharp increase in intensity from 0 to 1000 s, followed by an apparent exponential decrease from 1000 to 12,000 s (Figure 2a). To convert the raw heatflow (rate) vs time data into the more common concentration (conversion) vs time data, it was integrated and normalized (for polymer conversion from 0 to 1). Each reaction was allowed to run for at least 5x half-life (*t*_1/2_ = 2000 s), and after such time, the DSC pan was reweighed, which confirmed no mass had been lost during the course of the reaction. The pan was then opened and the entire crude product analyzed using ^1^H NMR spectroscopy to determine the overall conversion and [PVL]_t_ (where [PVL] = 1-[dVL]_t_) (Figures 2b and S7). Using these data, the normalized conversion vs time data was converted to a [PVL] vs time profile (Figures 2c and S8). Both data sets are very well fit by exponential increases, and such a kinetic model is fully consistent with an expected first-order rate dependence on monomer concentration in the rate law; the gradient of the exponential fit to the [PVL] vs time data is the pseudo-first-order rate constant, *k*_obs_. The rate constant could also be determined using semilogarithmic methods (and linear fits). Experiments were conducted in triplicate, using fresh batches of stock solutions, and the rate constant (*k*_obs_) is 4.66 (±0.08) × 10^–4^ s^–1^ (Table 1).

**Table 1:** Polymerization Catalysis Data Obtained for dVL, LLA, and TMC ROP, and PA-CHO ROCOP Using Different Experimental Methods to Quantify the Polymerization Kinetics

#	mon	temp.(°C)	kinetic method	*k*_obs_ (×10^–4^ s^–1^)^a^	TOF (h^–1^)^b^	conv. (%)^c^	*M_n,_*_SEC_ (g mol^–1^) [D_M_]^d^	Overall sample mass used, including through repeat experiments (g)^[^e^]^
1^f^	dVL	100	vial	4.5 (±0.4)	93 (±9)	89 (±3)	12,700 [2.02]	6.00
2^f^	dVL	100	DSC	4.66 (±0.08)	101 (±3)	96 (±1)	11,000 [1.77]	0.015
3^g^	LLA	120	vial	18.0 (±2.5)	280 (±30)	81 (±3)	13,100 [1.39]^h^	6.00
4^g^	LLA	120	DSC	14.5 (±0.8)	260 (±10)	80 (±2)	13,000 [1.25]^h^	0.030
5^i^	TMC	120	vial	5.7 (±0.2)	93 (±10)	92 (±1)	12,600 [1.65]	6.00
6^i^	TMC	120	DSC	5.80 (±0.01)	105 (±1)	92 (±1)	13,000 [1.62]	0.030
7^j^	PA/CHO	100	vial	7.7 (±0.5)	77 (±9)	>99%	1700 [1.15]	6.00
8^j^	PA/CHO	100	DSC	7.9 (±0.4)	125 (±1)	>99%	1700 [1.15]	0.015

a The rate constants are all obtained by exponential fits to the polymer/monomer conversion vs time data.

b Catalytic Activity obtained as turnover frequency, TOF = # moles monomer consumed/# moles catalyst/time. For all experiments, the TOF was determined at 33% monomer conversion from the rate constant monitoring.

c The polymer conversion was determined at the end of the reaction using ^1^H NMR spectroscopy, with mass balance confirmed by weighing.

d Polymer molecular weight (*M*_n_) determined by SEC, with a THF eluent and polystyrene standards (PdVL, P(PA-*alt*-CHO), PTMC) or with CHCl_3_ eluent and polystyrene standards (PLLA).

e The total mass used for the combined triplicate measurements.

f Polymerization conditions: [Sn(Oct)_2_]_0_/[BnOH]_0_/[dVL]_0_ = 1:1:100 at 100 °C, [dVl]_0_ = bulk (9.98 M).

g Polymerization conditions [Sn(Oct)_2_]_0_/[BnOH]_0_/[LLA]_0_ = 1:1:100 at 120 °C, [LLA]_0_ = 1.0 M in 1,2-dimethoxybenzene.

h Correction factor of 0.58 applied.^47^

i Polymerization conditions [Sn(Oct)_2_]_0_/[BnOH]_0_/[TMC]_0_ = 1:1:100 at 120 °C, [TMC]_0_ = 2.0 M in 1,2-dimethoxybenzene.

j [CrSalen]_0_/[NBu_4_Cl]_0_/[PA]_0_/[CHO]_0_ = 1:1:50:2000 at 85 °C, [CHO]_0_ = 9.5 M in neat CHO.

Next, the Sn(II)-catalyzed polymerization was conducted under the same conditions but in a vial, with magnetic stirring, in a heating block, and under nitrogen. There was regular aliquot removal (under an inert environment) and analysis of the crude products by ^1^H NMR spectroscopy to determine dVL conversion and concentration. Once again, these experiments were repeated at least twice more, with fresh stock solutions, to enable the determination of errors. A third identical set of experiments, again with the same conditions and in triplicate, were conducted in a Schlenk tube, under nitrogen and heated in an oil bath, equipped with a magnetic stirrer and an in situ IR spectroscopy probe (REACTIR). In every case, these experiments yield concentration vs time plots, which are very clearly best fit by exponentials. Once again gradients are pseudo-first-order rate constants, *k*_obs_. The overlay of the data obtained from the DSC kinetic method and vial reaction aliquot analysis shows identical reaction profiles with the same rate constants, within error, i.e., *k*_obs_ = 4.66 (±0.08) × 10^–4^ s^–1^ (DSC method), 4.50 × 10^–4^ (±0.40) × 10^–4^ s^–1^ (vial aliquots) (Table 1, entries 1–2, Figures S9 and S10). These experiments clearly demonstrate that under the testing conditions, the unstirred small-scale DSC experiments show equivalent reaction kinetics to experiments analyzed by conventional methods. It is worth noting that the rates observed by in situ spectroscopy probes are slower and less reliable; this arises from difficulties in fully drying the probe and maintaining an anaerobic environment at the reaction temperature (Table S3). The data collected using the DSC methodology is very reproducible, showing the lowest of the error ranges at ± 0.08 × 10^–4^ s^–1^ for DSC vs ± 0.4 × 10^–4^ s^–1^ for vial reaction methods (Figures S8 and S10). The high reproducibility is significant since fresh batches of stock solutions can be expected to introduce weighing/balance errors. Overall, the experiments suggest that the DSC kinetic analysis method should match, and may even improve upon, that collected by using conventional experimental methods in this field of catalysis.

The crude samples were collected after the experiment for characterization to determine conversion (^1^H NMR spectroscopy) and the polymer’s number-average molecular weight/molar mass (*M*_n_) and dispersity (D̵) (SEC, Figures S9 and S11). Both the DSC kinetics and vial reaction methods produce PVL showing very similar molar mass and dispersity (DSC reaction: *M*_n,SEC_ = 11 kg mol^–1^, D̵ = 1.77, vial reaction *M*_n,SEC_ = 12.7 kg mol^–1^, *M*_n,theory_ = 9.7 kg/mol) and identical molar mass distribution (monomodal). The PVL formed in the Schlenk tube reaction showed significantly lower molar mass, consistent with issues in maintaining an anaerobic environment since protic impurities function as chain transfer agents and reduce the overall molar mass.

Following these successful proof of concept results, two other ROP using l-lactide (LLA) or trimethylene carbonate (TMC) were each investigated using the DSC kinetics method and the conventional vial and aliquot method. In all reactions, the same successful Sn(Oct)_2_/BnOH catalyst system was applied at 120 °C using [Sn(Oct)_2_]_0_/[BnOH]_0_/[monomer]_0_ of 1:1:100, where [LLA]_0_ = 1 M and [TMC]_0_ = 2 M in 1,2-dimethoxybenzene, respectively. Another series of experiments compared the DSC and vial aliquot methods for measurement of rates in the ring-opening copolymerization of phthalic anhydride (PA) and cyclohexene oxide (CHO). The PA/CHO ROCOPs were applied at 100 °C [Cr(salcy)Cl]_0_/[NBu_4_Cl]_0_/[PA]_0_/[CHO]_0_ = 1:1:50:2000, in neat CHO ([CHO]_0_ = 9.53 M). In all the different catalytic polymerizations, there was excellent agreement between the vial and DSC experimental data sets and the resulting polymerization rate constants obtained from kinetic fits to the data (Table 1, nos. 3–8, Figures S12–S26). Further, the polymers formed after these reactions all showed the same compositions and molecular weights, by characterization using ^1^H NMR spectroscopy and SEC (Figures S27–S29). As an added benefit, in many cases, the accuracy and reproducibility of the quantified rate constants were improved using the DSC kinetic methods as shown through the smaller error range as compared with vial or Schlenk reactions. This is perhaps due to the improved temperature control of the DSC instrument (Table 1, # 3–8). Finally, to exemplify that the DSC method can be used to monitor polymerizations with small amounts of catalyst, a further reaction of dVL was carried out at 140 °C using loadings 1:1:1000 [Sn(Oct)_2_]_0_/[BnOH]_0_/[dVL]_0_. A clear exotherm was detected, corresponding to a *k*_obs_ of 6.21 × 10^–4^ s^–1^, to form high molar mass PVL (*M*_n,SEC_ = 128,400 g mol^–1^) with a degree of polymerization (DP) of *ca* 900 as determined by ^1^H NMR spectroscopy (*M*_n,NMR_ = 89,9000 g mol^–1^Figures S30–S33). These combined findings, using different catalysts, monomers, and polymerization conditions all suggest that the DSC kinetics methodology should be broadly useful to study polymerization catalysis.

There are some notable benefits to using the DSC kinetic analysis compared with conventional methods: the most important being that the same data set is generated with approximately 600× less material. Further, the experiments are data-rich and may be more easily optimized than those where aliquots must be removed manually. All of these features should save both material and time, including limiting the need for repeated catalyst or monomer syntheses and purifications. Provided the DSC is equipped with an autosampler, which is a common accessory, reactions may be queued, allowing fast and automated collection of the data sets.

## Measurement of Catalytic Activation Energies Using DSC Methods

Understanding and measuring the catalytic transition states activation enthalpy change (*E*_a_, Δ*H*^‡^) and pre-exponential factor (A) is important to quantitatively compare catalysts, understand changes to selectivity (where there are byproducts), and substantiate mechanistic hypotheses, particularly when combined with DFT calculations. In conventional homogeneous polymerization catalysis experiments, such activation parameters are usually determined by performing >5 isothermal kinetics experiments, across a range of temperatures. It is possible that the DSC methods could be used to quantify these catalyst activation energies. In other fields of science, DSC instruments are used to obtain reaction activation parameters; these methods apply dynamic heating using either a single (e.g., Borchardt-Daniels methods)^48^ or multiple (e.g., Flynn-Wall-Ozawa methods) heating ramps.^49,50^ Relevant to polymerizations, these types of DSC measurements are commonly used estimate activation parameters in epoxy resin curing,^29^ but there are limited data on how estimates of activation parameters using dynamic methods compare with those generated using isothermal (Arrenhius) treatments. One drawback of using dynamic temperature methods is the introduction of an additional variable (changing temperature, dT), which complicates the numerical analysis and may impact the reliability of the measurement.

The potential for DSC dynamic methods to quantify the activation parameters was assessed through the ROP of dVL, LLA, and TMC. In every case, the activation enthalpy, Δ*H*^‡^, and ln *A* (pre-exponential factor) values were determined using isothermal experiments or using two common dynamic-temperature DSC methods: the Borchardt-Daniels and Ozawa methods (full discussion of these methods are included in the Supporting Information). First, each Sn(II)-catalyzed ROP was conducted isothermally at five different reaction temperatures from 100 to 140 °C, and each measurement/temperature was conducted in triplicate for error analysis. The temperature range was selected for its efficient monomer/polymer conversion and to limit any reagent or solvent evaporation. In all cases, the polymerization rates and rate constants increased with increasing temperature—this is fully consistent with the expected behavior (Tables S4–S6 and Figures S34–S42). For each Sn(II)-catalyzed ROP, the natural logarithm of the rate constant (ln *k*_obs_) was plotted against reciprocal temperature (1/*T*) and in every case, there were good linear fits to the data. These linear fits enable quantification of the transition state enthalpy (Δ*H*^‡^), as the gradient, and the pre-exponential factor (ln *A*), as the *y*-axis intercept, respectively (Figures S36, S39 and S42). These isothermal DSC experiments enabled determination of the catalytic transition state enthalpy barriers: Δ*H*^‡^ = 57.4 (±5.4) kJ mol^–1^ for dVL ROP, 59.7 (±5.4) kJ mol^–1^ for LLA ROP and 76.4 (±4.3) for TMC ROP, respectively. The transition state barriers are all quite similar, as might be expected due to the Sn(II)-catalyzed reactions following a common mechanism. Further, the error ranges are small and the overall barriers compare well with literature data for related catalysts and polymerizations of dVL, LLA, and TMC.^51,52^

**Figure 3:**
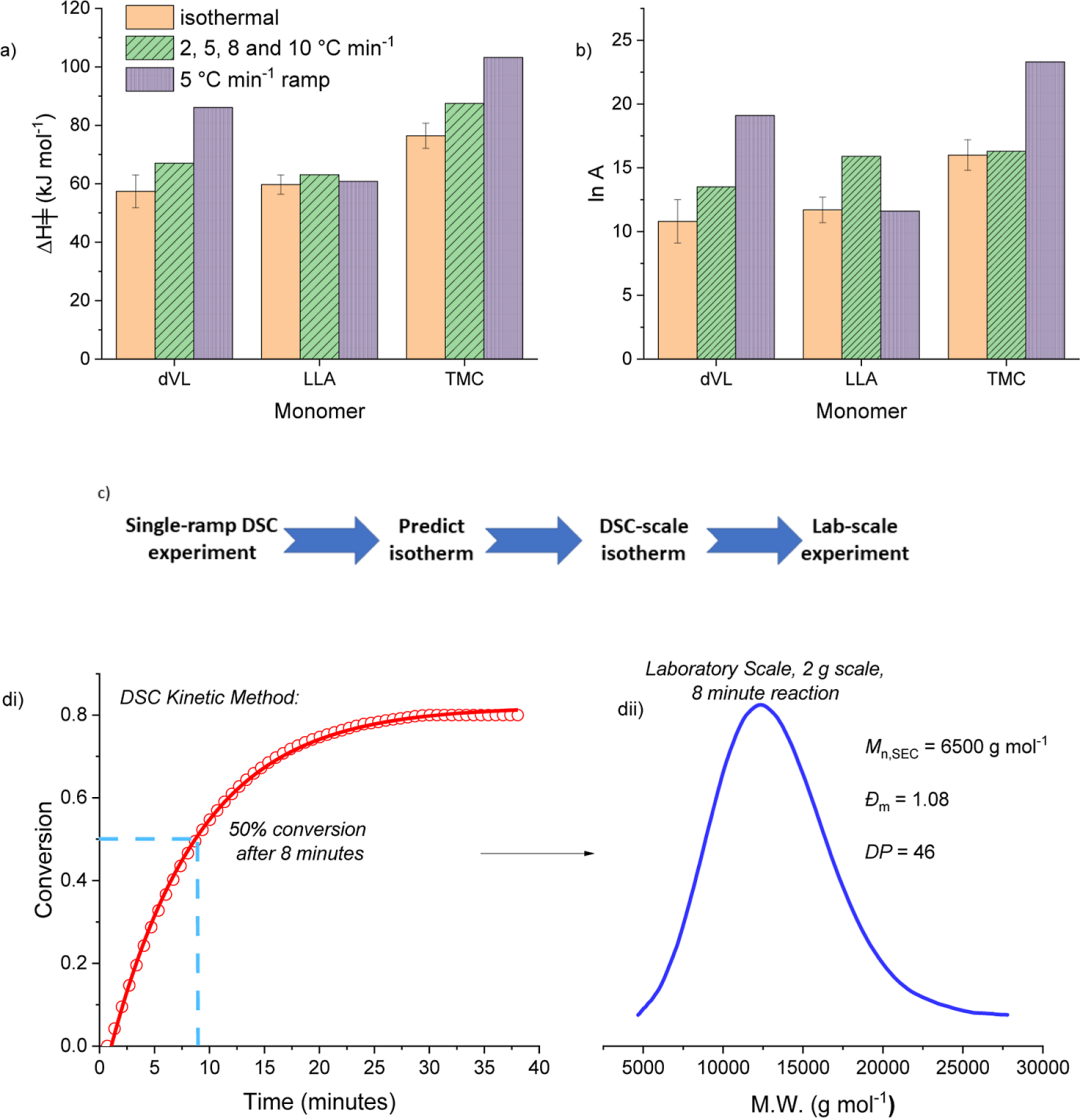
Using DSC methods both to measure and estimate transition state activation parameters for Sn(II)-catalyzed cyclic ester ROP (δ-valerolactone, l-lactide, trimethylene carbonate). The estimated kinetic data is used to target L-LA ROP conditions yielding specific PLLA compositions in laboratory scale experiments. (a,b) Histograms compare the transition state enthalpy, Δ*H*^‡^, values (a) and the pre-exponential factors, ln *A*, (b) determined using isothermal (orange, plain), Flynn-Wall-Ozawa (green, diagonal stripes) and Borchardt-Daniels (purple, dashes) methods for the three different monomers used in ROP. Isothermal reactions were performed in triplicate at 5 temperatures (100–140 °C). The Δ*H*^‡^ values were determined by plots of ln *k*_obs_ vs 1/*T*. Dynamic heating rates are over the range 2–10 °C/min, the Borchardt-Daniels methods are applied to data collected at 5 °C/min and the Flynn-Wall-Ozawa methods to 2, 5, 8, and 10 °C/min rates. Reaction Conditions: 1:1:100, [Sn(Oct)_2_]_0_/[BnOH]_0_/[monomer]_0_ =, [dVL]_0_ = 9.98 M, [LLA]_0_ = 1 M, [TMC]_0_ = 2 M. (c) Proposed workflow for use of dynamic heating in experimental reaction optimization. (d) L-LA ROP analyzed using the dynamic heating DSC methods to predict rates and conversions under specific conditions. (di) Plot showing PLLA conversion vs time, determined by DSC methods. The plot identifies that at ∼8 min, there should be 50% PLLA conversion and DP = 50. (dii) The laboratory scale experiment, after 8 min, produces gram-quantities of PLLA, the SEC chromatogram shows the PLLA DP = 46 and D̵ = 1.10. LLA ROP conditions for (d):1:1:100, [Sn(Oct)_2_]_0_/[BnOH]_0_/[LLA]_0_, [LLA]_0_ = 1.0 M in 1,2-dimethoxybenzene, 120 °C.

To understand the potential to apply DSC dynamic temperature methods, all three of the Sn(II)-catalyzed cyclic monomer ROP were performed using different heating rates from 2 to 10 °C min^–1^ and from 40 to 200 °C (Figures S43–S45). Using the dynamic temperature methods does not affect either the polymer composition or selectivity (^1^H NMR spectroscopy) or molecular weight (SEC analysis) (Figures S46 and S47). A series of experiments applied a single heating rate and the Borchardt-Daniels numerical methods to determine/estimate values for Δ*H*^‡^ and ln *A* (ASTME2041–23, Figures S2, S3 and S48–S58, Tables S1 and S7–S17).^48^ In brief, the method estimates the reaction rate constant, at a given temperature, from analysis of the heat flow vs temperature profile (more details in the Supporting Information). The transition state enthalpy and pre-exponential factors are subsequently determined using the same methods as already described. Using slow or intermediate heating rates generally resulted in closer agreement with the Δ*H*^‡^ values determined by isothermal experiments (Tables S20–S22). For example, for Sn(II)-catalyzed LLA ROP, the Δ*H*^‡^ = 59.7 ± 3.3 kJ mol^–1^ by isothermal measurements whereas dynamic heating DSC experiments at 5 °C min^–1^ heating rates gave Δ*H*^‡^ = 60.8 kJ mol^–1^, while heating at 10 °C min^–1^ gave Δ*H*^‡^ = 53.7 kJ mol^–1^. Similarly, better agreement with ln *A* values were obtained with slow or intermediate heating rates. E.g., for the same Sn(II)-catalyzed LLA ROP, the isothermal experiments resulted in ln *A* = 11.7 ± 1, while heating at 5 °C or 10 °C min^–1^ resulted in values of 11.6 and 9.3, respectively. The optimum heating rate for any dynamic heating rate (temperature) experiments is expected to be strongly reaction dependent, which complicates its use. Across these types of dynamic temperature change experiments, the resulting values for Δ*H*^‡^ and ln *A* often fell beyond the error ranges for the values determined by the more accurate isothermal methods (Figure 3a,b). Thus, isothermal DSC heating methods should be used to determine catalyst activation enthalpies (and pre-exponential factors) and these types of dynamic heating experiments can only provide approximations.

Next, DSC experiments were conducted with variable heating rates, using Flynn-Wall-Ozawa numerical analysis to construct log(heating rate) vs 1/T plots. These were used to estimate Δ*H*^‡^, which was further refined as outlined in ASTM E698-05 (see the Supporting Information for details, and Figures S6, S59, and S60, Tables S2, S18, and S19).^49,50^ Compared to the Borchardt-Daniels method, using the Flynn-Wall-Ozawa approach provides values which are in closer agreement with those determined by isothermal methods (Figure 3a,b, Tables S20–S22). For example, for the Sn(II)-catalyzed LLA ROP, the Δ*H*^‡^ and ln *A* values are the same, within error, as those measured by isothermal methods. For Sn(II)-catalyzed TMC ROP the values are also the same, within error, as those measured by isothermal kinetic methods. However, the Sn(II)-catalyzed dVL ROP was not so successful when analyzed using this method. Overall, these comparative catalytic polymerization data sets suggest that the Flynn-Wall-Ozawa method should be used to estimate the transition state activation parameters rather than the Borchardt-Daniels method. Nevertheless, the most accurate measurements result when conducting the DSC experiments isothermally over a range of different temperatures.

An interesting consideration for experimental scientists is the facility to use estimated transition state parameters to help optimize lab-scale reaction conditions. It may be possible to use faster dynamic heating DSC experiments to identify the right experimental conditions to use in larger (laboratory) scale polymerizations. To test this notion, the various estimated/measured Δ*H*^‡^ and pre-exponential factors were substituted into the Arrhenius equation, to predict the expected polymerization rate constant, *k*_obs_, under a specific set of conditions and temperatures. These rate constants were then used to estimate conversion versus time profiles for those conditions. These “back analyses” estimations conducted using the same catalysts and polymerizations showed that despite the differences in the estimations of Δ*H*^‡^ values between the most reliable isothermal and least reliable Borchardt-Daniels methods, the predicted conversion vs time plots are very similar over the selected temperature range (100–120 °C, Figure S61). The practical consequence for the experimental researcher is that dynamic kinetic measurements allow much faster data collection: a single 5 °C/min ramp from 40 – 200 °C takes 32 min, while performing the isothermal experiments at each temperature requires at least 10 h (*ca* 1–2 h for each temperature). It is, therefore, recommended that DSC dynamic heating rate experiments could be used to streamline reaction optimization (Figure 3c). For instance, by first using a DSC reaction with dynamic heating to predict the isothermal kinetic parameters, the observed rates can be predicted under a specific set of conditions and the outcome tested using a small-scale DSC experiment. The reaction can then be performed in the lab on a larger scale. These methods should improve the throughput of larger scale laboratory reactions and improve the accuracy of catalysis in producing target polymer properties. The importance of such an approach is appreciated by understanding all the reactions are well controlled polymerizations—i.e., using the conversion vs time profile enables targeting of specific polymer molar mass/DP. To exemplify the approach, a target PLLA was set showing a DP of ∼50 (*M*_*n*_ = 7 kg/mol) and a dispersity <1.10. The dynamic heating rate DSC kinetic methods were applied using 1:1:100 [Sn(Oct)_2_]_0_/[BnOH]_0_/[LLA]_0_, [LA]_0_ = 1.0 M in 1,2-dimethoxybenzene at 120 °C. These experiments allowed estimation of the required time for the polymerization to achieve PLLA DP = 50, i.e., 50% monomer conversion, to be ∼8 min at 120 °C (Figure 3di). The reaction was then scaled to 2 g (LLA), and conducted in a vial under otherwise identical conditions to the DSC experiments. The polymerization was quenched, by exposure to air and addition of solvents to precipitate the polymer, after 8 min. The resulting crude sample showed that PLLA with DP = 46 and dispersity of 1.08 (Figure 3dii, SEC, and ^1^H NMR). In our experience, using only a single larger-scale experiment it would be very difficult (and/or a random success) to so quickly achieve the target PLLA sample properties. Rather laboratory-scale synthesis of PLLA with a specific chain length and narrow dispersity usually involves a series of empirical optimization experiments, which can be rather material and catalyst wasteful. This proof-of-concept experiment exemplifies the practical utility of these the DSC kinetic methods to facilitate reaction optimization, prepare targeted polymer samples, to reduce waste chemicals and save time.

### DSC Experiments to Measure Polymerization Thermodynamic Parameters

Heterocycle ROP are often chemical equilibria between the monomer (heterocycle) and polymer. As mentioned, for 6-membered cyclic esters and carbonates, polymerization tends to be driven by enthalpy gain (release of ring-strain) but does involve an entropy cost.^11^ All practical uses of the polymerization catalysts require proper understanding and quantification of the polymerization thermodynamic parameters in order to select the most appropriate operating conditions. Further, the chemical equilibria can be run in reverse such that the polymers are chemically recycled to the heterocycles—such reactions are also catalyzed process and understanding the depolymerization thermodynamics is essential to select the most appropriate conditions.^53−55^ In conducting the kinetics experiments, using the DSC instrument, we wondered whether we could combine these small-scale experiments reaction profile analysis (i.e., establishing equilibrium was achieved), with the precise temperature control and ability to analyze the reaction products by spectroscopy. That data could be combined to determine the polymerization thermodynamic parameters. In testing the notion, it is notable that although there are quite a number of prior experimental measurements of the polymerization free energy, enthalpy and entropy change, these are not all reported under the same conditions, which makes comparisons between data sets very challenging.^11^ The most obvious issue is that many studies were conducted in dilute solutions, whereas most polymerizations and recycling processes are conducted in neat monomer/polymer. Thus, we decided to measure the equilibrium parameters for two cyclic carbonates for which there are not yet any prior reports in the literature and to make those measurements in the neat/bulk state.

**Figure 4:**
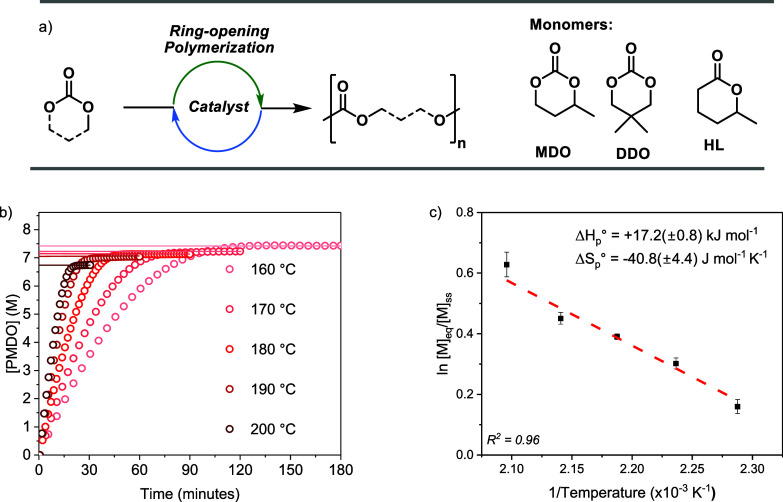
Using DSC methods to measure the cyclic carbonate ring-opening polymerization thermodynamic parameters. (a) The two cyclic carbonates used in these experiments: MDO and DDO. (b) Plot showing the [PMDO] conversion vs time data obtained from the ROP of MDO at temperatures of 160, 170, 180, 190, and 200 °C. Polymerization Conditions: 1:1:100, [Sn(Oct)]_2_/[BnOH]_0_/[MDO]_0_, in neat MDO, i.e. [MDO]_0_ = 8.61 M. [PMDO] = [MDO]_0_ – [MDO]_t_ The lines mark [PMDO]_eq_ for each reaction temperature. (c) Plot of natural logarithm of the equilibrium monomer concentration (ln[[M]_eq_]) vs reciprocal temperature. The standard state for each monomer, [M]_ss_ = 1 M. The polymerization thermodynamic parameters, Δ*H*_p_° and Δ*S*_p_°, are determined from the gradient and *y*-axis intercept of the linear fit to the data, respectively. The [M]_eq_ was determined by ^1^H NMR spectroscopy of the reaction product, at each temperature. The errors are the standard deviation of the mean determined from 3 repeat experiments.

The two 6-membered cyclic carbonates were 5,5-dimethyl-1,3-dioxan-2-one (DDO) and 4-methyl-1,3-dioxan-2-one (MDO) (Figure 4a). In neat monomer with [Sn(Oct)_2_]_0_/[BnOH]_0_/[cyclic carbonate]_0_ loadings of 1:1:100, the DSC methods were used to monitor polymerizations conducted isothermally, at temperatures from 130 to 200 °C. In each case, the heat-flow (rate) vs time data were used to establish that the polymerization equilibrium was achieved: this is clearly signaled by a plateau in the heat flow response or by the same plateau (corresponding to the equilibrium monomer concentration) in plots of monomer concentration vs time (Figure 4b). After the reaction, the equilibrium monomer concentration was determined by ^1^H NMR spectroscopy (Tables S23 and S24, Figures S63 and S65). Conducting these DSC experiments over a range of temperatures, and using Van’t Hoff analysis (plots of ln(K_eq_) vs 1/*T*), allowed for determination of the standard polymerization enthalpy (Δ*H*_p_°, gradient) and entropy (Δ*S*_p_°, *y* intercept) (Figures 4 and S66).

For each of the two cyclic carbonate ROP, both the Δ*H*_p_° and Δ*S*_p_° terms are negative. This is entirely consistent with such reactions generally being enthalpically, but not entropically, favorable (Table 2, entries 1 and 2). These thermodynamic values can be used to determine the polymer ceiling temperature (*T*_c_), i.e., the temperature at which the monomer/polymer free energy change is zero and above which polymerization is disfavored and depolymerization is favored (Table 2). The methyl-substituted cyclic carbonate (MDO) has a ceiling temperature of 477 °C, while the dimethyl-substituted monomer (DDO) has a lower value of 445 °C. These values for the ceiling temperatures are higher than for similar 6-membered ring cyclic esters (Table 2, entry 3). More detailed comparisons reveal that there is a slightly lower entropic penalty-associated polymerization of the analogous cyclic carbonate, MDO. For instance, comparison of the Δ*H*_p_° and Δ*S*_p_° terms for hexavalerolactone (HL) with MDO shows that Δ*H*_p_° terms are similar but the Δ*S*_p_° is significantly less negative (HL Δ*H*_p_° = −19.3 kJ mol^–1^, Δ*S*_p_° = −62.2 J mol^–1^ K^–1^; MDO Δ*H*_p_° = −17.2 kJ mol^–1^, Δ*S*_p_° = −40.7 J mol^–1^ K^–1^). The result is that the 6-membered ring cyclic carbonates have considerably higher ceiling temperatures. Interestingly, DDO has a higher ceiling temperature than MDO, despite it being a more substituted ring. This observation contrasts with the expected Thorpe Ingold effect where more ring substituents tend to increase the equilibrium monomer concentration in heterocycle ROP.^11^

**Table 2:** Polymerization Thermodynamic Parameters for the 6-Membered Ring Cyclic Carbonates^a^

entry	monomer	{M]0^b^	Δ*H*_p_° (k J mol^–1^)	Δ*S*_p_° (J mol^–1^ K^–1^)	*T*_c_ (°C)^c^
1	MDO	8.61	–17.2 (±0.8)	–40.7 (±4.4)	477
2	DDO	7.68	–25.0 (±0.1)	–51.7 (±0.7)	445
3	HL^24^	8.76	–19.3 (±0.5)	–62 (±2)	164

a Determined from the gradient (Δ*H*_p_°) and intercept (Δ*S*_p_°)of the ln [M]_eq_/[M]_ss_ vs 1/*T* plot for the ROP of the monomer. [M]_ss_ = 1.0 M.

b Bulk concentrations assume density of monomer is 1 g cm^–3^ and densities of monomer and polymer are the same.

c *T*_c_ = Δ*H*_p_°/Δ*S*_p_^BULK^, where Δ*S*_p_^BULK^ = Δ*S*_p_° + *R* ln([bulk monomer]_0_), where *R* is the ideal gas constant. [MMDO]_0_ = 8.61M, [DDO]_0_ = 7.68 M and [HL]_0_ = 8.76 M.

### Practical Guidelines for Future Investigations of Polymerization Catalysis Using DSC Methods

The results presented in this work suggest that using DSC methods may be beneficial for fast, accurate, and high-sensitivity monitoring of polymerization catalysis. As mentioned at the outset of this work, in the future, these methods should be explored for newly discovered polymerization catalysts where the benefits of miniaturization and automation could be significant. It is expected that these DSC measurements and methods can both expedite new catalyst discovery, improve understanding of mechanisms, and accelerate structure-performance relationship investigations. After having conducted this study, we have put together a set of recommendations and guidelines to help experimental researchers new to the use of DSC to monitor catalysis.

#### DSC Experiments Measure Rate

The consequence is that faster reactions will show a better sensitivity. As heterocycle ROP catalysis is almost always faster at higher temperatures (provided the catalyst is thermally stable), this means higher temperature experiments will result in greater signal responses (Figure S62). In making these measurements, we recommend absolute signal responses in the range 0.1–100 mW to ensure detectability (the typical baseline noise of the instrument we used is <0.2 μW) and to avoid thermal runaway. The catalytic reactions studied here all had signal responses in the range 0.3–2 mW, depending on the catalyst, polymerization, rate of reaction, mode (dynamic or isothermal), concentration and mass of sample. For dynamic DSC measurements, faster scan rates lead to greater signal response, as more monomer is present at higher temperatures (e.g., Figures S4 and S5).

#### Ensure Homogeneous Solutions at Room Temperature

Homogeneous catalysis relies on ensuring that all reagents and catalysts are dissolved. Any incomplete dissolution reagents will lead to inaccurate molar dosing. Further, any heat of dissolution or precipitation may interfere with the thermogram, resulting in inaccurate measurements of rate (Figure S67).

#### Reagent Concentration

The concentrations of reagents and catalysts will impact the instrument response. If the reagents are too dilute, an exotherm will be difficult to detect (Figure S68). On the other hand, very concentrated and highly exothermic reactions may lead to uncontrolled heating. It is noted that the latter case is very unlikely to apply to catalyzed heterocycle ROP or heterocycle/heteroallene ROCOP since the polymerization energy changes are quite low.

#### Watch out for Reagent/Product Thermal Transitions

The DSC instrument is sensitive to thermal transitions including reagent/product melting, boiling, or crystallization points. Care should be taken to avoid temperatures and conditions under which these might become relevant over the time span or temperature range of the measurement. They would be expected to interfere with, obscure, or complicate rate data (Figures S67 and S69). Furthermore, while it is possible to monitor polymerization using volatile reagents, provided the polymerization occurs at temperatures below the boiling point of the substrates, it may be challenging to accurately load highly volatile species (i.e., those with boiling points close to room temperature) as material rapidly evaporates on a small scale. This should be taken into consideration when designing reactions for use on the DSC.

#### Monitor the Instrument Baseline

When DSC methods are used to monitor polymerization catalysis, any changes to the shape and type of baseline may impact the integrated reaction profile, in turn affecting the measured rate constant. We recommend choosing the most appropriate baseline based on how the heat capacity of the material changes during the reaction (e.g., sigmoidal or horizontal tangent). When making comparisons between different catalysts/polymers, we have ensured that all data are processed using the same baseline type. One way to assess the impact of baseline correction is to repeat the kinetic analysis using different baseline types.^28^

#### Polymerization Enthalpy Change Range

In using DSC methods, care should be taken when measuring polymerizations resulting in low enthalpy changes. These would be expected to result from stable monomers (low ring-strain, often correlating with high degrees of substitution) or when using cyclic monomers which undergo entropically driven polymerizations (e.g., >15 membered ring lactones). In these cases, the low polymerization enthalpy will decrease the sensitivity of any kinetic measurements (Figure S68). Conversely, polymerizations resulting in high enthalpy change can lead to thermal runaway (i.e., uncontrolled heating of reactions), although as stated above, this is considered very unlikely when monitoring common heterocycle ROP catalysis.

#### Overall Sample Mass

We have conducted the DSC measurements using overall mass from 1 to 10 mg of sample. These were selected to ensure the polymerization heat flow is detectable and that homogeneous heating/constant temperature is maintained across the sample (i.e., to avoid thermal lag). As the integration of heat flow vs time to produce conversion vs time plots is self-normalizing, provided the conversion can be determined by other methods (e.g., NMR spectroscopy), the mass of the sample will not affect the shape of the reaction profile and obtained rate constants. However, the overall mass does influence the overall enthalpy change (mW/g), so it is important to record the input mass. Moreover, monitoring the sample mass is important to help the user determine if the vessel (pan) is properly sealed during the reaction, which is essential for polymerization equilibrium measurements.

#### Take Care with the Heat Capacity of Solvents

High heat capacity materials will decrease the sensitivity of the instrument, and so it is important to select the most appropriate solvent for any homogeneous catalysis investigations using DSC. For instance, chlorobenzene has a higher heat capacity than 1,2-dimethoxybenzene. This means that using chlorobenzene as the polymerization catalysis solvent rather than 1,2-dimethoxybenzene results in a lower absolute signal and reaction enthalpy, despite the fact similar rates are observed in both solvents (Figure S70).

#### Reactions Should Have Zero/Minimal Rates at Room Temperature

This requirement is important since the same must be loaded into the instrument, and if using an autosampler, the start of the reaction may occur sometime (hours) later. Thus, we recommend using catalysts that have zero or very low rates of polymerization at room temperature.

## Conclusions

A series of experimental methodologies to measure catalytic polymerization kinetics and thermodynamics using a DSC instrument, equipped with an autosampler, were described. These methods were used to measure the key catalytic parameters, including rate constants, activation enthalpies, and pre-exponential factors for two different catalysts applied across four different polymerizations. Cyclic monomer ring-opening polymerization and epoxide/anhydride ring-opening copolymerization catalysis were selected as they are high growth fields for catalyst development and fields in which experts from polymer and materials science use catalysis to make useful products. Here, miniaturized kinetic methods were demonstrated using very widely applied and commercial Sn(II) catalysts, used with alcohol initiators, for the ring-opening polymerization of d-valerolactone, l-lactide, and trimetheylene carbonate. Further, a robust and commercial Cr(III) catalyst, used with an ammonium chloride cocatalyst, was investigated in the ring-opening copolymerization of cyclohexene oxide and phthalic anhydride. Using the miniaturized and automated DSC methods, under a range of temperatures/heating rates and conditions, resulted in fast, accurate, and high-sensitivity data, which underpinned elucidation of the polymerization rates, orders in monomer (rate laws), activities, reaction profiles (extent of reaction/equilibrium monomer conversion), and transition state barriers. The small-scale DSC methods were demonstrated to be very helpful in identifying the optimum reaction conditions (temperature, time scale, and concentration) to produce polymers with targeted degrees of polymerization and dispersity. These methods of polymerization kinetic and thermodynamic analysis, using DSC instruments, are unfamiliar to many in the polymerization catalysis community, and so a series of recommendations and considerations for practical implementation of the methods are presented. In the future, it is clear that these methods should both accelerate new catalyst discovery, underpin structure–performance relationships and mechanistic insight, and improve operational sustainability through chemical and time reductions. We recommend using these methods to assess and compare new polymerization catalysts and to accelerate catalyzed polymerization uptake in the production of sustainable polymers and materials.

## Supporting Information Available

All the experimental details, including materials, instrumentation, and methods used in the polymerization kinetic measurements using the DSC instrument; product qualification by ^1^H NMR and IR spectroscopy and by SEC; and plots used for kinetic analyses, reaction profiles, Arrenhius plots, equilibrium monomer concentration measurements, and associated tabulated data. The following files are available free of charge. Supporting Information (PDF)
