# Supplementary material for: Using Differential Scanning Calorimetry to Accelerate Polymerization Catalysis: A Toolkit for Miniaturized and Automated Kinetics Measurements
Source: ACS Catal. 2025 Apr 11;15(9):6760–71. doi: 10.1021/acscatal.5c01758 (PMC12053833; doi:10.1021/acscatal.5c01758)
Supplement: Supplementary file 1 — cs5c01758_si_001.pdf [file cs5c01758_si_001.pdf]

Supporting information:

Using Differential Scanning Calorimetry to Accelerate Polymerization Catalysis: a Toolkit for Miniaturized and Automated Kinetics Measurements

Thomas M. McGuire, David Ning and Charlotte K. Williams*

[a] Department of Chemistry, Chemistry Research Laboratory, University of Oxford, 12 Mansfield Rd, Oxford, OX1 3TA, U.K

Corresponding Authors

Charlotte K. Williams: Email: charlotte.williams@chem.ox.ac.uk

Supporting information: 1
1. Materials 3
2. Methods 4
   2.1 Polymerization Method using a DSC pan 4
   2.2 Polymerization Method with Aliquot Analysis 4
   2.3 Polymerization Method using *in-situ* ATR-IR Spectroscopy 4
   2.4 Estimation of Activation Parameters using Dynamic Heating Rates 4
3. Additional information 11
   3.1 Benchmarking DSC kinetics 11
   3.2 Isothermal kinetics for dVL, LLA and TMC ROP and PA/CHO ROCOP 27
   3.3 ROP of dVL, LLA and TMC under dynamic heating 34
   3.5 dVL ROP kinetics by single heating ramp 37
   3.5 LLA ROP kinetics by single heating ramp 40
   3.6 TMC ROP kinetics by single heating ramp 44
   3.7 LLA and TMC kinetics by multi-heating ramps 48
   3.8 Comparison of methods for determination of activation paramaters for the kinetics of dVL, TMC and LLA ROP 49
   3.9 Determination of equilibrium monomer conversions 52
   3.10 Examples for new users 57
4. References 59

1. Materials

All experiments were carried out, under N_2, using standard Schlenk/glovebox techniques, unless otherwise stated. All chemicals were stored, under N_2, unless otherwise stated. 1,2-Dichlorobenzene was purchased from Sigma-Aldrich. Prior to use, 1,2-dichlorobenzene was degassed, by N_2 purge, and stored over 3 Å molecular sieves under N_2. δ-Valerolactone (dVL) was purchased from Tokyo Chemical Industries. Prior to use, the lactone was stirred over calcium hydride, overnight, and then fractionally distilled under reduced pressure. L-Lactide (LLA) was purchsed from Sigma-Aldrich. Prior to use, LLA was recrystallised from toluene, three times, then sublimed, under reduced pressure. Trimethylene carbonate (TMC) was purchased from Tokyo chemical industry. Prior to use, TMC was recrystallised from anhydrous diethylether, two times. Phthalic anhydride (PA) was purchased from Sigma-Aldrich. Prior to use, PA was purified by stirring in dry toluene, at room temperature, for 16 hours. The supernatant was filtered, and the toluene removed in vacuo. The resultant white powder was crystallised from hot (60 °C) chloroform then sublimed under vacuum at 80 °C. Cyclohexene oxide (CHO) was purchased from Acros Organics. Prior to use, the epoxide was stirred over calcium hydride overnight and fractionally distilled, under reduced pressure, twice. 4-methyl-1,3-dioxan-2-one (MDO) was synthesised according to literature.[1] Prior to use, MDO was dried over CaH_2 and fractionally distilled, under reduced pressure. 5,5-dimethyl-1,3-dioxan-2-one (DDO).[1] Prior to use, DDO was twice recrystallised from anhydrous diethyl ether. Tin(II) 2-ethylhexanoate ($Sn(Oct)_2$) was purchased from Sigma-Aldrich and used as received. Anhydrous benzyl alcohol was purchased from Sigma-Aldrich and used as received. (*R,R*)-(−)-*N,N'*-Bis(3,5-di-*tert*-butylsalicylidene)-1,2-cyclohexanediaminochromium(III) chloride ((salcy)CrCl) was purchased from strem chemicals and used as received. Tetrabutylammonium chloride was purchased from Sigma-Aldrich and used as received

Size Exclusion Chromatography (SEC) was carried out on a Shimadzu LC-20AD instrument using two PSS SDV linear M columns in series, with a THF eluent. Measurements were conducted at 30 °C, with a flow rate of 1 mL/min. Samples were detected with a differential refractive index (RI) detector. Number-average molar mass ($M_{n,SEC}$) and dispersities ($Đ_M$ (M_w/M_n)) were determined using narrow molar mass polystyrene calibrants (M_n = 500 – 1000000 g mol^{-1}) . The polymer samples were dissolved in HPLC-grade THF, at a concentration of *ca* 10 mg/mL, and filtered through a 0.2 μm microfilter prior to analysis.

Differential scanning calorimetry (DSC) was performed using a TA Discovery 25-Auto. See methodology section for detailed outline on DSC measurments. Dynamic kinetic analysis was performed using Trios software (v5.7.1.74) with the kinetics add-on package.

In-situ FTIR spectra were recorded using a Mettler-Toledo DiComp sentinel probe.

NMR spectra were obtained using a Bruker AVIII HD nanobay NMR spectrometer. Coupling constants are given in Hertz. Selectivities were determined by ^1H NMR spectroscopy.

Turnover Frequency (TOF) calculations were taken at 33% conversion for all reactions.

2. Methods

2.1 Polymerization Method using a DSC pan

N.B. For polymerizations with dVL, stock solutions were prepared in pure monomer. No reaction was observed between the monomer and $Sn(Oct)_2$ or monomer and benzyl alcohol.

In a glovebox, stock solutions of $Sn(Oct)_2$ (1.0 M, 40.5 mg diluted to 0.1 mL in dVL) and benzyl alcohol (0.1 M, 10.8 mg diluted to 0.1 mL in dVL) were prepared. 10 µL of both stock solutions (0.01 mmol, 1 equiv.) were added to a vial containing dVL (88 mg) to bring the total mass of dVL to 100 mg (1.0 mmol, 100 equiv.) and the final loading of $Sn(Oct)_2$: BnOH: dVL to 1: 1: 100.

A DSC pan and hermetic lid were then tared and the dVL reaction solution was transferred to the DSC pan via micropipette (*ca* 3 µL). The pan and lid were sealed, reweighed and the exact mass of the sample recorded. The pan was then transferred to a DSC instrument for polymerization. For isothermal measurments, the DSC cell was heated to the reaction temperature, before automated loading of the reference pan and sample pan. For dynamic measurements, the sample and reference pan were loaded at 40 °C. All measurments were carried out under N_2 flow (50 mL min^{-1}).

Once the reaction was complete, the pan was recovered and reweighed to confirm no mass loss had occurred during the reaction. Using a needle, the pan was carefully pierced and soaked in deuterated chloroform containing benzoic acid (catalyst quenching agent, *ca* 10 equiv.) and mesitylene (NMR spectroscopic standard, 10.0 µL, 0.071 mmol). After recording the NMR spectrum, the sample was dried and diluted in THF (ca 1 mL) for SEC analysis.

For determination of activation parameters via isothermal methods, isotherms were held at temperatures between 80-150 °C until completion of the reaction.

2.2 Polymerization Method with Aliquot Analysis

In a glovebox, dVL (2.00 g, 20.0 mmol, 100 equiv.) was added to a vial containing $Sn(Oct)_2$ (80.9 mg, 0.200 mmol, 1.00 equiv.) and BnOH (21.6 mg, 0.200, 1.00 equiv.). The solution was divided equally between 8 vials. The vials were heated to the reaction temperature (100 °C) and individually quenched at pre-determined time intervals using benzoic acid. Conversion and molar mass of the polymer were determined using ^1H NMR spectroscopy and SEC.

2.3 Polymerization Method using *in-situ* ATR-IR Spectroscopy

In a glovebox, dVL was added (2.00 g, 20.0 mmol, 100 equiv.) to a Schlenk-tube containing $Sn(Oct)_2$ (80.9 mg, 0.200 mmol, 1.00 equiv.) and BnOH (21.6 mg, 0.200 mmol, 1.00 equiv.). Under a flow of N_2, the Schlenk-tube was fitted with a ATR-IR probe were then transferred to a preheated oil bath (100 °C). The carbonyl resonances of the monomer and polymer was monitored until the reaction reached completion. Once complete, the vessel was cooled to room temperature and the catalyst quenched through addition of benzoic acid. Final conversion was determined using ^1H NMR spectroscopy and molar mass was determined by SEC.

2.4 Estimation of Activation Parameters using Dynamic Heating Rates

Determination of activation parameters using dynamic heating methods was conducted according to either the Borchardt-Daniels (ASTM E2041, single ramp) or Flynn-Wall-Ozawa method (ASTM E698, multi ramp) with heating rates between 2 and 10 °C min^{-1}. Data for dynamic analysis was processed using the Trios Kinetics software package.

2.4.1 Borchardt-Daniles Method (ASTM E2041-23)

Following Borchardt-Daniels method (ASTM E2041-23):

Assuming that the reaction follows first order kinetics, the rate of reaction can be expressed as:

$$\frac{d\alpha}{dt} = k(1-\alpha)$$

Equation S1

Where α is the fractional conversion, $\frac{d\alpha}{dt}$ is the reaction rate, *k* is the rate constant at a given temperature, t is time.

Taking the natural logarithm of both sides gives:

$$ln\frac{d\alpha}{dt} = lnk + ln(1-\alpha)$$

Equation S2

Equation S2 can be rearranged to:

$$lnk = ln\frac{d\alpha}{dt} - ln(1-\alpha)$$

Equation S3

The rate and fractional conversion is determined from the DSC exotherm (Fig. S1). Here, the determination was taken using 20 segments of the curve, evenly spaced by temperature, between 5% conversion and the conversion at maximum rate (Fig S2 and Table S1)..

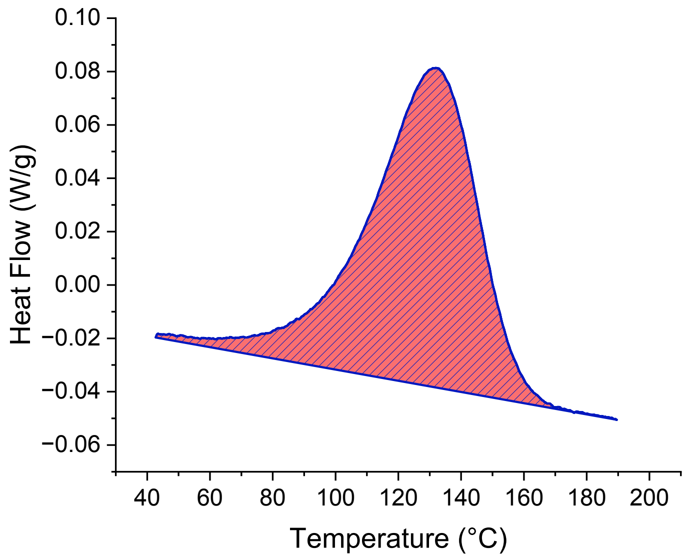

Figure S1. Heatflow *vs* temperature for the ROP of dVL conducted under a heating rate of 2 °C min^{-1} between 40 – 200 °C ([Sn(Oct)$_2$]$_0$:[BnOH]$_0$:[dVL]$_0$ = 1:1:100, in neat dVL. [dVL]$_0$ = 9.98 M). Shade shows the area where the integral was taken to generate figure S2.

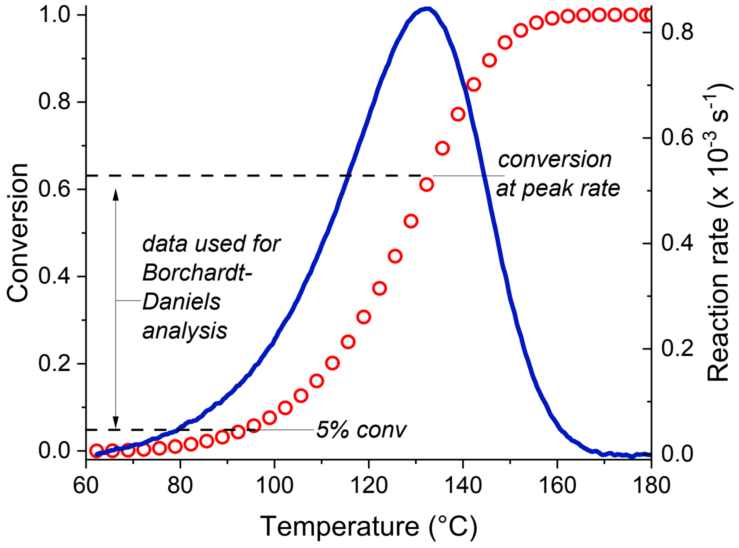

Figure S2. Conversion (red circles, left y axis) and reaction rate (blue line, right y axis) *vs* temperature for the ROP of dVL. Reaction conditions: heating rate of 2 °C min^{-1}, 40 – 200 °C, $[Sn(Oct)_2]_0$:$[BnOH]_0$:$[dVL]_0$ = 1:1:100, neat ($[dVL]_0$ = 9.98 M). Conversion was determined from the normalized integral of the heatflow *vs* time data in figure S1. The reaction rate is equivalent to the normalized heat flow in figure S1.

Table S1. Temperature, fraction conversion and rate determined at 20 evenly spaced sections between 5 and 65% conversion of dVL ROP conducted a heating rate of 2 °C min^{-1} between 40 – 200 °C.

Temp. (°C)[a]	1/Temp (K^{-1})	Fractional conversion, α[a]	Rate (s^{-1})[a]	ln(1-α)	ln k[b]
93.95	0.00272	0.05	1.46533E-4	-0.05129	-8.77696
96.05	0.00271	0.05999	1.6926E-4	-0.06187	-8.62221
98.15	0.00269	0.07121	1.93803E-4	-0.07387	-8.4748
100.25	0.00268	0.08426	2.21292E-4	-0.08802	-8.32801
102.35	0.00266	0.09925	2.52517E-4	-0.10453	-8.17951
104.45	0.00265	0.11616	2.8659E-4	-0.12348	-8.03398
106.55	0.00263	0.13538	3.26368E-4	-0.14547	-7.88202
108.65	0.00262	0.15716	3.66525E-4	-0.17097	-7.74047
110.75	0.0026	0.18164	4.12015E-4	-0.20045	-7.594
112.85	0.00259	0.20898	4.56898E-4	-0.23444	-7.45661
114.95	0.00258	0.23984	5.10818E-4	-0.27423	-7.30527
117.05	0.00256	0.27326	5.64097E-4	-0.31918	-7.1611
119.15	0.00255	0.31056	6.17955E-4	-0.37188	-7.01721
121.25	0.00254	0.35111	6.71662E-4	-0.43249	-6.87327
123.35	0.00252	0.39526	7.2416E-4	-0.50295	-6.72755
125.45	0.00251	0.44229	7.71317E-4	-0.58391	-6.5835
127.55	0.0025	0.49202	8.07068E-4	-0.67731	-6.44479
129.65	0.00248	0.54375	8.34108E-4	-0.78471	-6.30444
131.76	0.00247	0.59702	8.4425E-4	-0.90888	-6.16818
133.86	0.00246	0.65	8.38415E-4	-1.04982	-6.03418

Reaction conditions: $[Sn(Oct)_2]_0$: $[BnOH]_0$: $[dVL]_0$ = 1: 1: 100 in neat dVL. $[dVL]_0$ = 9.98 M. [a] Determined from 20 evenly spaced segments in area indiciated in figure S2 [b] $\ln k = \ln\frac{d\alpha}{dt} - \ln(1-\alpha)$

Assuming the reaction follows arrenhius behaviour:

$$k = A\, e^{-\frac{\Delta H^{\ddagger}}{RT}}$$
Equation S4

Where T is temperature, A is the pre-exponential factor, $\Delta H^{\ddagger}$ is the activation energy and R is the gas constant.

Taking the natural logarithm gives:

$$lnk = \ln(A) - \frac{\Delta H^{\ddagger}}{RT}$$
Equation S5

Using the estimated values of *k* (Table S1), a plot of ln*k* *vs* 1/T yields a straight line. The $\Delta H^{\ddagger}$ and A are estimated from the slope and intercept, respectively.

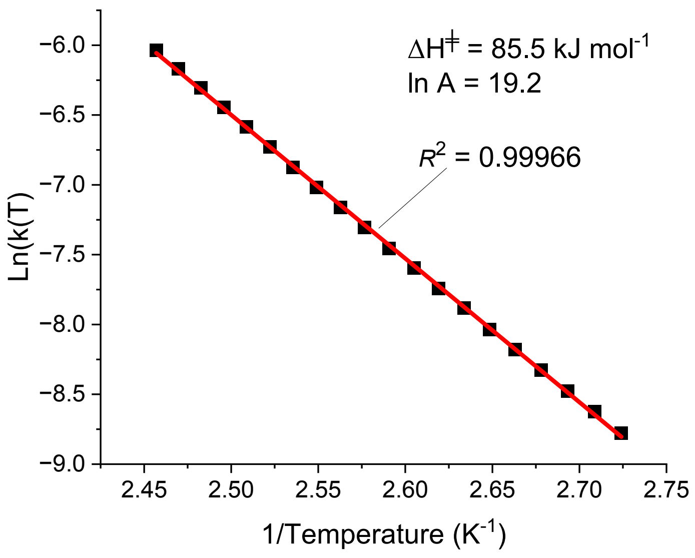

Figure S3. Plot of ln k *vs* 1/temperature for for the ROP of dVL. Reaction conditions: heating rate of 2 °C min^{-1}, 40 – 200 °C, [Sn(Oct)$_2$]$_0$:[BnOH]$_0$:[dVL]$_0$ = 1:1:100, neat ([dVL]$_0$ = 9.98 M). Data taken from Table S1.

2.4.2 Activation Parameters using Multi-heating-rate Method

Following the Flynn-Wall-Ozawa method (ASTM E698-05):

The fractional conversion and rate can be determined by integrating and normalising the DSC exotherms at different heating rates to give the data shown in Figures S4 and 5:

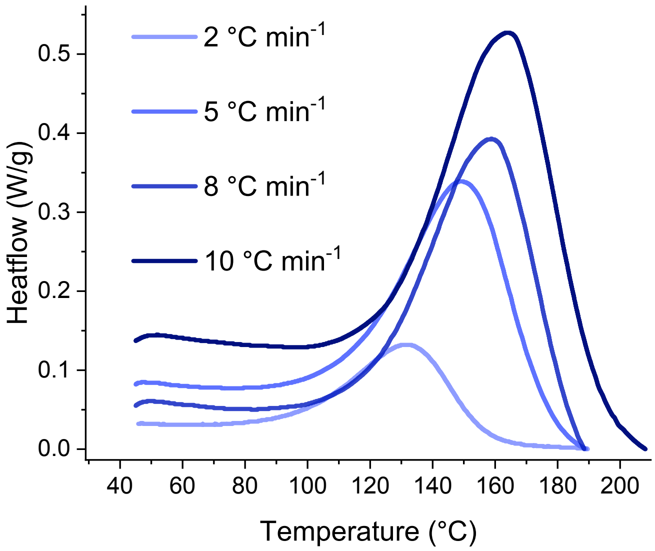

Figure S4. Plot of heatflow *vs* temperature for the ROP of dVL. Reaction conditions: heating rates of 2, 5, 8 and 10 °C min^{-1}, 40 – 220 °C, $[Sn(Oct)_2]_0$:$[BnOH]_0$:$[dVL]_0$ = 1:1:100, neat ($[dVL]_0$ = 9.98 M). Thermograms were integrated as shown in figure S1.

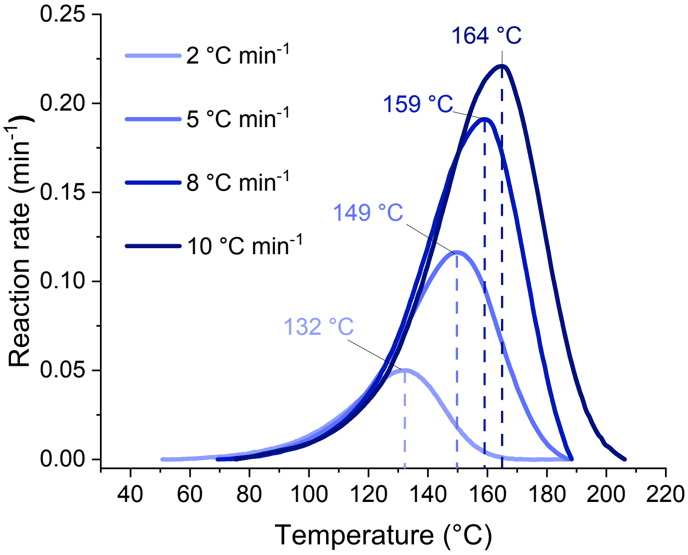

Figure S5. Plot of reaction rate *vs* temperature for ROP of dVL. Reaction conditions: heating rates of 2, 5, 8 and 10 °C min^{-1}, 40 – 220 °C, $[Sn(Oct)_2]_0$:$[BnOH]_0$:$[dVL]_0$ = 1:1:100, neat ($[dVL]_0$ = 9.98 M). The reaction rate is equivalent to the normalized heat flow in figure S4. The peak temperature for each heating rate is shown.

Table S2. Table showing heating rate, peak temperature taken from figure S5 for the ROP of dVL conducted under dynamic heating between 40 – 200 °C

Heating rate, β (°C min^{-1})	Peak temperature (°C)	1/Tempeature (x 10^{-3} K^{-1})	Log β
2	132	2.47	0.3
5	149	2.37	0.7
8	159	2.31	0.9
10	164	2.28	1

Reaction conditions: $[Sn(Oct)_2]_0$: $[BnOH]_0$: $[dVL]_0$ = 1: 1: 100 in neat dVL. $[dVL]_0$ = 9.98 M.

The log of the heating rate, β, was plotted against reciprocal temperature to give:.

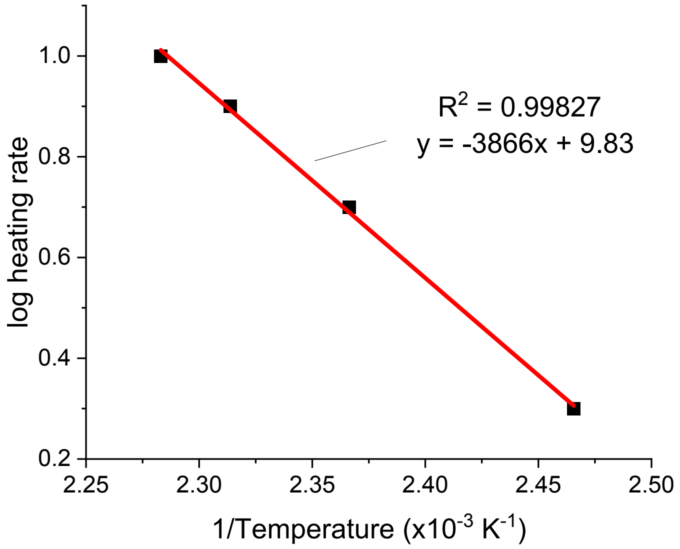

Figure S6. Plot of Log(heating rate) vs 1/temperature for the ROP dVL. Reaction conditions: heating rates of 2, 5, 8 and 10 °C min^{-1},40 – 220 °C, $[Sn(Oct)_2]_0$:$[BnOH]_0$:$[dVL]_0$ = 1:1:100, neat, ($[dVL]_0$ = 9.98 M).

An initial $\Delta H^{\ddagger}$ is estimated from the gradient of figure S6 using:

$$\Delta H^{\ddagger}(est1) = \ -2.19\ \times gradient\ \times R$$

Equation S6

$$= 70394.5\ \mathrm{J\ mo}l^{-1}$$

Where R is the gas constant.

The $\Delta H^{\ddagger}$ is refined as follows:

To calculate the correction factor, *X*, use:

$$X = \frac{\Delta H^{\ddagger}(est1)}{RT}$$

Equation S7

Using $\Delta H^{\ddagger}$(est1), the peak temperature at the maxiumum heating rate and equation S7 gives:

$$X1 = \frac{\Delta H^{\ddagger}(est1)}{RT} \approx 20$$

Using Table X2.1 given in ASTM0698-05, X1 corresponds to a *D* = 1.10 (D1)

The ΔH‡ is further refined using:

$$\Delta H^{\ddagger}(est2) = \frac{-2.303 \times R \times gradient}{D1}$$

Equation S8

$$= 67296 \text{ J mo}l^{-1}$$

Using ΔH‡(est2) the peak temperature at the maximum heating rate and equation S7 gives:

$$X2 = \frac{\Delta H^{\ddagger}(est2)}{RT} \approx 19$$

Using Table X2.1 given in ASTM0698-05, this estimate of X2 corresponds to a *D* = 1.1053 (*D2)*

The ΔH‡(final) is given by:

$$\Delta H^{\ddagger}(final) = \frac{-2.303 \times R \times gradient}{D2}$$

$$= 66.9 \text{ J mo}l^{-1}$$

The A value was calculated using:

$$A = \frac{\beta \times \Delta H^{\ddagger} \times e^{\Delta H^{\ddagger}/RT}}{RT^2}$$

Equation S9

$$= 699485\ s$$

$$\ln A = 13.5$$

3. Additional information

3.1 Benchmarking DSC kinetics

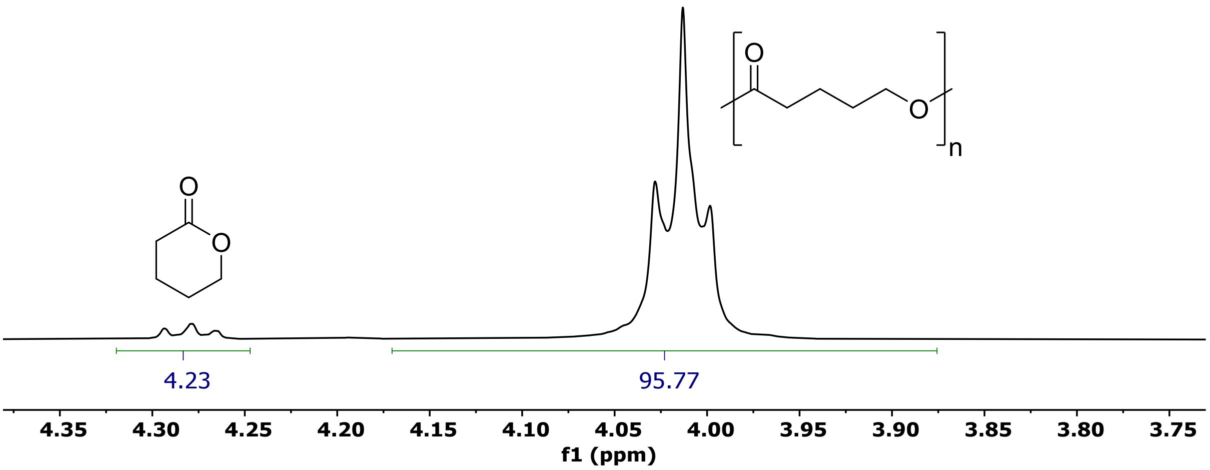

Figure S7. ^1H **NMR spectrum of PVL (dVL ROP) conducted in a DSC. Reaction conditions: 100 °C, $[Sn(Oct)_2]_0$:$[BnOH]_0$:$[dVL]_0$ = 1:1:100, in neat dVL ($[dVL]_0$ = 9.98 M). The monomer conversion was used to determine [PVL] as [PVL] = $[dVL]_0$ - $[dVL]_t$.**

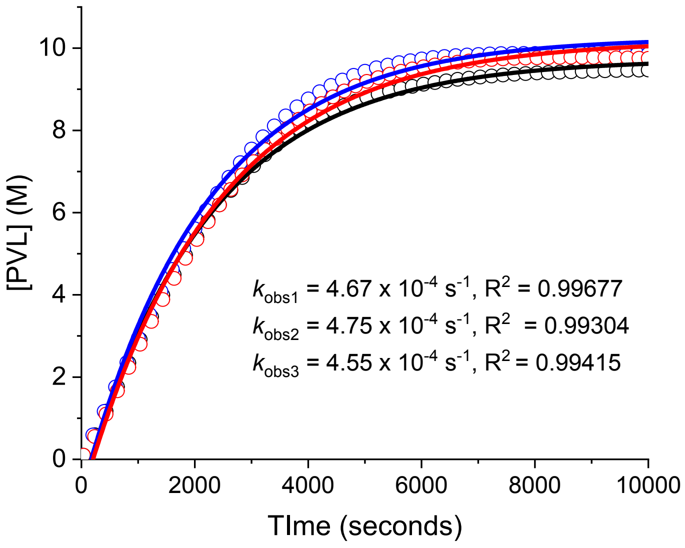

Figure S8. Plot of [PVL] vs time showing repeat, independent experiments (red, blue and black circles). The final conversion of dVL was determined by ^1H **NMR spectroscopy (Fig. S7) and used to calculate the [PVL] vs time profile. The red, blue and black lines each show the first-order exponential fit to the data which was used to extract k_{obs} values.**

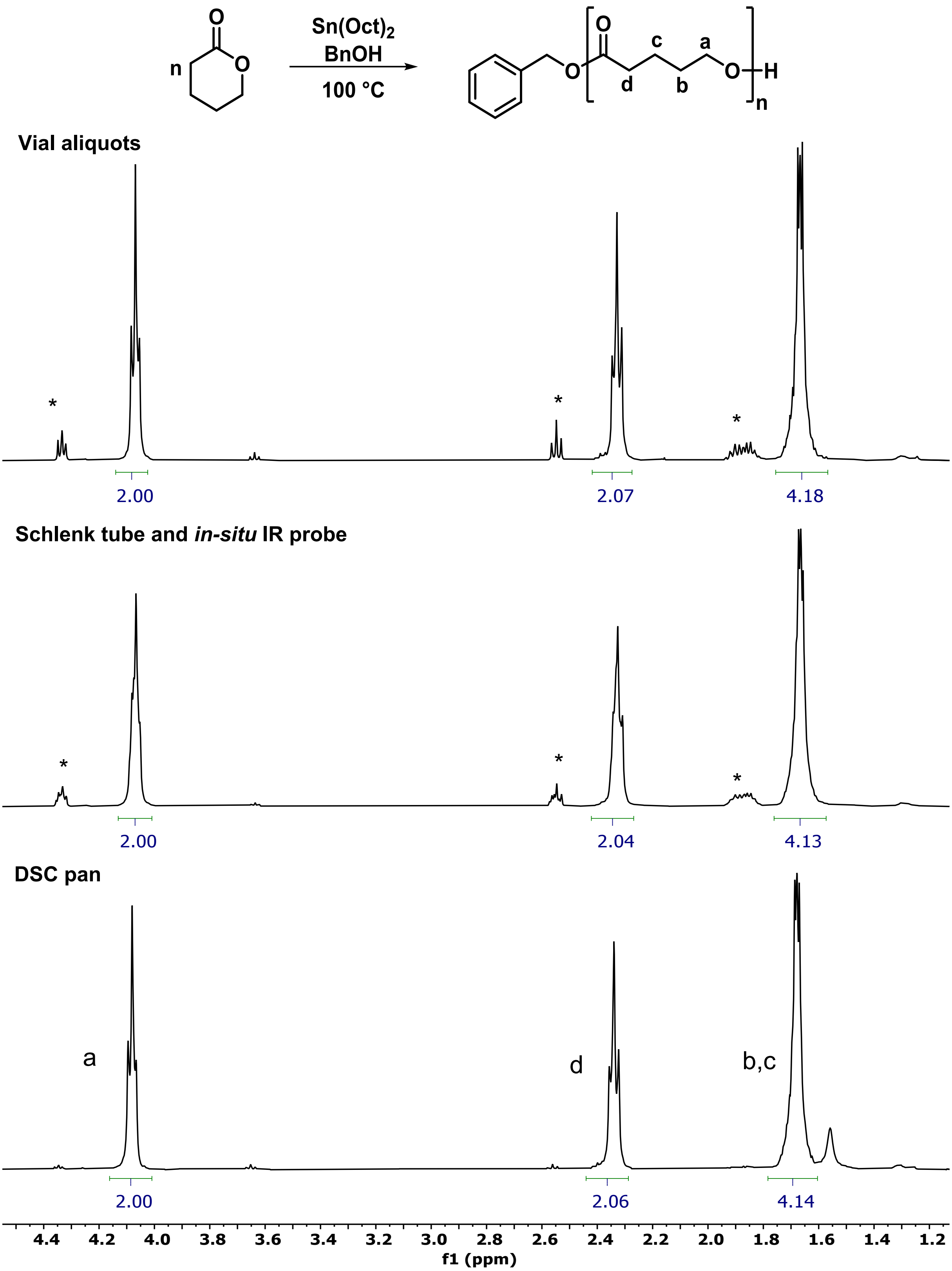

Figure S9. Stacked crude ^{1}H NMR spectra from dVL ROP conducted in vial (top), Schlenk (middle) and DSC pan (bottom). Reactions conditions: 120 °C, $[Sn(Oct)_2]_0:[BnOH]_0:[dVL]_0$ = 1:1:100, in neat dVL ($[dVL]_0$ = 9.98 M). * indicates residual monomer

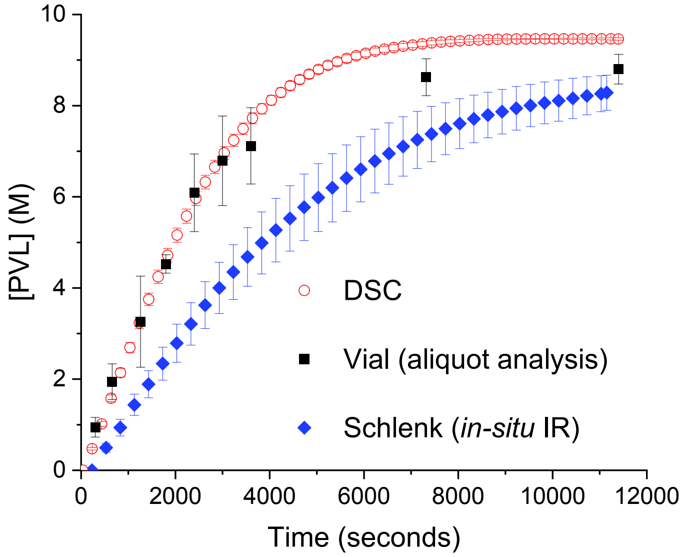

Figure S10. Plot of [PVL] *vs* time for the ROP of dVL. Reaction conditions: 100 °C, $[Sn(Oct)_2]_0$:$[BnOH]_0$:$[dVL]_0$ = 1:1:100, in neat monomer ($[dVL]_0$ = 9.98 M). (Table 1, entries 1 – 2 and Table S3). Reactions were monitored by DSC (red, conducted in DSC), ^{1}H NMR spectroscopy (black, conducted in vial) and in-situ IR spectroscopy (red, conducted in Schlenk). The error bars are the standard deviation of the mean taken from three repeat runs (note: error on DSC runs is significantly smaller).

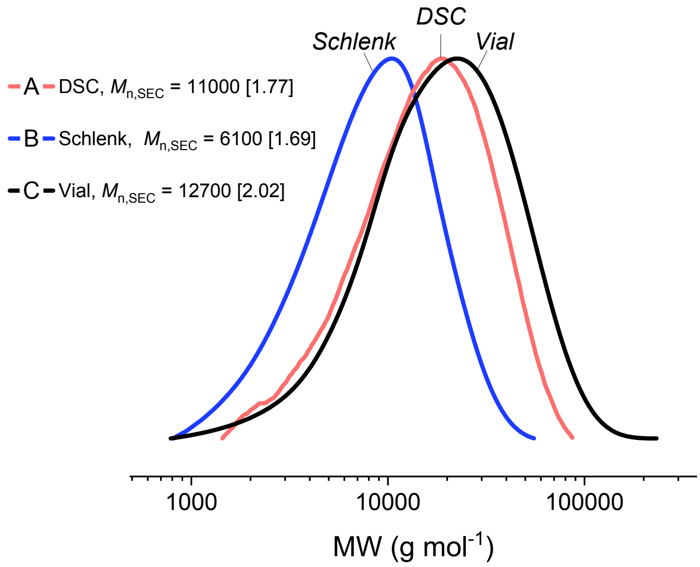

Figure S11. SEC chromatograms of PVL formed in DSC pan (red), vial (black) or Schlenk tube (blue). Reaction conditions: $[Sn(Oct)_2]_0$:$[BnOH]_0$:$[dVL]_0$ = 1:1:100, in neat monomer ($[dVL]_0$ = 9.98 M).

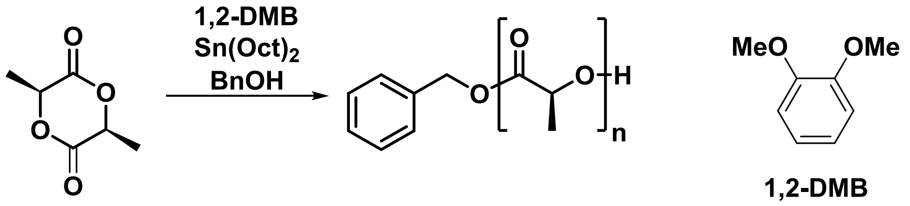

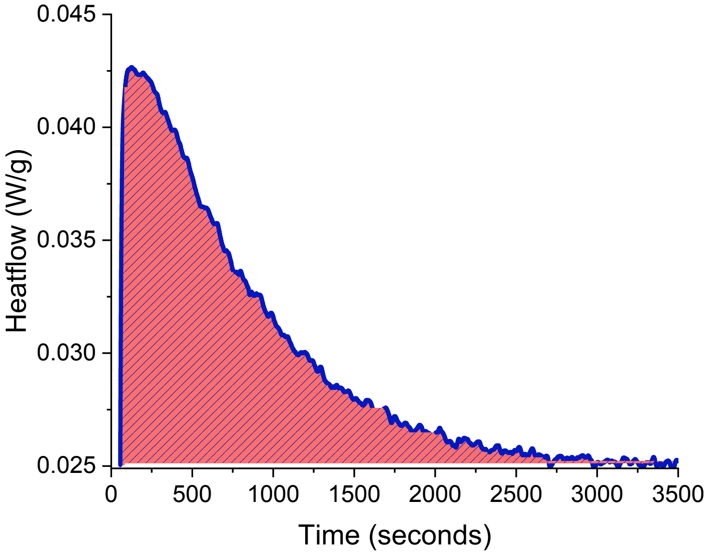

Figure S12. Plot of heatflow vs time for the ROP of LLA. Reaction conditions: 120 °C, $[Sn(Oct)_2]_0$:$[BnOH]_0$:$[LLA]_0$ = 1:1:100, $[LLA]_0$ = 1.0 M in 1,2-dimethoxybenzene. (Table 1, entry 3). The shaded area shows the region where the integral was taken to generate figure S13.

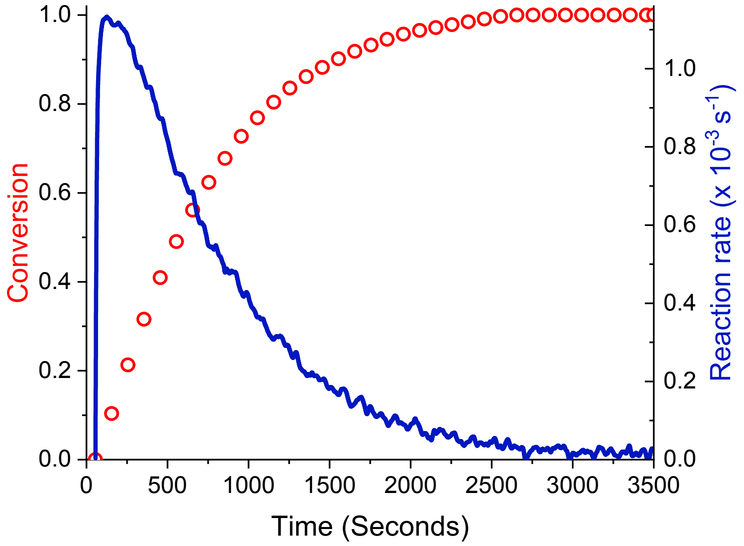

Figure S13. Plot of PLLA conversion (red circles, left y axis) and reaction rate (blue line, right y axis) *vs* time for the ROP of LLA. Reaction conditions: 120 °C, $[Sn(Oct)_2]_0$:$[BnOH]_0$:$[LLA]_0$ = 1:1:100, in 1,2-dimethoxybenzene, $[LLA]_0$ = 1.0 M. Conversion was determined from the normalized integral of the heatflow *vs* time data in figure S12. The reaction rate is equivalent to the normalized heat flow in figure S12.

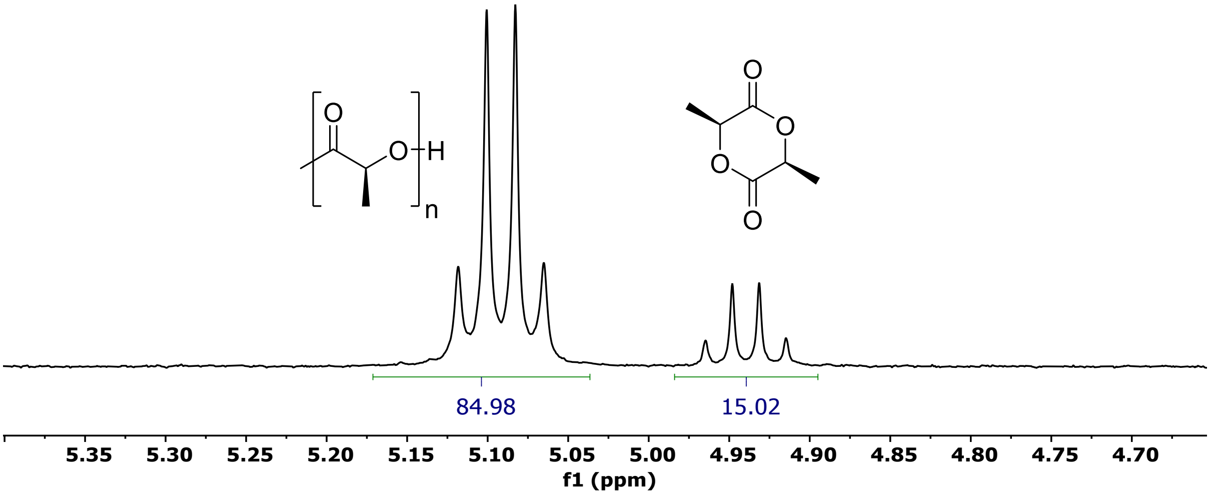

Figure S14. ^{1}H NMR spectrum of PLLA (LLA ROP) conducted in a DSC. Reaction conditions: 120 °C, $[Sn(Oct)_2]_0$:$[BnOH]_0$:$[LLA]_0$ = 1:1:100, $[LLA]_0$ = 1.0 M in 1,2-dimethoxybenzene.The monomer conversion (83%) was used to determine [PLLA] as [PLLA] = $[LLA]_0$ - $[LLA]_t$

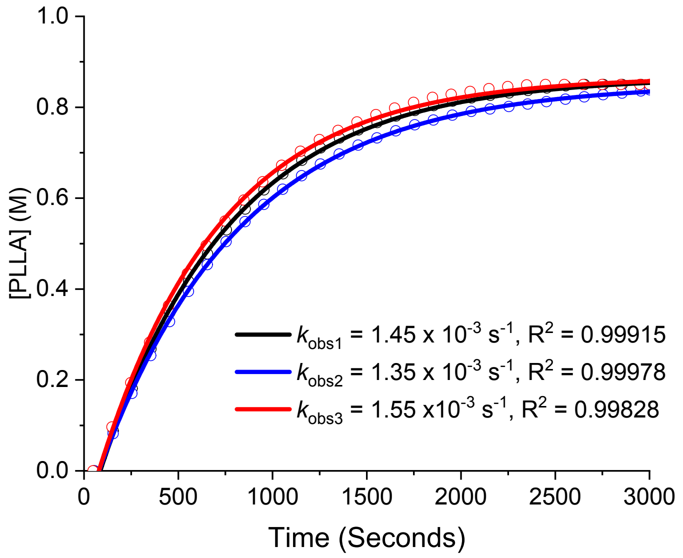

Figure S15. Plots of [PLLA] vs time showing repeat, independent experiments (red, blue and black circles). The final conversion of LLA was determined by ^{1}H NMR spectroscopy (Fig. S14) which was used to calculate the [PLLA] vs time profile. The red, blue and black lines each show the first-order exponential fit to the data which was used to extract k_{obs} values

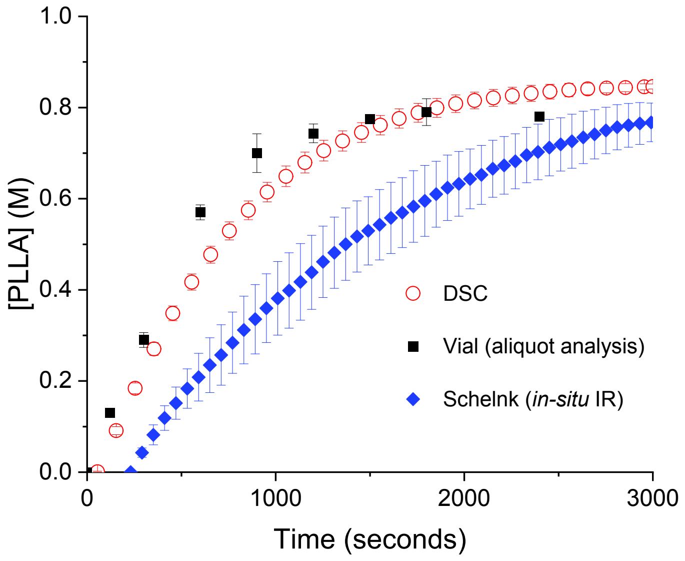

Figure S16. Plots of [PLLA] *vs* time for the ROP of LLA. Reaction conditions: 120 °C, $[Sn(Oct)_2]_0$:$[BnOH]_0$:$[LLA]_0$ = 1:1:100, $[LLA]_0$ = 1.0 M in 1,2-dimethoxybenzene. (Table 1, entries 3 – 4 and Table S3). Reactions were monitored by DSC (red, conducted in DSC), ^{1}H NMR spectroscopy (black, conducted in vial) and in-situ IR spectroscopy (blue, conducted in Schlenk). The error bars are the standard deviation of the mean taken from three repeat runs (note: error on DSC runs is significantly smaller).

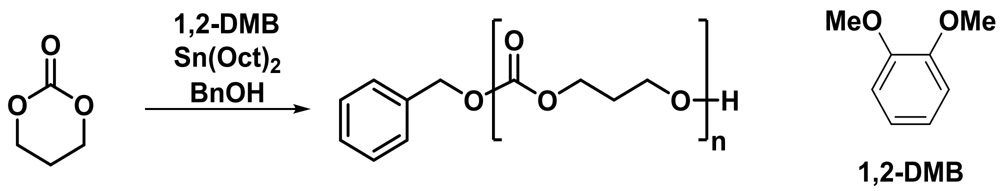

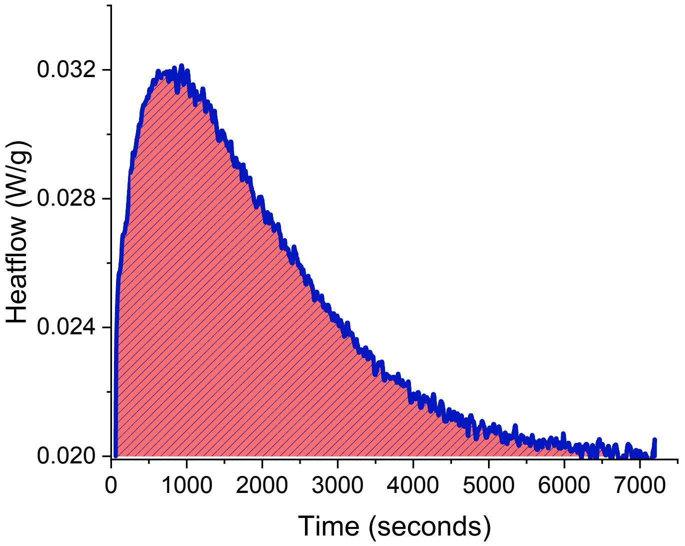

Figure S17 Plots of heatflow *vs* time for ROP of TMC. Reaction conditions: 120 °C, $[Sn(Oct)_2]_0$:$[BnOH]_0$:$[TMC]_0$ = 1:1:100, $[TMC]_0$ = 2.0 M in 1,2-dimethoxybenzene. (Table 1, entries 5). The shaded area shows the region where the integral was taken to generate figure S18.

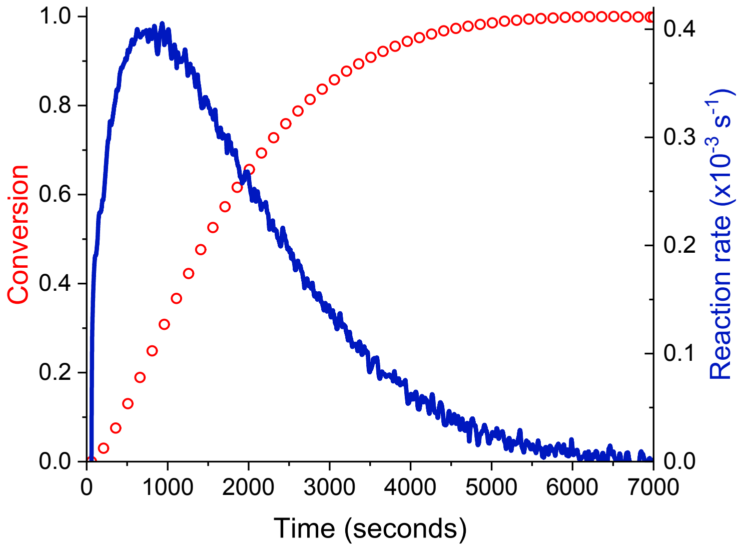

Figure S18. Plot of PTMC conversion (red circles, left y axis) and reaction rate (blue line, right y axis) *vs* time. Reaction conditions: 120 °C, $[Sn(Oct)_2]_0$:$[BnOH]_0$:$[TMC]_0$ = 1:1:100, $[TMC]_0$ = 2.0 M in 1,2-dimethoxybenzene. Conversion was determined from the normalized integral of the heatflow *vs* time data in figure S17. The reaction rate is equivalent to the normalized heat flow in figure S17.

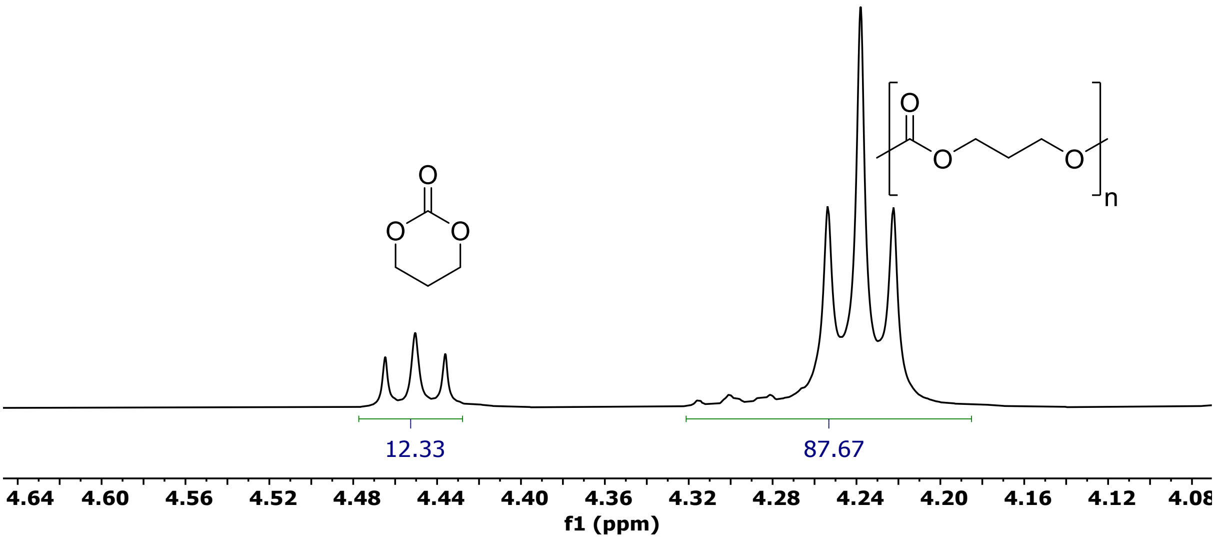

Figure S19. ^{1}H NMR spectrum of PTMC (TMC ROP) formed in a DSC. Reaction conditions: 120 °C, $[Sn(Oct)_2]_0$:$[BnOH]_0$:$[TMC]_0$ = 1:1:100, $[TMC]_0$ = 2.0 M in 1,2-dimethoxybenzene. The monomer conversion (88%) was used to determine [PTMC] as [PTMC] = $[TMC]_0$ – $[TMC]_t$ figure S20.

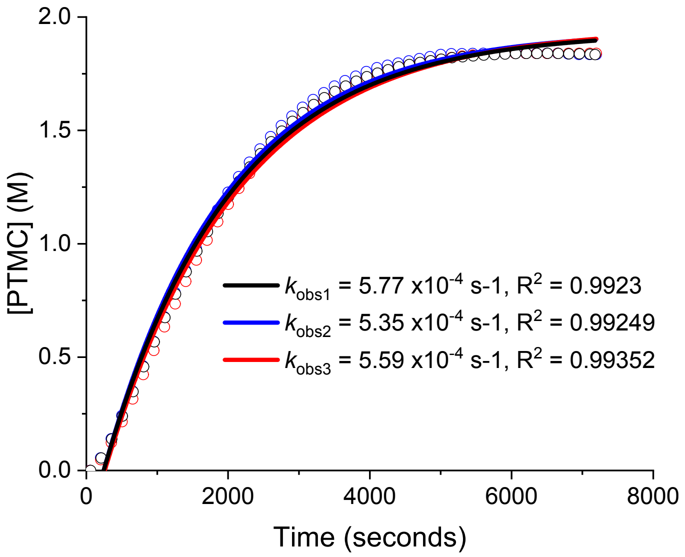

Figure S20. Plots of [PTMC] vs time showing repeat, independent experiments (red, blue and black circles). The final conversion of TMC was determined by ^{1}H NMR spectroscopy (Fig. S19) which was used to calculate the [PTMC] vs time profile. The red, blue and black lines each show the first-order exponential fit to the data which was used to extract k_{obs} values

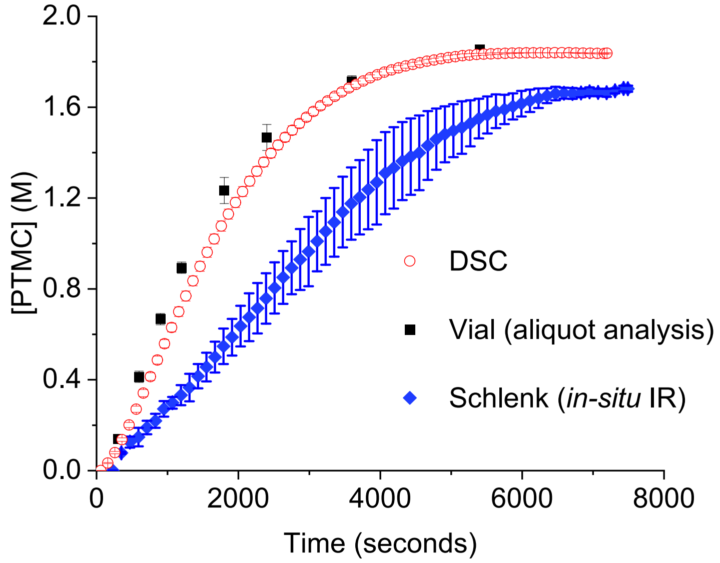

Figure S21. Plots of [PTMC] vs time. Reaction conditions: 120 °C, $[Sn(Oct)_2]_0$:$[BnOH]_0$:$[TMC]_0$ = 1:1:100, $[TMC]_0$ = 2.0 M in 1,2-dimethoxybenzene (Table 1, entries, 5 – 6). Reactions were monitored by DSC (red, conducted in DSC), ^{1}H NMR spectroscopy (black, conducted in vial) and in-situ IR spectroscopy (blue, conducted in Schlenk). The error bars are the standard deviation of the mean taken from three repeat runs (note: error on DSC reactions is significantly smaller than vial or Schlenk reactions).

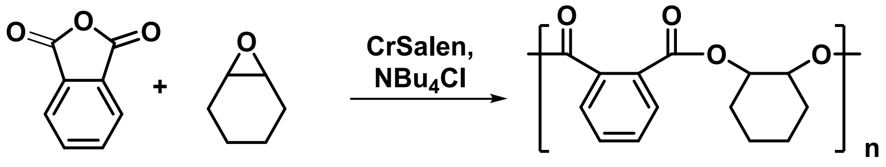

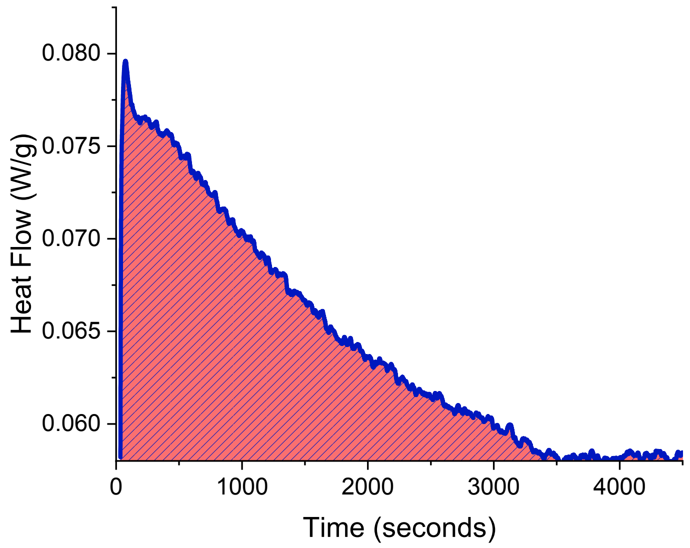

Figure S22. Plot of heatflow *vs* time for the ROCOP of CHO and PA at 85 °C $[(salcy)CrCl]_0$:$[PPNCl]_0$:$[PA]_0$:$[CHO]_0$ = 1: 50: 2000 in neat CHO ($[CHO]_0$ = 9.5 M, Table 1, entry 5). The shaded area shows the region where the integral was taken to generate figure S23. *Note these loadings of PA/CHO were chosen to maximise the polymerization rate, as the reaction is first order in CHO and zero-order in anhydride, and ensure complete solubility of the PA monomer at room temperature*.

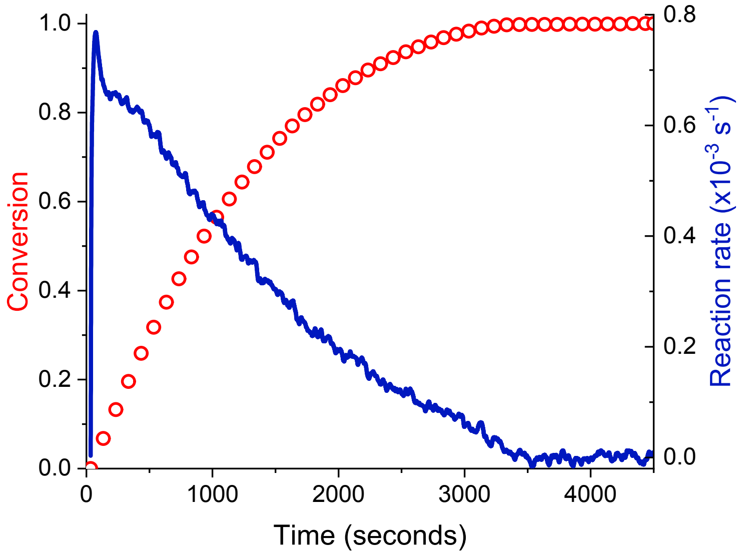

Figure S23. Plots of PA conversion (red circles, left y axis) and reaction rate (blue line, right y axis) *vs* time, for the ROCOP of PA and CHO. Reaction conditions: 85 °C, $[(salcy)CrCl]_0$:$[PPNCl]_0$:$[PA]_0$:$[CHO]_0$ = 1: 50: 2000 in neat CHO ($[CHO]_0$ = 9.5 M). Conversion was determined from the normalized integral of the heatflow *vs* time data in figure S22. The reaction rate is equivalent to the normalized heat flow in figure S22.

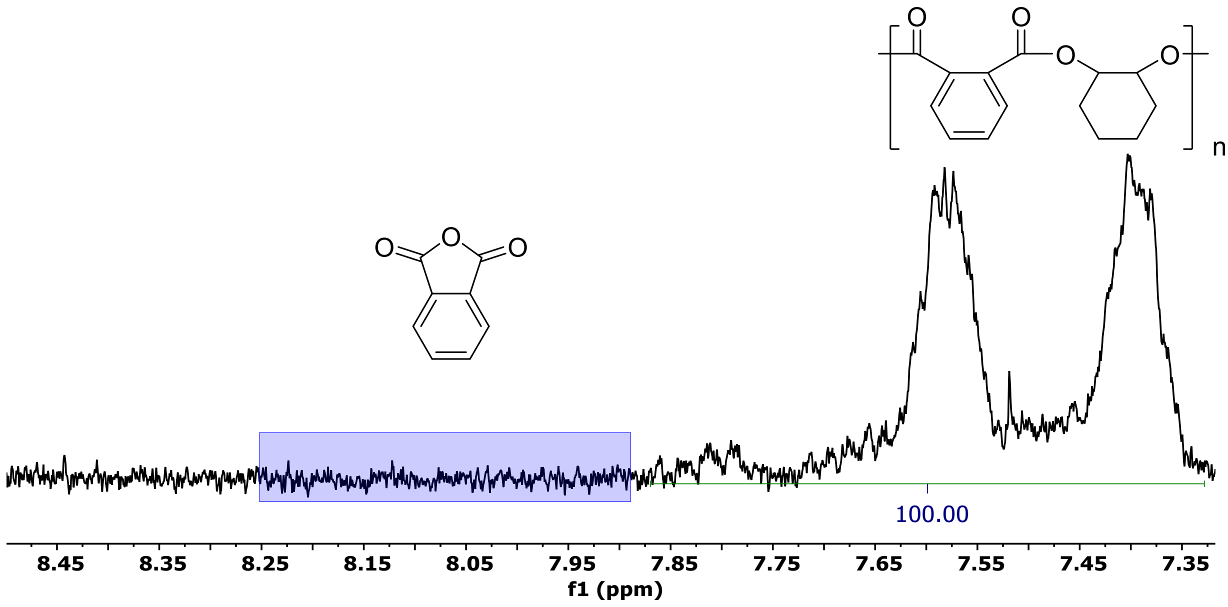

Figure S24. ^1H **NMR spectrum of P(PA-*alt*-CHO) (PA/CHO ROCOP) conducted in a DSC. Reaction condtions: 85 °C, $[(salcy)CrCl]_0$:$[PPNCl]_0$:$[PA]_0$:$[CHO]_0$ = 1: 50: 2000, in neat CHO ($[CHO]_0$ = 9.5 M). The monomer conversion (100%) was used to determine [P(PA-*alt*-CHO)] as [P(PA-*alt*-CHO)] = $[PA]_0$ – $[PA]_t$ (note, shaded area shows region PA resonances appear).**

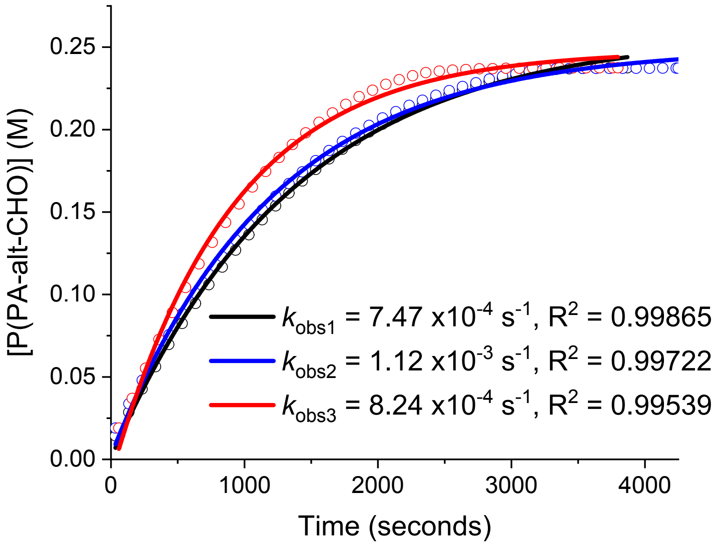

Figure S25. Plots of [P(PA-*alt*-CHO] vs time showing repeat, independent experiments (red, blue and black circles). The final conversion of PA was determined by ^1H NMR spectroscopy (Fig. S24) which was used to calculate the [P(PA-*alt*-CHO)]] vs time profile. The red, blue and black lines each show the first-order exponential fit to the data which was used to extract k_{obs} values.

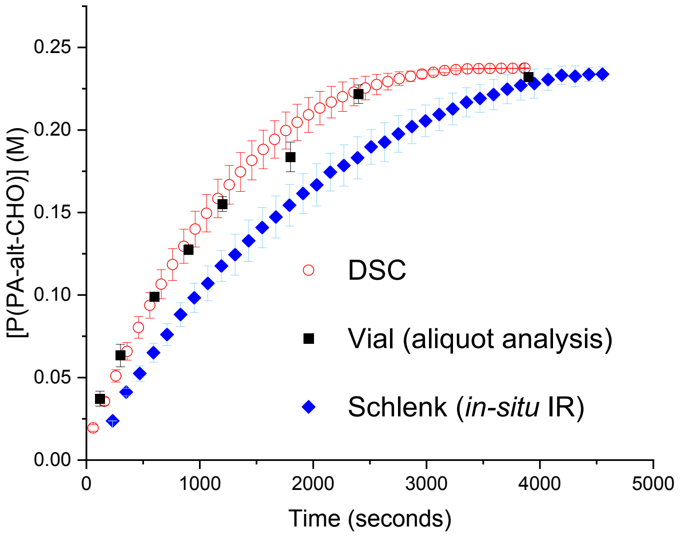

Figure S26. Plots of [P(PA-*alt*-CHO)] vs time. Reaction conditions: 85 °C, $[(salcy)CrCl]_0$:$[PPNCl]_0$:$[PA]_0$:$[CHO]_0$ = 1:1:50:2000, in neat CHO ($[CHO]_0$ = 9.5 M, Table 1, entries 5 – 6 and Table S3). Reactions were monitored by DSC (red, conducted in DSC), ^{1}H NMR spectroscopy (black, conducted in vial) and in-situ IR (blue, conducted in Schlenk). The error bars are the standard deviation of the mean taken from three repeat runs (note: error on DSC reactions is significantly smaller than vial or Schlenk reactions).

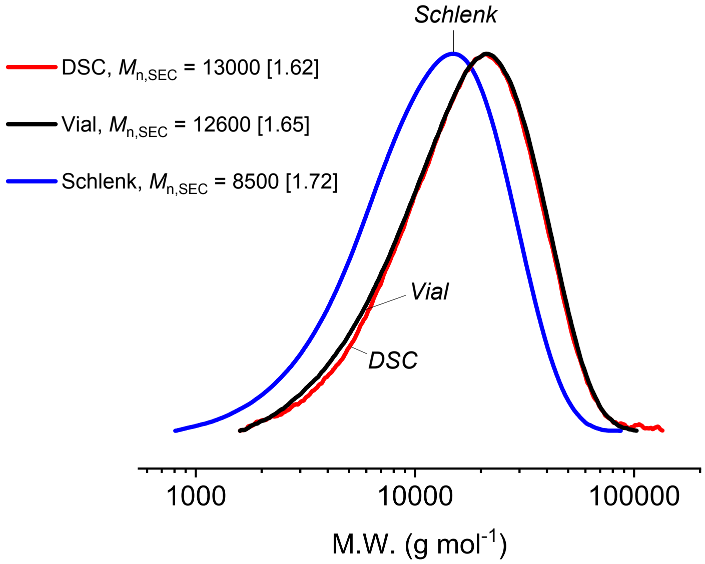

Figure S27. SEC chromatograms of PTMC formed in DSC (red), vial (black) or Schlenk tube (blue). Reaction conditions: 120 °C, $[TMC]_0$:$[BnOH]_0$:$[Sn(Oct)_2]_0$ 1: 1: 100, $[TMC]_0$ = 2 M in 1,2-dimethoxybenzene.

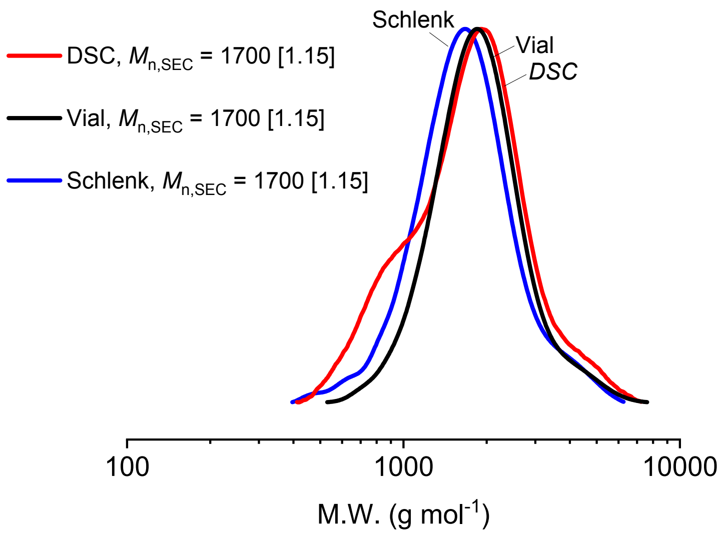

Figure S28. SEC chromatograms of P(PA-*alt*-CHO) formed in DSC (red), vial (black) or Schlenk tube (blue). Reaction conditions: 85 °C, $[(salcy)CrCl]_0$:$[PPNCl]_0$:$[PA]_0$:$[CHO]_0$ 1: 1: 50: 2000 in neat CHO ($[CHO]_0$ = 9.5 M).

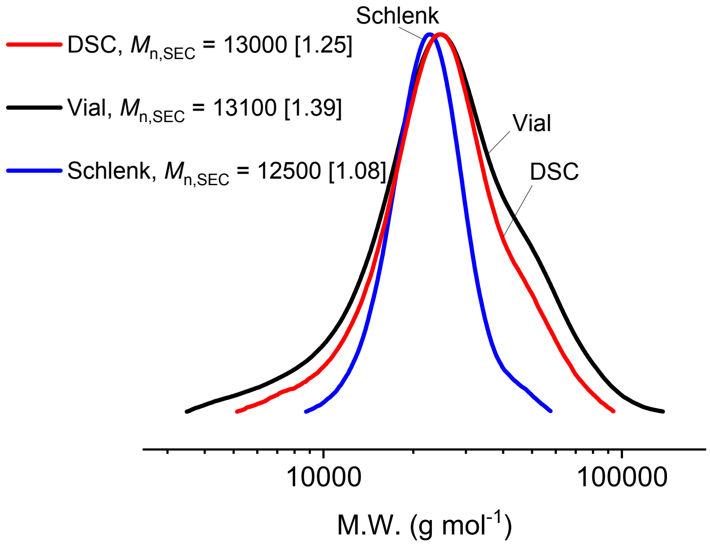

Figure S29. SEC chromatograms of PLLA formed in DSC pan (red), vial (black) or Schlenk tube (blue). Reaction conditions: 120 °C, $[Sn(Oct)_2]_0$:$[BnOH]_0$:$[LLA]_0$ at 1: 1: 100 $[LLA]_0$ = 1 M in 1,2-dimethoxybenzene. Note: correction factor of 0.58 applied.[2]

Table S3. Summary of kinetic data using *in-situ* IR probe.

#	Mon.	Temp. (°C)	k_{obs} (x 10^{-4} s^{-1})[a]	TOF (h^{-1})[b]	Poly. Conv. (%)[c]	$M_{n,SEC}$ (g mol^{-1}) [$Đ_M$][d]	*Overall Mass needed (g)[e]*
1[f]	dVL	100	2.2 (±0.5)	37(±7)	91(±2)	6100 [1.69]	6.00
2[g]	LLA	120	8.6 (±2.1)	133 (±30)	82(±3)	12500 [1.08][h]	6.00
3[i]	TMC	120	2.4 (±0.5)	54 (±5)	90(±3)	8500 [1.72]	6.00
4[j]	PA/ CHO	100	5.1 (±0.9)	125(±9)	>99%	1700 [1.15]	6.00

[a] The rate constants are all obtained by exponential fits to the polymer/monomer conversion *vs* time data. [b] Catalytic Activity obtained as turn-over-frequency, TOF= # moles monomer consumed/# moles catalyst/time. For all experiments the TOF was determined at 33% monomer conversion from the rate constant monitoring. [c] The polymer conversion was determined at the end of the reaction using ^{1}H NMR spectroscopy, with mass balance confirmed by weighing. [d] Polymer molecular weight (Mn) determined by SEC, with a THF eluent and polystyrene standards (PVL, P(PA-*alt*-CHO), PTMC) or with $CHCl_3$ eluent and polystyrene standards (PLLA). [e] The total mass used for the combined triplicate measurements. [f] Polymerization conditions: $[Sn(Oct)_2]_0$:$[BnOH]_0$:$[dVL]_0$ = 1:1:100 at 100 °C, $[dVl]_0$ =bulk (9.98 M) [g] Polymerization conditions $[Sn(Oct)_2]_0$:$[BnOH]_0$:$[LLA]_0$ = 1:1:100 at 120 °C, $[LLA]_0$ = 1.0 M in 1,2-dimethoxybenzene.[h] correction factor of 0.58 applied[2]. [i] Polymerization conditions $[Sn(Oct)_2]_0$:$[BnOH]_0$:$[TMC]_0$ = 1:1:100 at 120 °C, $[TMC]_0$ = 2.0 M in 1,2-dimethoxybenzene. [j] $[(salcy)CrCl]_0$:$[NBu_4Cl]_0$: $[PA]_0$: $[CHO]_0$ = 1: 1: 50: 2000 at 85 °C, $[CHO]_0$ = 9.5 M in neat CHO.

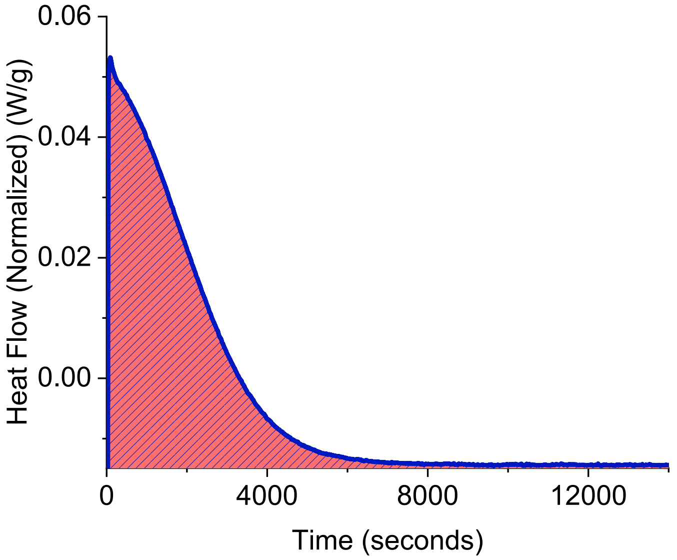

Figure S30. Plot of heatflow vs time for the ROP of dVL. Reaction conditions: 160 °C, $[Sn(Oct)_2]_0$:$[BnOH]_0$:$[dVL]_0$ = 1:1:1000, in neat dVL. $[dVL]_0$ = 9.98 M). The shaded area shows the integral that was used to generate figure S31.

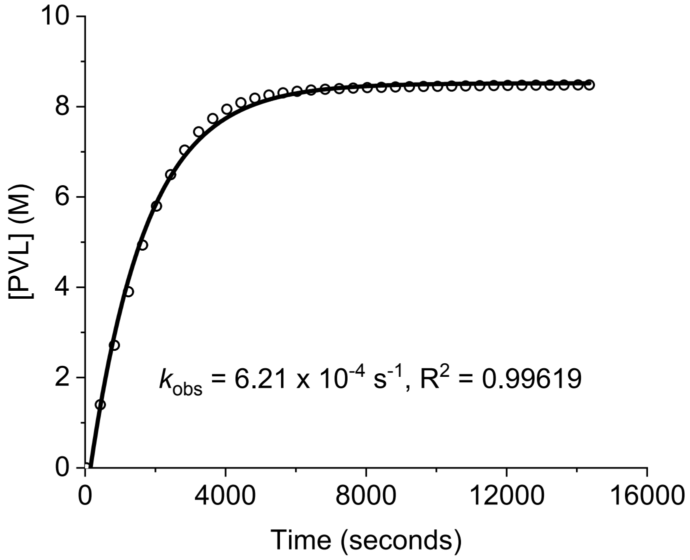

Figure S31. Plot of [PVL] vs time (black circles, 160 °C, $[Sn(Oct)_2]_0$:$[BnOH]_0$:$[dVL]_0$ = 1:1:1000, in neat monomer, $[dVL]_0$ = 9.98 M). The final conversion of dVL was determined by ^1H NMR spectroscopy and used to calculate the [PVL] vs time profile. The black line show the first-order exponential fit to the data which was used to extract k_{obs} values.

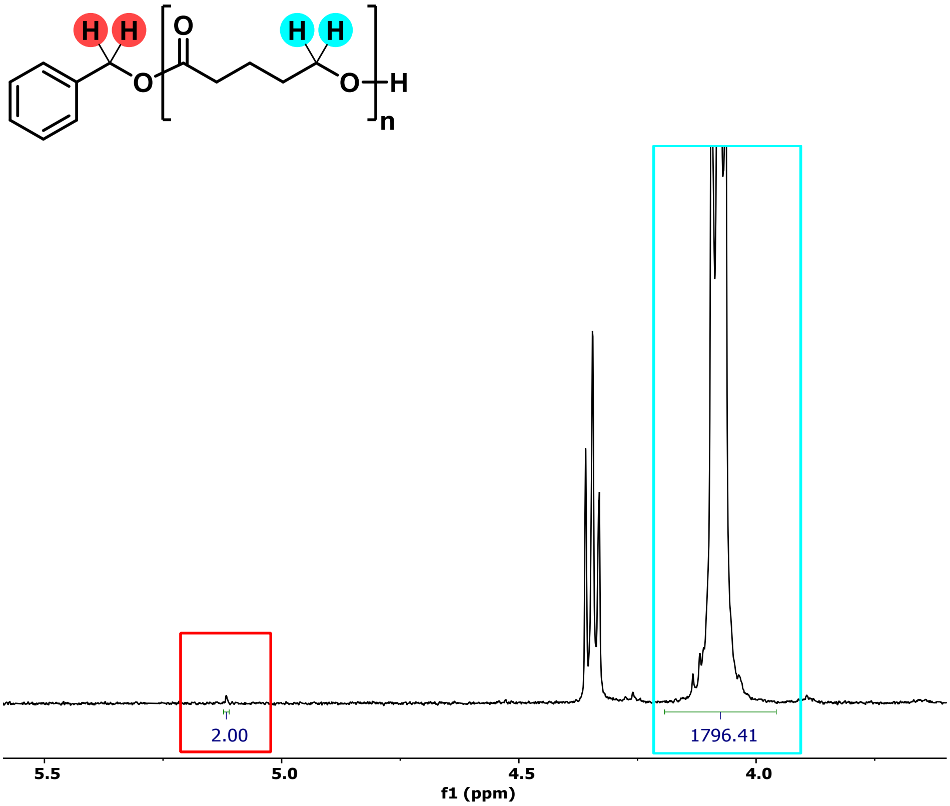

Figure S32. ^1H NMR spectrum of PVL (dVL ROP) conducted in a DSC. Reaction conditions: 160 °C, $[Sn(Oct)_2]_0$:$[BnOH]_0$:$[dVL]_0$ = 1:1:1000, in neat dVL ($[dVL]_0$ = 9.98 M). The highted integrals show the benzyl alcohol end group and PVL repeat unit which gives a degree of polymerization of 898 and $M_{n,NMR}$ of 89,900 g mol^{-1}. *Note residual monomer is detected at 4.25 ppm.*

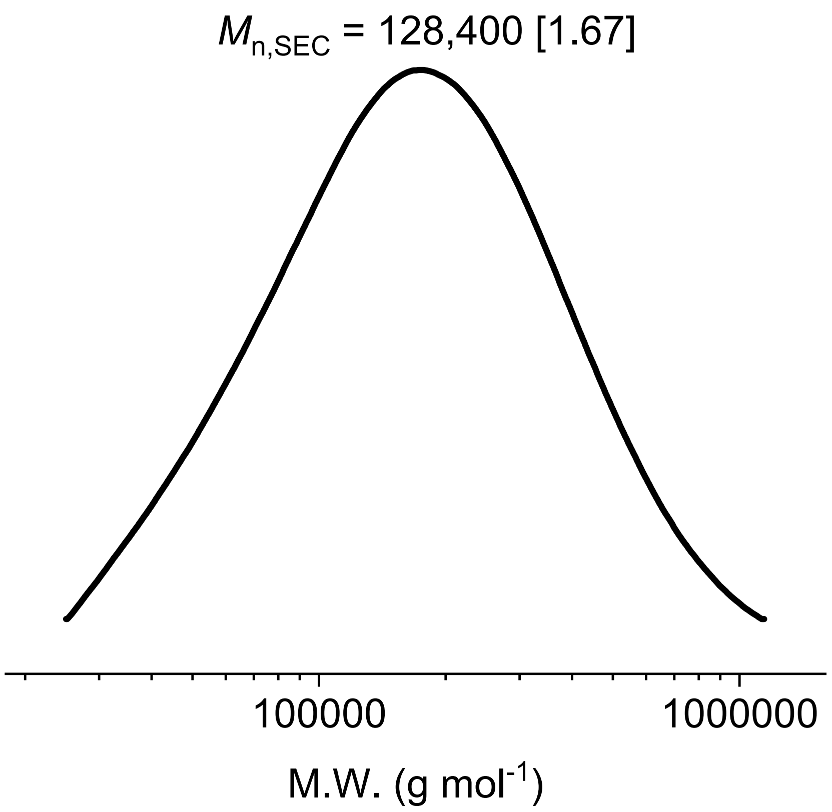

Figure S33. SEC chromatograms of PVL formed in DSC pan determined in cholorofrm eluent. Reaction conditions: 160 °C, $[Sn(Oct)_2]_0$:$[BnOH]_0$:$[dVL]_0$ = 1:1:1000, in neat monomer ($[dVL]_0$ = 9.98 M).

3.2 Isothermal kinetics for dVL, LLA and TMC ROP and PA/CHO ROCOP

Table S4. ROP of dVL using $Sn(Oct)_2$/BnOH catalyst at 100, 105, 110, 115 and 120 °C

Entry	Temp. (°C)	k_{obs} [a] ($x10^{-4}$ s^{-1})	k_{obs} [b] (x 10^{-4} s^{-1})	TOF[c] (h^{-1})
1	100	4.66636	4.7 ± 0.1	100±10
		4.75489		
		4.55581		
2	105	4.66636	5.3 ± 0.9	150±20
		4.66636		
		6.57562		
3	110	7.62195	6.5 ± 0.8	160±10
		6.08643		
		5.91017		
4	115	9.53289	8.7 ± 0.6	190±10
		8.01282		
		8.66551		
5	120	13.33333	11.9 ± 1.0	250±10
		11.17318		
		11.23848		

Reactions conducted in DSC pans with $Sn(Oct)_2$: BnOH: dVL loadings of 1: 1: 100 in neat dVL, $[dVL]_0$ = 9.98 M.[a] Determined from exponential fit of conversion *vs* time profiles from 0 – 90% conversion.[b] k_{obs}, error = standard deviation from 3 repeats [d] TOF = moles of dVL consumed/time * moles of catalyst. Determined from 0 – 30% conversion.

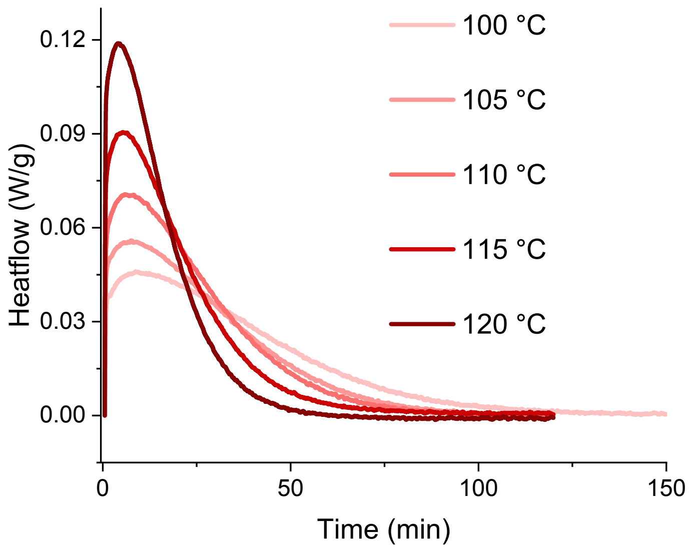

Figure S34. Plots of heatflow *vs* time for the ROP of dVL. Reactions were performed at 100, 105, 110, 115 and 120 °C with loadings of $[Sn(Oct)_2]_0$: $[BnOH]_0$: $[dVL]_0$ = 1: 1: 100 in neat dVL. $[dVL]_0$ = 9.98 M.

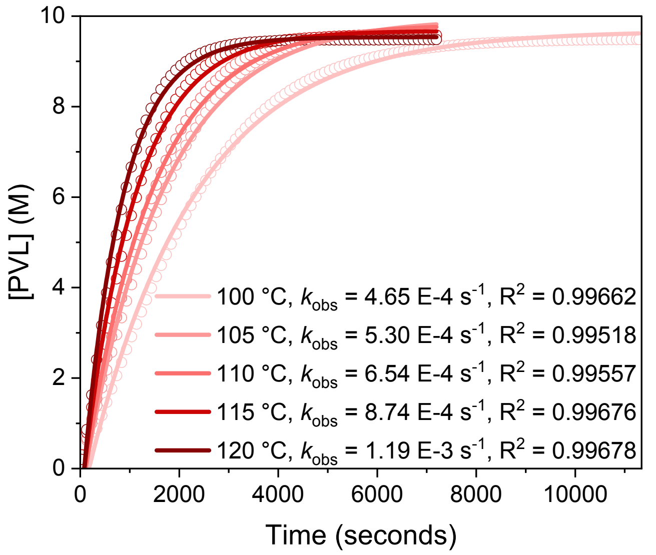

Figure S35. Plots of [PVL] *vs* time. Reactions were performed at 100, 105, 110, 115 and 120 °C with loadings of $[Sn(Oct)_2]_0$: $[BnOH]_0$: $[dVL]_0$ = 1: 1: 100 in neat dVL. $[dVL]_0$ = 9.98 M

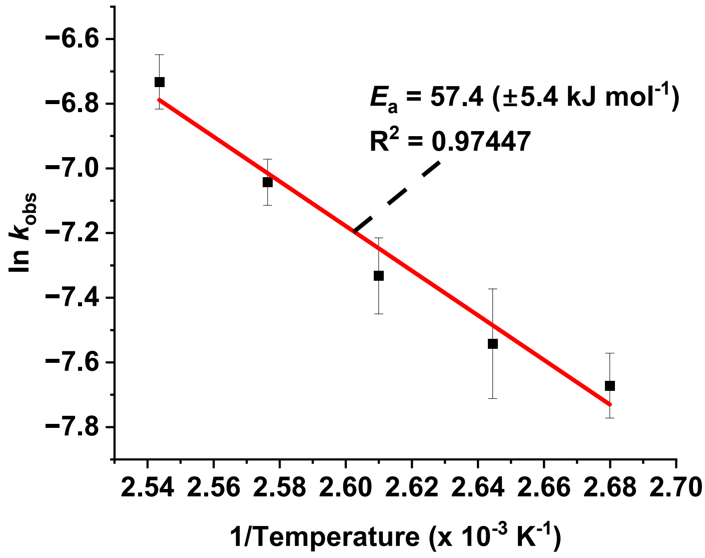

Figure S36. Plot of ln k_{obs} vs 1/Temperature for the ROP of dVL. Reactions were performed at 100, 105, 110, 115 and 120 °C with loadings of $[Sn(Oct)_2]_0$: $[BnOH]_0$: $[dVL]_0$ = 1: 1: 100 in neat dVL. $[dVL]_0$ = 9.98 M. Data taken from table S4.

Table S5. ROP of LLA using $Sn(Oct)_2$/BnOH catalyst at 120, 125, 130, 135 and 140 °C

Entry	Temp. (°C)	k[a] (x 10^{-3} s^{-1})	k_{obs} [b] (x 10^{-3} s^{-1})	TOF[c] (h^{-1})
1	120	1.45<br>1.35<br>1.55	1.5 ± 0.1	260 ± 10
2	125	1.89<br>1.74<br>1.94	1.9 ± 0.1	330 ± 10
3	130	2.42<br>1.94<br>1.94	2.1 ± 0.2	370 ± 20
4	135	3.07<br>3.68<br>2.43	3.1 ± 0.5	470 ± 20
5	140	3.76<br>3.70<br>3.27	3.6 ± 0.2	570 ± 30

Reactions conducted in DSC pans with $Sn(Oct)_2$: BnOH: LLA loadings of 1: 1: 100, $[LLA]_0$ = 1 M in 1,2-dimethoxybenzene.[a] Determined from exponential fit of conversion *vs* time profiles from 0 – 90% conversion.[b] k_{obs} = average of 3 repeats, error = standard deviation from 3 repeats [d] TOF = moles of LLA consumed/time * moles of catalyst. Determined from 0 – 30% conversion.

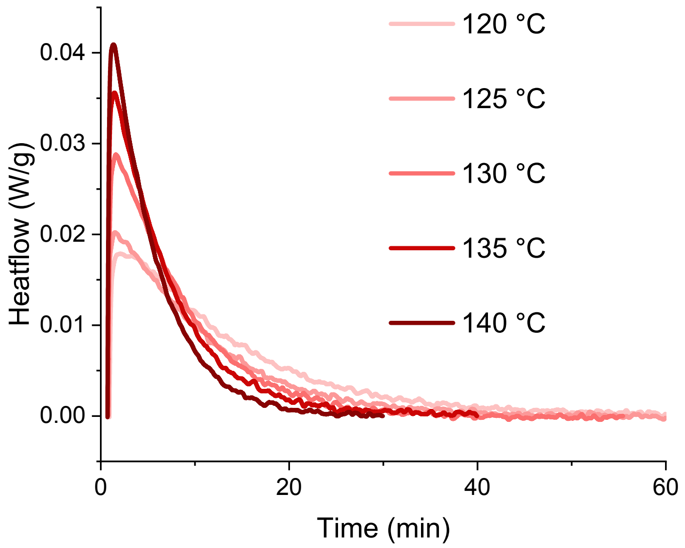

Figure S37. Plots of heatflow *vs* time for the ROP of LLA. Reactions were performed at 120, 125, 130, 135 and 140 °C with loadings of $[Sn(Oct)_2]_0$: $[BnOH]_0$: $[LLA]_0$ = 1: 1: 100. $[LLA]_0$ = 1 M in 1,2-dimethoxybenzene.

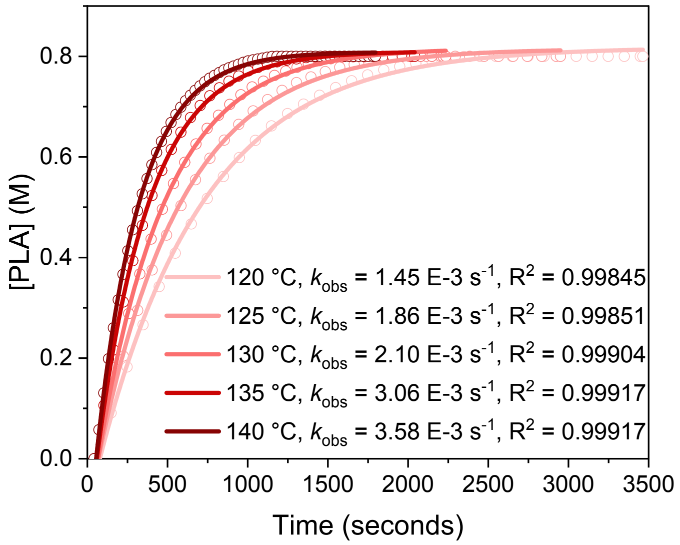

Figure S38. Plots of [PLLA] *vs* time. Reactions were performed at 120, 125, 130, 135 and 140 °C with loadings of $[Sn(Oct)_2]_0$: $[BnOH]_0$: $[LLA]_0$ = 1: 1: 100. $[LLA]_0$ = 1 M in 1,2-dimethoxybenzene.

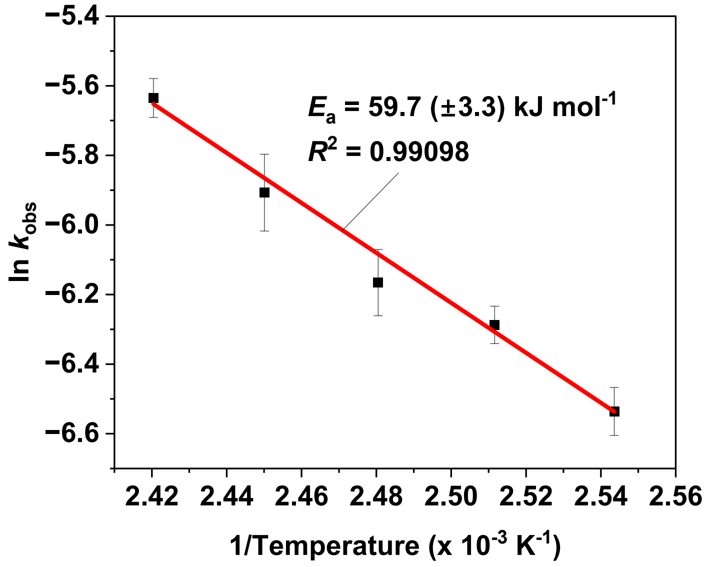

Figure S39. Plot of ln k_{obs} vs 1/temperature for the ROP of LLA. Reactions were performed at 120, 125, 130, 135 and 140 °C with loadings of $[Sn(Oct)_2]_0$: $[BnOH]_0$: $[LLA]_0$ = 1: 1: 100. $[LLA]_0$ = 1 M in 1,2-dimethoxybenzene. Data taken from table S5

Table S6. Solution ROP of TMC using $Sn(Oct)_2$/BnOH catalyst at 120, 130, 135, 140 and 150 °C

Entry	Temp. (°C)	k[a] (x 10^{-3} s^{-1})	k_{obs} [b] (x 10^{-3} s^{-1})	TOF[c] (h^{-1})
1	120	0.577	0.56 ± 0.01	110 ± 10
		0.536		
		0.559		
2	130	1.00	1.0 ± 0.1	180 ± 10
		1.10		
		0.980		
3	135	1.56	1.56 ± 0.1	250 ± 10
		1.56		
		1.57		
4	140	1.80	1.8 ± 0.1	300 ± 10
		1.79		
		1.81		
5	150	3.14	3.2 ± 0.08	540 ± 40
		3.32		
		3.13		

Reactions conducted in DSC pans with $Sn(Oct)_2$: BnOH: TMC loadings of 1: 1: 100, $[TMC]_0$ = 2 M, in 1,2-dimethoxybenzene.[a] Determined from exponential fit of conversion *vs* time profiles from 0 – 90% conversion.[b] k_{obs} = average of 3 repeats, error = standard deviation from 3 repeats [d] TOF = moles of TMC consumed/time * moles of catalyst. Determined from 0 – 30% conversion.

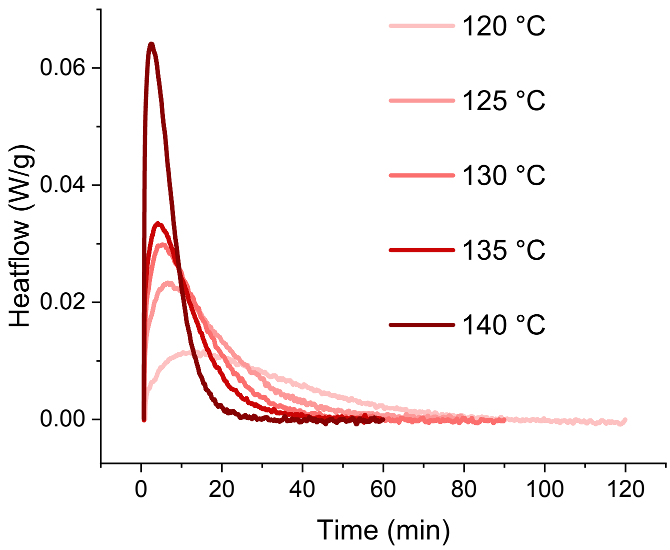

Figure S40. Plot of heatflow vs time for the ROP of TMC. Reactions were performed at 120, 130, 135, 140 and 150 °C with loadings of $[Sn(Oct)_2]_0$: $[BnOH]_0$: $[TMC]_0$ = 1: 1: 100 $[TMC]_0$ = 2 M, in 1,2-dimethoxybenzene.

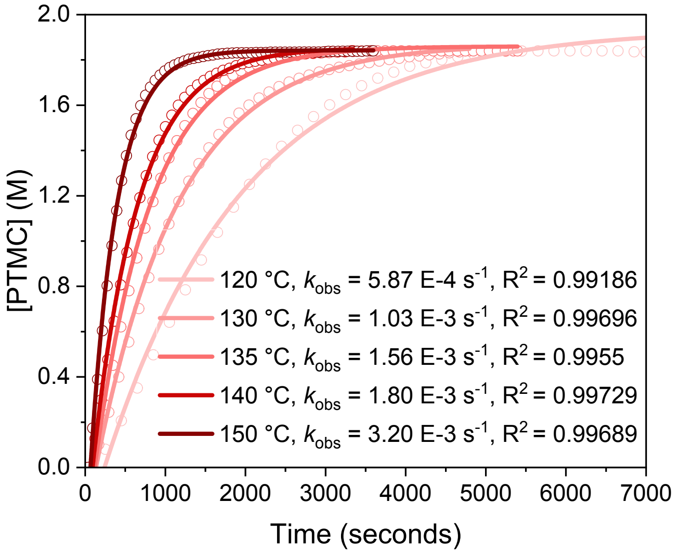

Figure S41. Plots of [PTMC] vs time. Reactions were performed at 120, 130, 135, 140 and 150 °C with loadings of $[Sn(Oct)_2]_0$: $[BnOH]_0$: $[TMC]_0$ = 1: 1: 100. $[TMC]_0$ = 2 M, 1,2-dimethoxybenzene.

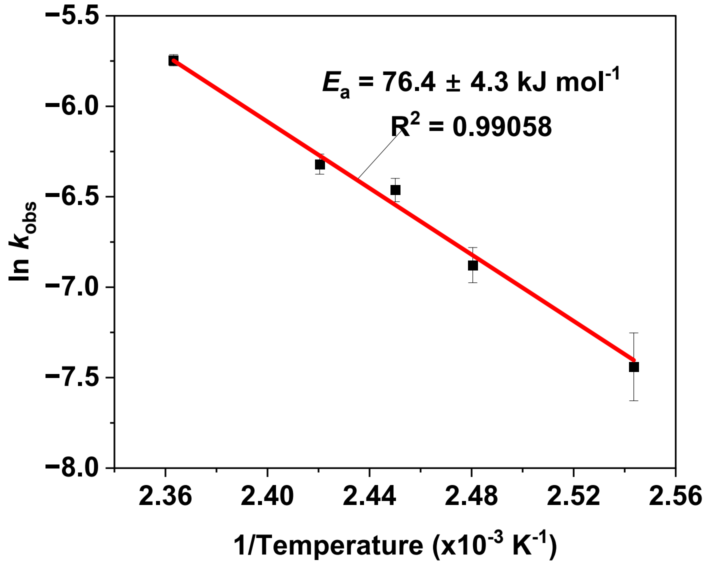

Figure S42. Plot of ln k_{obs} vs 1/temperature for the ROP of TMC. Reactions were performed at 120, 130, 135, 140 and 150 °C with loadings of $[Sn(Oct)_2]_0$: $[BnOH]_0$: $[TMC]_0$ = 1: 1: 100. $[TMC]_0$ = 2 M in 1,2-dimethoxybenzene. Data taken from table S6

3.3 ROP of dVL, LLA and TMC under dynamic heating

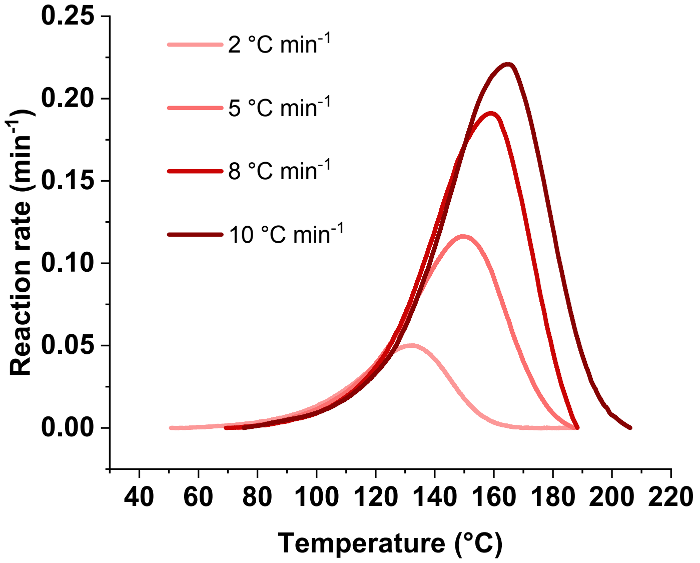

Figure S43. Plots of reaction rate *vs* temperature for the ROP of dVL with heating rates of 2, 5, 8 and 10 °C min^{-1}. $[Sn(Oct)_2]_0$: $[BnOH]_0$: $[dVL]_0$ = 1: 1: 100 in neat dVL. $[dVL]_0$ = 9.98 M.

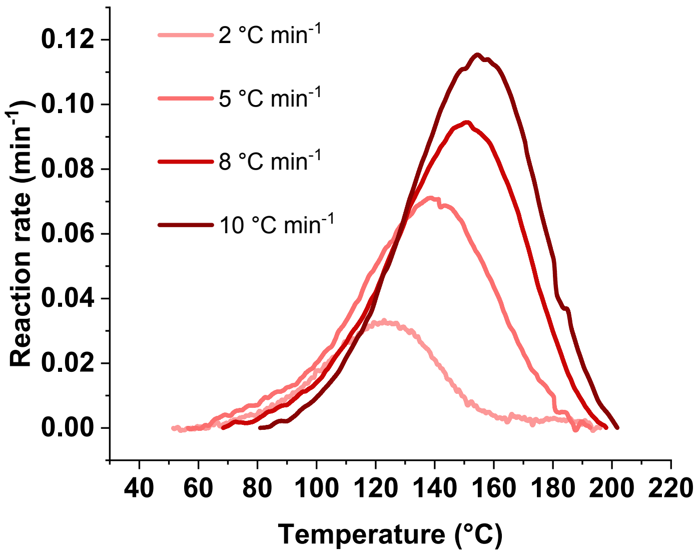

Figure S44. Plots of reaction rate *vs* temperature for the ROP of LLA with heating rates of 2, 5, 8 and 10 °C min^{-1}. $[Sn(Oct)_2]_0$: $[BnOH]_0$: $[LLA]_0$ = 1: 1: 100. $[LLA]_0$ = 1 M in 1,2-dimethoxybenzene

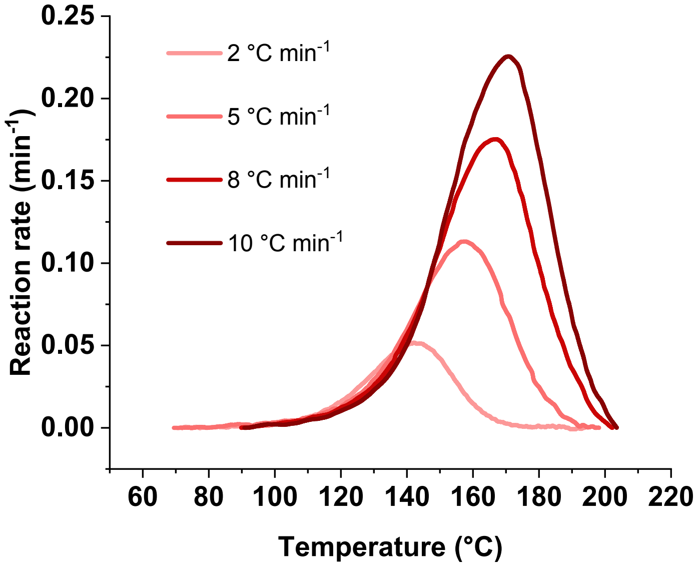

Figure S45. Plots of reaction rate *vs* temperature for the ROP of TMC with heating rates of 2, 5, 8 and 10 °C min^{-1}. $[Sn(Oct)_2]_0$: $[BnOH]_0$: $[TMC]_0$ = 1: 1: 100. $[TMC]_0$ = 2 M in 1,2-dimethoxybenzene.

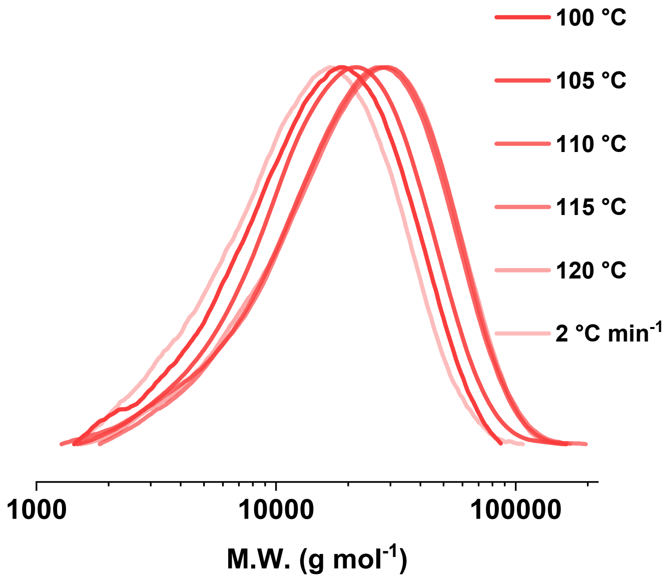

Figure S46. Representative SEC chromatograms of polymer formed in DSC pan at different isotherms or under dynamic heating rates. Polymerizations were conducted at 105, 110, 115 120 °C and at 2 °C min^{-1} 40 -200 °C using $[Sn(Oct)_2]_0$:$[BnOH]_0$:$[dVL]_0$ 1: 1: 100. $[dVl]_0$ = 9.98 M

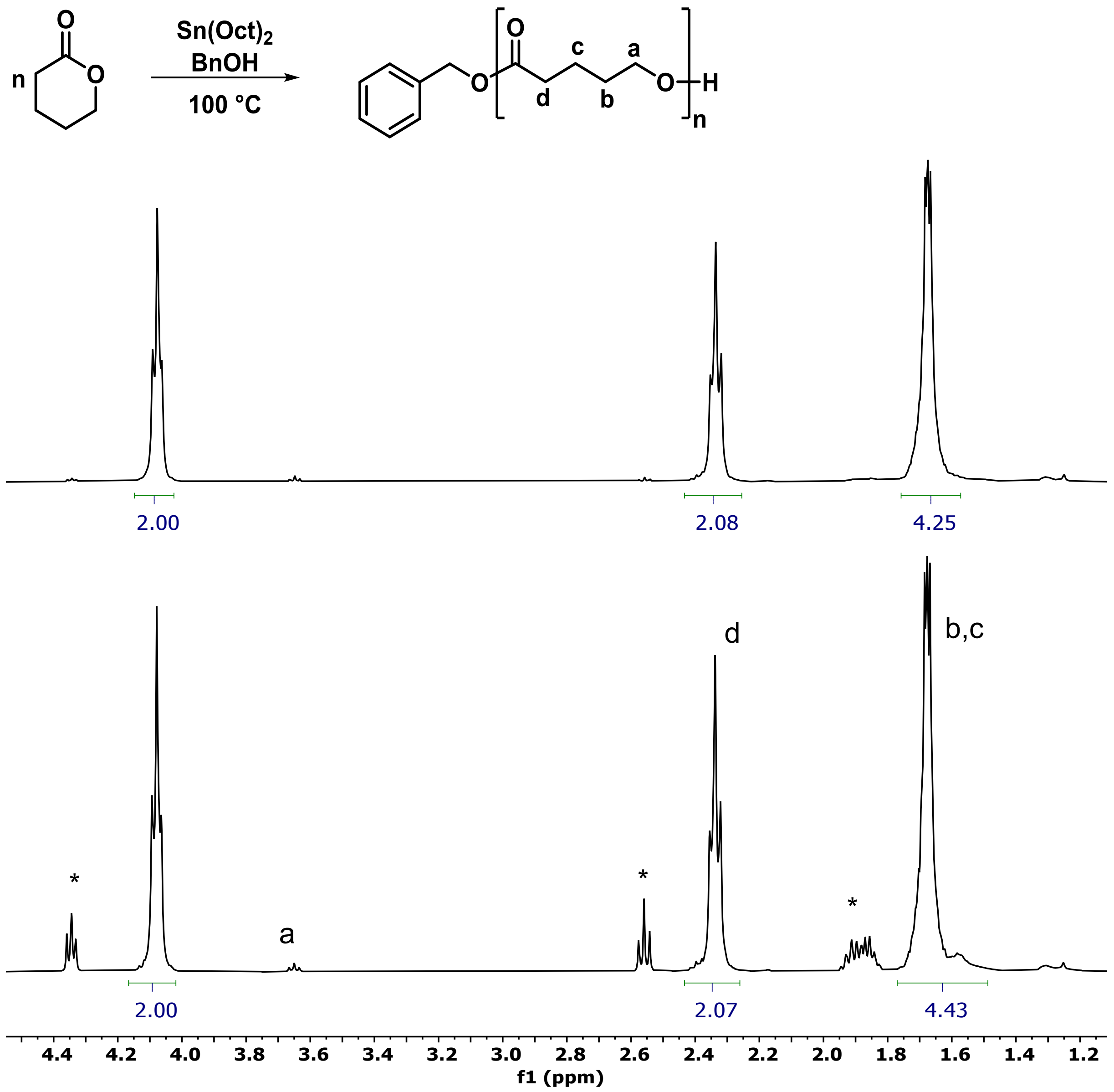

Figure S47. Stacked crude ^{1}H NMR spectra from dVL ROP conducted in DSC pan at 120 °C or at heating rate of 2 °C min^{-1} between 40 – 200 °C. Reactions were conducted at $[Sn(Oct)_2]_0$:$[BnOH]_0$:$[dVL]_0$ = 1:1:100, in neat dVL. $[dVL]_0$ = 9.98 M. * indicates residual monomer

3.5 dVL ROP kinetics by single heating ramp

Table S7. Temperature, fraction conversion and rate determined at 20 evenly spaced sections between 5 and 65% conversion of dVL ROP conducted a heating rate of 5 °C min^{-1} between 40 – 200 °C.

Temp. (°C)[a]	1/Temp (K^{-1})	Fractional conversion, α[a]	Rate (s^{-1})[a]	ln(1-α)	ln *k*[b]
108.56	0.00262	0.04999	3.31E-4	-0.05128	-7.96172
110.85	0.0026	0.05975	3.8E-4	-0.06161	-7.81365
113.14	0.00259	0.07095	4.37667E-4	-0.07359	-7.66036
115.43	0.00257	0.08385	5.02E-4	-0.08758	-7.50922
117.72	0.00256	0.09861	5.71667E-4	-0.10382	-7.3633
120.01	0.00254	0.1153	6.49667E-4	-0.12251	-7.21648
122.29	0.00253	0.13421	7.38667E-4	-0.14411	-7.06589
124.58	0.00251	0.15582	8.34167E-4	-0.16939	-6.91936
126.87	0.0025	0.1801	9.39667E-4	-0.19857	-6.77085
129.16	0.00249	0.20745	0.00105	-0.2325	-6.62504
131.45	0.00247	0.23795	0.00118	-0.27174	-6.47443
133.74	0.00246	0.27194	0.0013	-0.31737	-6.32858
136.03	0.00244	0.30931	0.00143	-0.37006	-6.18064
138.32	0.00243	0.35019	0.00156	-0.43108	-6.03479
140.61	0.00242	0.3946	0.00167	-0.50187	-5.89118
142.9	0.0024	0.44189	0.00178	-0.5832	-5.74982
145.19	0.00239	0.49197	0.00186	-0.67721	-5.6095
147.47	0.00238	0.54365	0.00192	-0.7845	-5.47093
149.76	0.00236	0.59679	0.00194	-0.9083	-5.33755
152.05	0.00235	0.64989	0.00192	-1.04951	-5.20624

Reaction conditions: $[Sn(Oct)_2]_0$: $[BnOH]_0$: $[dVL]_0$ = 1: 1: 100 in neat dVL. $[dVL]_0$ = 9.98 M. [a] Determined from 5 °C min^{-1} ramp in figure S43 [b] $\ln k = \ln\frac{d\alpha}{dt} - \ln(1-\alpha)$

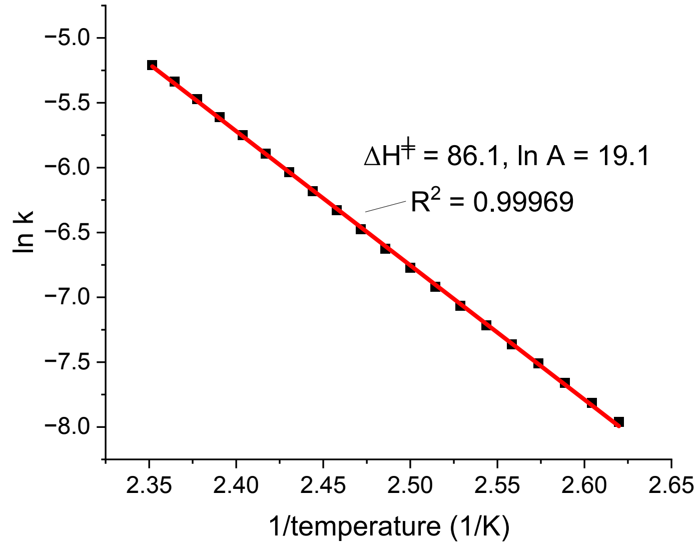

Figure S48. Plot of ln k *vs* 1/temperature for for the ROP of dVL. Reaction conditions: heating rate of 5 °C min^{-1}, 40 – 200 °C, $[Sn(Oct)_2]_0$: $[BnOH]_0$: $[dVL]_0$ = 1: 1: 100 in neat dVL ($[dVL]_0$ = 9.98 M). Data taken from table S7.

Table S8. Temperature, fraction conversion and rate determined at 20 evenly spaced sections between 5 and 65% conversion of dVL ROP conducted a heating rate of 8 °C min^{-1} between 40 – 200 °C.

Temp. (°C)[a]	1/Temp (K^{-1})	Fractional conversion, α[a]	Rate (s^{-1})[a]	ln(1-α)	ln k[b]
117.04	0.00256	0.05003	5.07833E-4	-0.05132	-7.53449
119.28	0.00255	0.05921	5.86833E-4	-0.06104	-7.38013
121.53	0.00253	0.0698	6.76E-4	-0.07236	-7.22689
123.78	0.00252	0.08204	7.75833E-4	-0.0856	-7.07672
126.02	0.00251	0.09606	9.03833E-4	-0.10099	-6.90756
128.27	0.00249	0.11246	0.00103	-0.1193	-6.75572
130.52	0.00248	0.13115	0.00119	-0.14058	-6.59024
132.76	0.00246	0.15258	0.00137	-0.16556	-6.43
135.01	0.00245	0.17711	0.00155	-0.19493	-6.27252
137.25	0.00244	0.20485	0.00175	-0.22922	-6.1211
139.5	0.00242	0.23592	0.00194	-0.26908	-5.97351
141.75	0.00241	0.27038	0.00215	-0.31523	-5.82602
143.99	0.0024	0.30838	0.00236	-0.36872	-5.6783
146.24	0.00238	0.3497	0.00256	-0.43032	-5.53848
148.49	0.00237	0.39467	0.00275	-0.50198	-5.39619
150.73	0.00236	0.44184	0.0029	-0.58311	-5.25878
152.98	0.00235	0.49188	0.00302	-0.67704	-5.12597
155.23	0.00233	0.54341	0.00311	-0.78397	-4.98887
157.47	0.00232	0.59623	0.00317	-0.90691	-4.84625
159.72	0.00231	0.64999	0.00318	-1.04979	-4.70074

Reaction conditions: $[Sn(Oct)_2]_0$: $[BnOH]_0$: $[dVL]_0$ = 1: 1: 100 in neat dVL. $[dVL]_0$ = 9.98 M. [a] Determined from from 8 °C min^{-1} ramp in figure S43 [b] $\ln k = \ln\frac{d\alpha}{dt} - \ln(1-\alpha)$

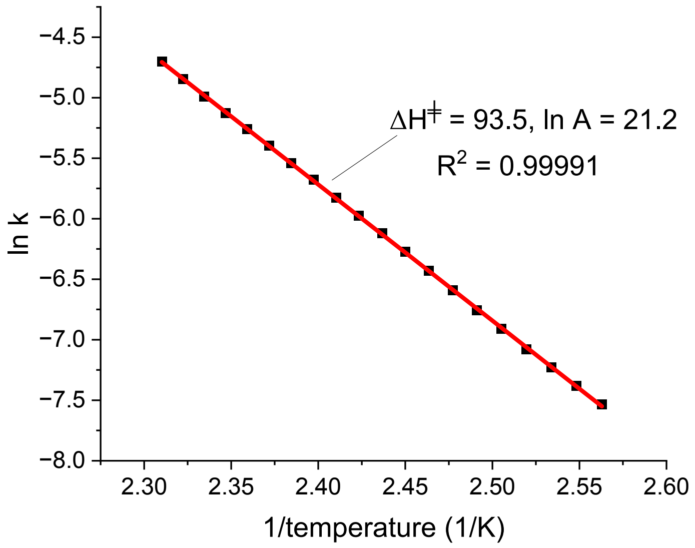

Figure S49. Plot of ln k *vs* 1/temperature for for the ROP of dVL. Reaction conditions: heating rate of 5 °C min^{-1}, 40 – 200 °C, $[Sn(Oct)_2]_0$: $[BnOH]_0$: $[dVL]_0$ = 1: 1: 100 in neat dVL ($[dVL]_0$ = 9.98 M). Data taken from table S8.

Table S9. Temperature, fraction conversion and rate determined at 20 evenly spaced sections between 5 and 65% conversion of dVL ROP conducted a heating rate of 10 °C min^{-1} between 40 – 200 °C.

Temp. (°C)[a]	1/Temp (K^{-1})	Fractional conversion, α[a]	Rate (s^{-1})[a]	ln(1-α)	ln *k*[b]
120.57	0.00254	0.04997	5.72667E-4	-0.05126	-7.4135
122.98	0.00252	0.05888	6.58833E-4	-0.06068	-7.26485
125.38	0.00251	0.06904	7.56833E-4	-0.07154	-7.11534
127.79	0.00249	0.08082	8.86E-4	-0.08427	-6.94503
130.19	0.00248	0.09454	0.00103	-0.09931	-6.77689
132.6	0.00246	0.11074	0.0012	-0.11737	-6.60488
135	0.00245	0.12928	0.00139	-0.13843	-6.43813
137.41	0.00244	0.15091	0.00159	-0.16359	-6.28331
139.81	0.00242	0.17516	0.00181	-0.19257	-6.12375
142.22	0.00241	0.20306	0.00204	-0.22698	-5.9669
144.62	0.00239	0.23405	0.0023	-0.26664	-5.8104
147.02	0.00238	0.26883	0.00254	-0.31311	-5.66361
149.43	0.00237	0.30725	0.00279	-0.36709	-5.5135
151.83	0.00235	0.34888	0.00303	-0.42906	-5.37049
154.24	0.00234	0.39411	0.00325	-0.50106	-5.22816
156.64	0.00233	0.44219	0.00342	-0.58374	-5.0944
159.05	0.00231	0.49272	0.00355	-0.67869	-4.96388
161.45	0.0023	0.54405	0.00363	-0.78537	-4.83337
163.86	0.00229	0.59707	0.00368	-0.90899	-4.69709
166.26	0.00228	0.65002	0.00366	-1.04988	-4.55973

Reactions conditions: $[Sn(Oct)_2]_0$: $[BnOH]_0$: $[dVL]_0$ = 1: 1: 100 in neat dVL. $[dVL]_0$ = 9.98 M. [a] Determined from from 10 °C min^{-1} ramp in figure S43 [b] $\ln k = \ln\frac{d\alpha}{dt} - \ln(1-\alpha)$

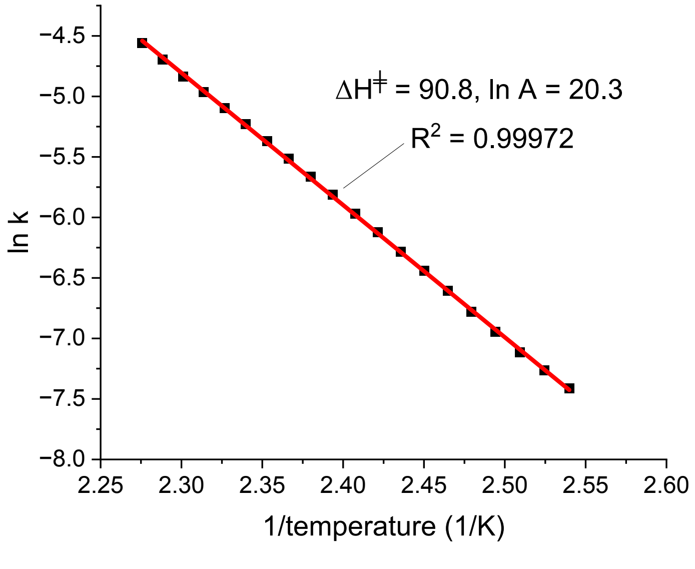

Figure S50. Plot of ln k *vs* 1/temperature for for the ROP of dVL. Reaction conditions: heating rate of 10 °C min^{-1}, 40 – 200 °C, $[Sn(Oct)_2]_0$: $[BnOH]_0$: $[dVL]_0$ = 1: 1: 100 in neat dVL ($[dVL]_0$ = 9.98 M). Data taken from table S9.

3.5 LLA ROP kinetics by single heating ramp

Table S10. Temperature, fraction conversion and rate determined at 20 evenly spaced sections between 5 and 40% conversion of LLA ROP conducted a heating rate of 2 °C min^{-1} between 40 – 200 °C.

Temp. (°C)[a]	1/Temp (K^{-1})	Fractional conversion, α[a]	Rate (s^{-1})[a]	ln(1-α)	ln *k*[b]
89.54	0.00276	0.04997	1.5E-4	-0.05126	-8.7527
91.24	0.00274	0.05816	1.66167E-4	-0.05992	-8.64224
92.95	0.00273	0.06724	1.82E-4	-0.06961	-8.54211
94.65	0.00272	0.07709	2.05333E-4	-0.08022	-8.41011
96.35	0.00271	0.08813	2.28833E-4	-0.09226	-8.29041
98.05	0.00269	0.10019	2.53333E-4	-0.10557	-8.17493
99.76	0.00268	0.11375	2.92333E-4	-0.12076	-8.01697
101.46	0.00267	0.12878	2.93833E-4	-0.13786	-7.99411
103.16	0.00266	0.14478	3.29833E-4	-0.1564	-7.86043
104.87	0.00265	0.16212	3.525E-4	-0.17688	-7.77345
106.57	0.00263	0.18074	3.72667E-4	-0.19935	-7.69539
108.27	0.00262	0.20053	4.04E-4	-0.22381	-7.59003
109.97	0.00261	0.2224	4.44167E-4	-0.25154	-7.46777
111.68	0.0026	0.24509	4.435E-4	-0.28116	-7.43959
113.38	0.00259	0.26827	4.585E-4	-0.31234	-7.37498
115.08	0.00258	0.29274	4.88667E-4	-0.34636	-7.27715
116.78	0.00256	0.31844	5.09167E-4	-0.38337	-7.19917
118.49	0.00255	0.34507	5.23E-4	-0.42323	-7.13263
120.19	0.00254	0.37241	5.38167E-4	-0.46587	-7.06154
121.89	0.00253	0.39987	5.40667E-4	-0.51061	-7.01206

Reaction conditions: $[Sn(Oct)_2]_0$: $[BnOH]_0$: $[LLA]_0$ = 1: 1: 100. $[LLA]_0$ = 1 M in 1,2-dimethoxybenzene. [a] Determined from 2 °C min^{-1} ramp in figure S44 [b] $\ln k = \ln\frac{d\alpha}{dt} - \ln(1-\alpha)$

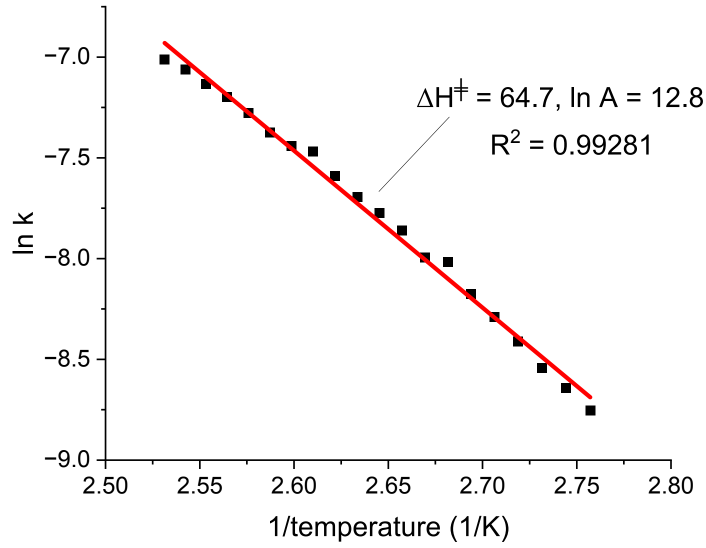

Figure S51. Plot of ln k *vs* 1/temperature for for the ROP of LLA. Reaction conditions: heating rate of 2 °C min^{-1}, 40 – 200 °C, $[Sn(Oct)_2]_0$:$[BnOH]_0$:$[LLA]_0$ = 1:1:100, $[LLA]_0$ = 1 M in 1,2-dimethoxybenzene. Data taken from table S10.

Table S11. Temperature, fraction conversion and rate determined at 20 evenly spaced sections between 5 and 40% conversion of LLA ROP conducted a heating rate of 5 °C min^{-1} between 40 – 200 °C.

Temp. (°C)[a]	1/Temp (K^{-1})	Fractional conversion, α[a]	Rate (s^{-1})[a]	ln(1-α)	ln *k*[b]
95.22	0.00271	0.05	2.62167E-4	-0.05129	-8.19551
97.38	0.0027	0.05725	2.95833E-4	-0.05895	-8.06711
99.53	0.00268	0.06521	3.28333E-4	-0.06743	-7.95382
101.68	0.00267	0.07413	3.665E-4	-0.07702	-7.83431
103.84	0.00265	0.08419	4.05667E-4	-0.08795	-7.72184
105.99	0.00264	0.09503	4.455E-4	-0.09985	-7.61636
108.15	0.00262	0.10752	5.10667E-4	-0.11375	-7.46595
110.3	0.00261	0.12127	5.58333E-4	-0.12928	-7.36114
112.46	0.00259	0.1365	6.19E-4	-0.14676	-7.24057
114.61	0.00258	0.15315	6.68833E-4	-0.16623	-7.1435
116.76	0.00256	0.17152	7.50667E-4	-0.18816	-7.00615
118.92	0.00255	0.19178	8.05167E-4	-0.21292	-6.91148
121.07	0.00254	0.21334	8.59667E-4	-0.23996	-6.81914
123.23	0.00252	0.23642	9.23333E-4	-0.26974	-6.718
125.38	0.00251	0.26071	9.6E-4	-0.30207	-6.64655
127.53	0.0025	0.28619	0.00102	-0.33714	-6.54857
129.69	0.00248	0.31323	0.00106	-0.37576	-6.4702
131.84	0.00247	0.34117	0.0011	-0.41729	-6.39593
134	0.00246	0.37021	0.00114	-0.46237	-6.31195
136.15	0.00244	0.39998	0.00117	-0.51079	-6.24393

Reaction conditions: $[Sn(Oct)_2]_0$:$[BnOH]_0$:$[LLA]_0$ = 1:1:100. $[LLA]_0$ = 1 M in 1,2-dimethoxybenzene. [a] Determined from 5 °C min^{-1} ramp in figure S44 [b] $\ln k = \ln\frac{d\alpha}{dt} - \ln(1-\alpha)$

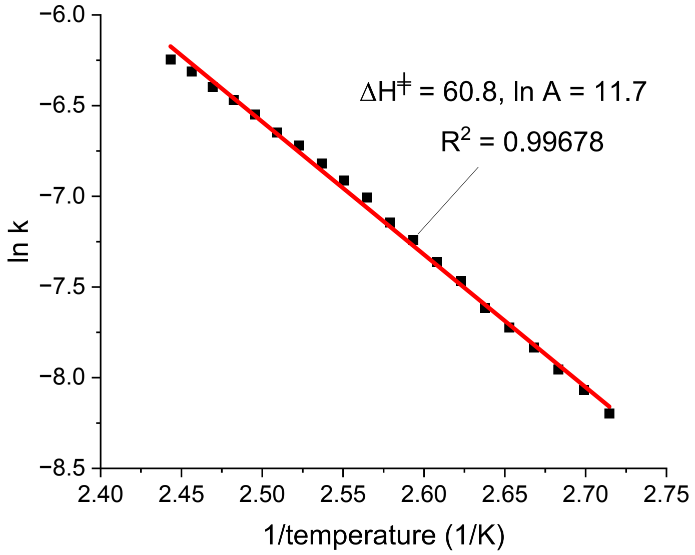

Figure S52. Plot of ln k *vs* 1/temperature for for the ROP of LLA. Reaction conditions: heating rate of 5 °C min^{-1}, 40 – 200 °C, $[Sn(Oct)_2]_0$:$[BnOH]_0$:$[LLA]_0$ = 1:1:100, $[LLA]_0$ = 1 M in 1,2-dimethoxybenzene. Data taken from table S11.

Table S12. Temperature, fraction conversion and rate determined at 20 evenly spaced sections between 5 and 40% conversion of LLA ROP conducted a heating rate of 8 °C min^{-1} between 40 – 200 °C.

Temp. (°C)[a]	1/Temp (K^{-1})	Fractional conversion, α[a]	Rate (s^{-1})[a]	ln(1-α)	ln *k*[b]
110.86	0.0026	0.05001	4.63333E-4	-0.0513	-7.62613
113.09	0.00259	0.0582	5.13167E-4	-0.05996	-7.51471
115.33	0.00257	0.06724	5.63333E-4	-0.06961	-7.41232
117.56	0.00256	0.07718	6.30333E-4	-0.08032	-7.2891
119.79	0.00254	0.08827	6.92167E-4	-0.09241	-7.18332
122.02	0.00253	0.10049	7.73E-4	-0.10591	-7.05906
124.26	0.00252	0.11424	8.61833E-4	-0.12131	-6.93518
126.49	0.0025	0.1293	9.415E-4	-0.13846	-6.82954
128.72	0.00249	0.14572	0.00102	-0.1575	-6.7342
130.95	0.00247	0.16338	0.00112	-0.17839	-6.6198
133.19	0.00246	0.18278	0.00119	-0.20185	-6.53292
135.42	0.00245	0.20333	0.00126	-0.22731	-6.453
137.65	0.00243	0.22491	0.00133	-0.25478	-6.36829
139.88	0.00242	0.24764	0.00139	-0.28454	-6.29336
142.12	0.00241	0.27148	0.00145	-0.31674	-6.21983
144.35	0.0024	0.29608	0.0015	-0.35109	-6.15043
146.58	0.00238	0.32171	0.00155	-0.38818	-6.08446
148.81	0.00237	0.34765	0.00156	-0.42717	-6.03591
151.05	0.00236	0.37401	0.00157	-0.46842	-5.98548
153.28	0.00235	0.40007	0.00154	-0.51094	-5.96532

Reaction conditions: $[Sn(Oct)_2]_0$:$[BnOH]_0$:$[LLA]_0$ = 1:1:100. $[LLA]_0$ = 1 M in 1,2-dimethoxybenzene. [a] Determined from 8 °C min^{-1} ramp in figure S44 [b] $\ln k = \ln \frac{d\alpha}{dt} - \ln(1-\alpha)$

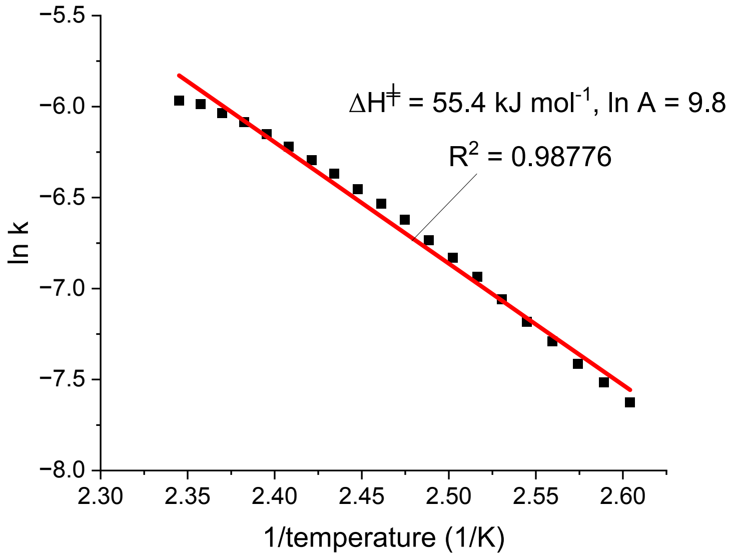

Figure S53. Plot of ln k *vs* 1/temperature for for the ROP of LLA. Reaction conditions: heating rate of 8 °C min^{-1}, 40 – 200 °C, $[Sn(Oct)_2]_0$:$[BnOH]_0$:$[LLA]_0$ = 1:1:100, $[LLA]_0$ = 1 M in 1,2-dimethoxybenzene. Data taken from table S12.

Table S13. Temperature, fraction conversion and rate determined at 20 evenly spaced sections between 5 and 35% conversion of LLA ROP conducted a heating rate of 10°C min^{-1} between 40 – 200 °C.

Temp. (°C)[a]	1/Temp (K^{-1})	Fractional conversion, α[a]	Rate (s^{-1})[a]	ln(1-α)	ln *k*[b]
119.65	0.00255	0.04999	6.49333E-4	-0.05128	0.04102
121.54	0.00253	0.05793	7.41333E-4	-0.05968	0.0472
123.42	0.00252	0.06676	8.12667E-4	-0.06909	0.05224
125.31	0.00251	0.07629	8.80167E-4	-0.07936	0.05715
127.2	0.0025	0.0867	9.62167E-4	-0.09069	0.0632
129.08	0.00249	0.09807	0.00105	-0.10322	0.06998
130.97	0.00247	0.11052	0.00115	-0.11712	0.07774
132.86	0.00246	0.12416	0.00124	-0.13257	0.0848
134.74	0.00245	0.13852	0.00133	-0.1491	0.09243
136.63	0.00244	0.15411	0.0014	-0.16737	0.09956
138.52	0.00243	0.17043	0.00148	-0.18685	0.10729
140.4	0.00242	0.18422	0.00155	-0.20361	0.11566
142.29	0.00241	0.20583	0.00165	-0.23046	0.12444
144.18	0.0024	0.2248	0.00171	-0.25463	0.13257
146.06	0.00239	0.24452	0.00177	-0.2804	0.14084
147.95	0.00237	0.26506	0.00183	-0.30797	0.14913
149.84	0.00236	0.28587	0.00185	-0.33669	0.15535
151.72	0.00235	0.30687	0.00188	-0.36654	0.16251
153.61	0.00234	0.32833	0.00192	-0.39799	0.1711
155.5	0.00233	0.35002	0.00191	-0.43081	0.17659

Reaction conditions: $[Sn(Oct)_2]_0$:$[BnOH]_0$:$[LLA]_0$ = 1:1:100. $[LLA]_0$ = 1 M in 1,2-dimethoxybenzene. [a] Determined from10 °C min^{-1} ramp in figure S44 [b] $\ln k = \ln\frac{d\alpha}{dt} - \ln(1-\alpha)$

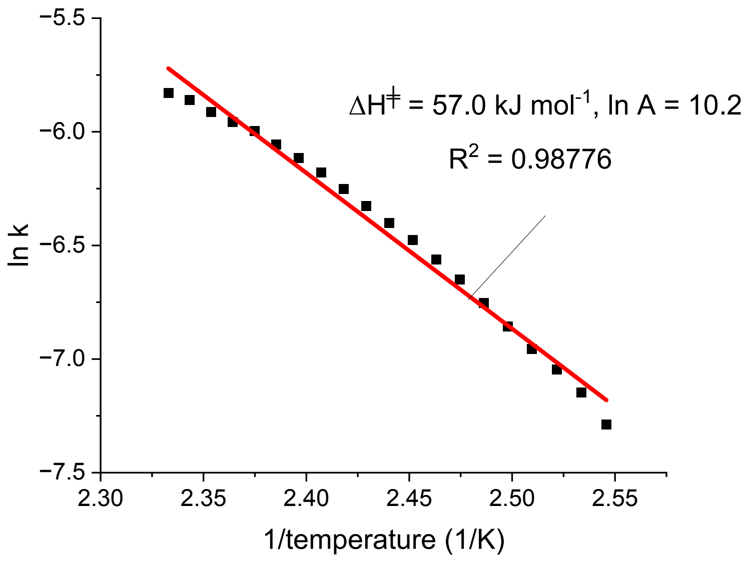

Figure S54. Plot of ln k *vs* 1/temperature for for the ROP of LLA. Reaction conditions: heating rate of 10 °C min^{-1}, 40 – 200 °C, $[Sn(Oct)_2]_0$:$[BnOH]_0$:$[LLA]_0$ = 1:1:100, $[LLA]_0$ = 1 M in 1,2-dimethoxybenzene. Data taken from table S13.

3.6 TMC ROP kinetics by single heating ramp

Table S14. Temperature, fraction conversion and rate determined at 20 evenly spaced sections between 5 and 55% conversion of TMC ROP conducted a heating rate of 2°C min^{-1} between 40 – 200 °C.

Temp. (°C)[a]	1/Temp (K^{-1})	Fractional conversion, α[a]	Rate (s^{-1})[a]	ln(1-α)	ln *k*[b]
114.69	0.00258	0.05	1.705E-4	-0.05129	-8.62556
116.29	0.00257	0.05876	1.88833E-4	-0.06056	-8.51375
117.89	0.00256	0.0686	2.21167E-4	-0.07107	-8.34562
119.49	0.00255	0.0802	2.57333E-4	-0.0836	-8.1811
121.09	0.00254	0.09326	2.9E-4	-0.0979	-8.04742
122.69	0.00253	0.10811	3.26833E-4	-0.11441	-7.91059
124.3	0.00252	0.12502	3.78E-4	-0.13355	-7.74693
125.9	0.00251	0.14435	4.24833E-4	-0.15589	-7.60763
127.5	0.0025	0.16593	4.73167E-4	-0.18144	-7.47434
129.1	0.00249	0.19	5.275E-4	-0.21072	-7.33629
130.7	0.00248	0.21658	5.76E-4	-0.24409	-7.21477
132.31	0.00247	0.2459	6.375E-4	-0.28223	-7.07589
133.91	0.00246	0.27771	6.87333E-4	-0.32533	-6.95716
135.51	0.00245	0.31201	7.395E-4	-0.37398	-6.83521
137.11	0.00244	0.3486	7.84167E-4	-0.42863	-6.72198
138.71	0.00243	0.38693	8.12E-4	-0.48928	-6.62622
140.32	0.00242	0.42704	8.42667E-4	-0.55694	-6.52187
141.92	0.00241	0.46797	8.61E-4	-0.63106	-6.42635
143.52	0.0024	0.50905	8.515E-4	-0.71141	-6.35696
145.12	0.00239	0.54987	8.41333E-4	-0.79822	-6.28222

Reaction condutions: $[Sn(Oct)_2]_0$:$[BnOH]_0$:$[TMC]_0$ = 1:1:100. $[TMC]_0$ = 2 M in 1,2-dimethoxybenzene. [a] Determined from 2 °C min^{-1} ramp in figure S45 [b] $\ln k = \ln \frac{d\alpha}{dt} - \ln(1-\alpha)$

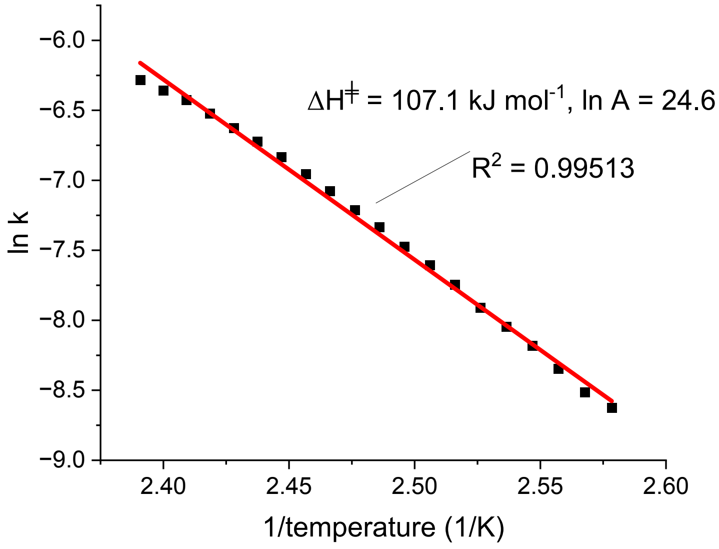

Figure S55. Plot of ln k *vs* 1/temperature for for the ROP of TMC. Reaction conditions: heating rate of 2 °C min^{-1}, 40 – 200 °C, $[Sn(Oct)_2]_0$:$[BnOH]_0$:$[TMC]_0$ = 1:1:100, $[TMC]_0$ = 2 M in 1,2-dimethoxybenzene. Data taken from table S14.

Table S15. Temperature, fraction conversion and rate determined at 20 evenly spaced sections between 5 and 55% conversion of TMC ROP conducted a heating rate of 5°C min^{-1} between 40 – 200 °C.

Temp. (°C)[a]	1/Temp (K^{-1})	Fraction conversion[a]	Rate (sec^{-1})[a]	ln(1-fraction conversion)	ln *k*[b]
124.78	0.00251	0.04998	3.21833E-4	-0.05127	-7.98994
126.66	0.0025	0.05771	3.65667E-4	-0.05944	-7.85358
128.55	0.00249	0.06671	4.275E-4	-0.06904	-7.68878
130.43	0.00248	0.07729	5.025E-4	-0.08044	-7.51582
132.31	0.00247	0.08938	5.77E-4	-0.09363	-7.36445
134.19	0.00245	0.10345	6.64167E-4	-0.1092	-7.20798
136.07	0.00244	0.11942	7.635E-4	-0.12717	-7.05063
137.95	0.00243	0.13788	8.75167E-4	-0.14836	-6.89267
139.83	0.00242	0.15891	9.84E-4	-0.17306	-6.75104
141.71	0.00241	0.18242	0.00111	-0.20141	-6.60491
143.59	0.0024	0.20888	0.00123	-0.23431	-6.46438
145.47	0.00239	0.23814	0.00137	-0.27199	-6.32028
147.35	0.00238	0.27053	0.0015	-0.31544	-6.18491
149.23	0.00237	0.30568	0.0016	-0.36482	-6.07011
151.11	0.00236	0.34291	0.00171	-0.41993	-5.95276
152.99	0.00235	0.38256	0.00179	-0.48217	-5.8429
154.87	0.00234	0.42338	0.00184	-0.55057	-5.74925
156.75	0.00233	0.46555	0.00188	-0.62652	-5.64757
158.64	0.00232	0.5083	0.00188	-0.70989	-5.56927
160.52	0.00231	0.55007	0.00183	-0.79866	-5.50523

Reaction condutions: $[Sn(Oct)_2]_0$:$[BnOH]_0$:$[TMC]_0$ = 1:1:100. $[TMC]_0$ = 2 M in 1,2-dimethoxybenzene. [a] Determined from 5 °C min^{-1} ramp in figure S45 [b] $\ln k = \ln\frac{d\alpha}{dt} - \ln(1-\alpha)$

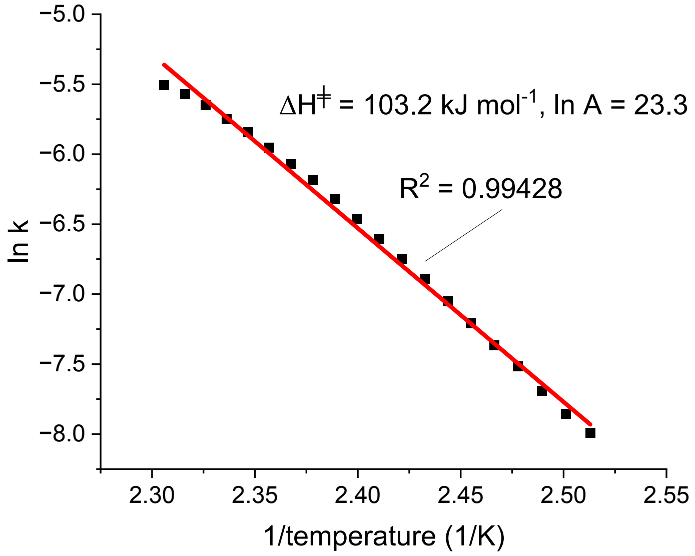

Figure S56. Plot of ln k *vs* 1/temperature for for the ROP of TMC. Reaction conditions: heating rate of 5 °C min^{-1}, 40 – 200 °C, $[Sn(Oct)_2]_0$:$[BnOH]_0$:$[TMC]_0$ = 1:1:100, $[TMC]_0$ = 2 M in 1,2-dimethoxybenzene. Data taken from table S15.

Table S16. Temperature, fraction conversion and rate determined at 20 evenly spaced sections between 5 and 55% conversion of TMC ROP conducted a heating rate of 8°C min^{-1} between 40 – 200 °C.

Temp. (°C)[a]	1/Temp (K^{-1})	Fractional conversion, α[a]	Rate (s^{-1})[a]	ln(1-α)	ln *k*[b]
133.06	0.00246	0.05001	5.445E-4	-0.0513	-7.46466
134.94	0.00245	0.05829	6.31667E-4	-0.06006	-7.30759
136.82	0.00244	0.0678	7.45833E-4	-0.07021	-7.1304
138.7	0.00243	0.07905	8.56667E-4	-0.08235	-6.97972
140.59	0.00242	0.09208	9.765E-4	-0.0966	-6.83534
142.47	0.00241	0.10672	0.00111	-0.11286	-6.69331
144.35	0.0024	0.12341	0.00126	-0.13172	-6.54554
146.23	0.00238	0.14222	0.00143	-0.15341	-6.39572
148.11	0.00237	0.16393	0.00165	-0.17904	-6.22946
149.99	0.00236	0.18844	0.00182	-0.2088	-6.10132
151.87	0.00235	0.21533	0.00202	-0.24249	-5.96112
153.75	0.00234	0.24512	0.0022	-0.2812	-5.84031
155.63	0.00233	0.2776	0.00239	-0.32518	-5.7119
157.51	0.00232	0.31227	0.00253	-0.37436	-5.60491
159.39	0.00231	0.34892	0.00266	-0.42912	-5.49894
161.27	0.0023	0.38738	0.00279	-0.49001	-5.3933
163.15	0.00229	0.42724	0.00286	-0.55729	-5.29875
165.03	0.00228	0.46792	0.0029	-0.63096	-5.21077
166.91	0.00227	0.50904	0.00292	-0.71139	-5.12464
168.8	0.00226	0.55005	0.00288	-0.79862	-5.05045

Reaction condutions: $[Sn(Oct)_2]_0$:$[BnOH]_0$:$[TMC]_0$ = 1:1:100. $[TMC]_0$ = 2 M in 1,2-dimethoxybenzene. [a] Determined from 8 °C min^{-1} ramp in figure S45 [b] $\ln k = \ln \frac{d\alpha}{dt} - \ln(1-\alpha)$

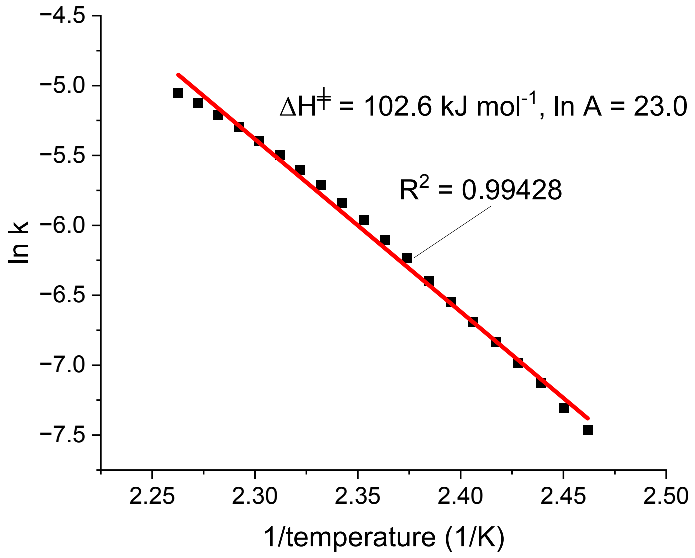

Figure S57. Plot of ln k *vs* 1/temperature for for the ROP of TMC. Reaction conditions: heating rate of 8 °C min^{-1}, 40 – 200 °C, $[Sn(Oct)_2]_0$:$[BnOH]_0$:$[TMC]_0$ = 1:1:100, $[TMC]_0$ = 2 M in 1,2-dimethoxybenzene. Data taken from table S16.

Table S17. Temperature, fraction conversion and rate determined at 20 evenly spaced sections between 5 and 55% conversion of TMC ROP conducted a heating rate of 10°C min^{-1} between 40 – 200 °C.

Temp. (°C)[a]	1/Temp (K^{-1})	Fractional conversion, α[a]	Rate (s^{-1})[a]	ln(1-α)	ln *k*[b]
137.68	0.00243	0.05003	6.96833E-4	-0.05132	-7.21828
139.49	0.00242	0.05815	8.08667E-4	-0.05991	-7.05993
141.31	0.00241	0.06764	9.375E-4	-0.07004	-6.90255
143.12	0.0024	0.07872	0.00109	-0.08199	-6.73747
144.93	0.00239	0.09141	0.00126	-0.09586	-6.5778
146.75	0.00238	0.10647	0.0015	-0.11258	-6.38716
148.56	0.00237	0.12388	0.00172	-0.13225	-6.23421
150.38	0.00236	0.14378	0.00196	-0.15523	-6.0786
152.19	0.00235	0.16633	0.0022	-0.18192	-5.93761
154	0.00234	0.19148	0.00242	-0.21255	-5.81224
155.82	0.00233	0.21904	0.00266	-0.24723	-5.6824
157.63	0.00232	0.24918	0.00291	-0.28659	-5.55458
159.44	0.00231	0.28173	0.00307	-0.33091	-5.45676
161.26	0.0023	0.31617	0.00324	-0.38005	-5.35203
163.07	0.00229	0.35195	0.00339	-0.43379	-5.25353
164.88	0.00228	0.38972	0.00353	-0.49384	-5.15305
166.7	0.00227	0.42878	0.00363	-0.55998	-5.05776
168.51	0.00226	0.46847	0.00371	-0.632	-4.96455
170.32	0.00225	0.50922	0.00376	-0.71176	-4.87243
172.14	0.00225	0.5501	0.00373	-0.79873	-4.79288

Reaction condutions: $[Sn(Oct)_2]_0$:$[BnOH]_0$:$[TMC]_0$ = 1:1:100. $[TMC]_0$ = 2 M in 1,2-dimethoxybenzene. [a] Determined from 10 °C min^{-1} ramp in figure S45 [b] $\ln \boldsymbol{k} = \ln \frac{d\alpha}{dt} - \ln(1-\alpha)$

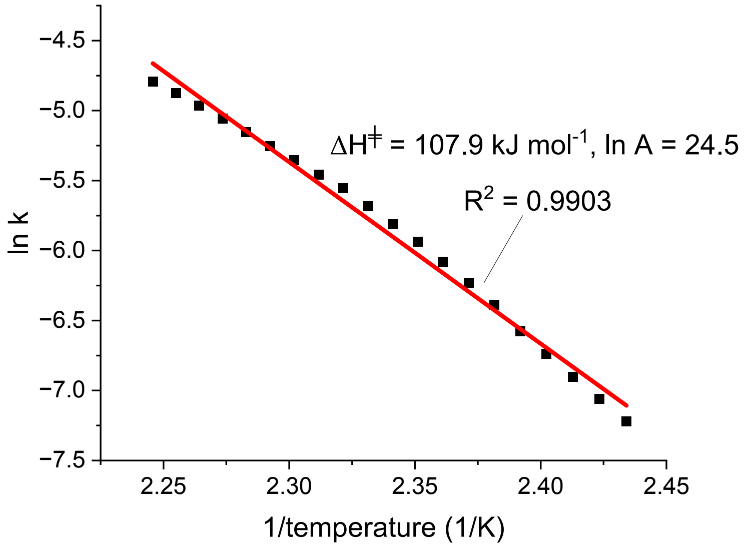

Figure S58. Plot ln k *vs* 1/temperature for for the ROP of TMC. Reaction conditions: heating rate of 10°C min^{-1}, 40 – 200 °C, $[Sn(Oct)_2]_0$:$[BnOH]_0$:$[TMC]_0$ = 1:1:100, $[TMC]_0$ = 2 M in 1,2-dimethoxybenzene. Data taken from table S17.

3.7 LLA and TMC kinetics by multi-heating ramps

Table S18. Heating rate, peak temperature taken from figure S44 for the ROP of LLA conducted under dynamic heating between 40 – 200 °C.

Heating rate, β (°C min^{-1})	Peak temperature (°C)	1/Tempeature (x 10^{-3} K^{-1})	Log β
2	123	2.53	0.3
5	139	2.43	0.7
8	151	2.36	0.9
10	155	2.34	1

Reaction conditions:$[Sn(Oct)_2]_0$:$[BnOH]_0$:$[LLA]_0$ = 1:1:100. $[LLA]_0$ = 1 M in 1,2-dimethoxybenzene

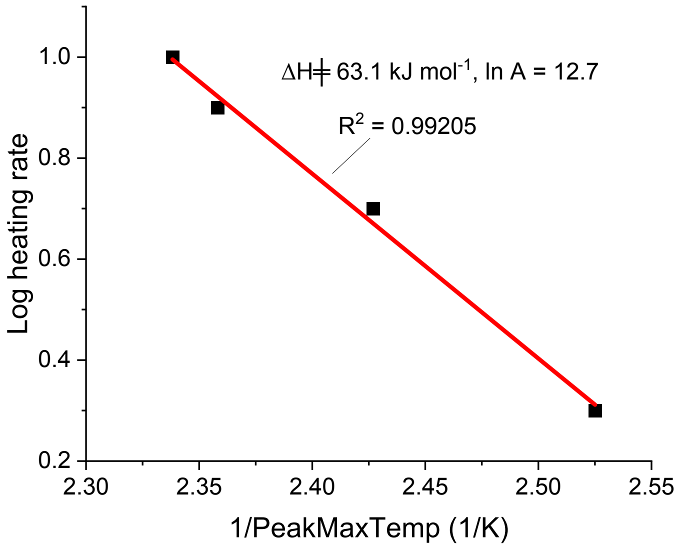

Figure S59. Log heating rate vs 1/temperature for the ROP LLA. Reaction conditions: heating rates of 2, 5, 8 and 10 °C min^{-1}, 40 – 200 °C, $[Sn(Oct)_2]_0$:$[BnOH]_0$:$[LLA]_0$ = 1:1:100, $[LLA]_0$ = 1 M in 1,2-dimethoxybenzene. Data taken from table S18.

Table S19. Heating rate, peak temperature taken from figure S45 for the ROP of TMC conducted under dynamic heating between 40 – 200 °C. X

Heating rate, β (°C min^{-1})	Peak temperature (°C)	1/Tempeature (x 10^{-3} K^{-1})	Log β
2	142	2.41	0.3
5	158	2.32	0.7
8	167	2.27	0.9
10	171	2.25	1

$[Sn(Oct)_2]_0$:$[BnOH]_0$:$[TMC]_0$ = 1:1:100. $[TMC]_0$ = 2 M in 1,2-dimethoxybenzene.

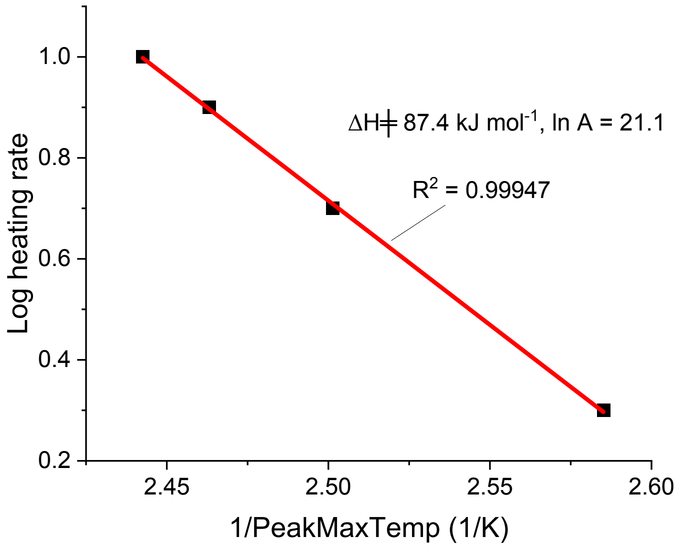

Figure S60. Plot showing log heating rate vs 1/temperature for the ROP TMC. Reaction conditions: heating rates of 2, 5, 8 and 10 °C min^{-1}, 40 – 200 °C, $[Sn(Oct)_2]_0$:$[BnOH]_0$:$[TMC]_0$ = 1:1:100, $[TMC]_0$ = 2 M in 1,2-dimethoxybenzene. Data taken from table S19.

3.8 Comparison of methods for determination of activation paramaters for the kinetics of dVL, TMC and LLA ROP

Table S20. Comparison of methods for determination of Δ*H*‡ and Ln A for the ROP of dVL using a $Sn(Oct)_2$/BnOH catalyst

DSC method	Model	Figure	Δ*H*‡	Ln A
Isothermal	Arrenhius isothermal	S36	57.4 (±5.4)	10.8 ± 1.7
2 °C min^{-1} ramp	Borchardt-Daniels	S2	85.5	19.2
5 °C min^{-1} ramp	Borchardt-Daniels	S48	86.1	19.1
8 °C min^{-1} ramp	Borchardt-Daniels	S49	93.5	21.2
10 °C min^{-1} ramp	Borchardt-Daniels	S50	90.8	20.3
2, 5, 8, 10 °C min^{-1} ramp	Flynn-Wall-Ozawa	S6	66.8	13.5

Reaction conditions: $[Sn(Oct)_2]_0$: $[BnOH]_0$: $[dVL]_0$ = 1: 1: 100 in neat dVL. $[dVL]_0$ = 9.98 M. Borchardt-Daniels, taken between 5 – 65% conversion, with 20 intervals. Reaction order set to 1. Flynn-Wall-Ozawa: calculated from peak temperature

Table S21. Comparison of methods for determination of Δ$H^‡$ and Ln A for the ROP of LLA using a $Sn(Oct)_2$/BnOH catalyst/initiator

DSC method	Model	Figure	Δ$H^‡$	Ln A
Isothermal	Arrenhius isothermal	S39	59.7 ± 3.3	11.7 ± 1.0
2 °C min^{-1} ramp	Borchardt-Daniels	S51	64.7	12.8
5 °C min^{-1} ramp	Borchardt-Daniels	S52	60.8	11.6
8 °C min^{-1} ramp	Borchardt-Daniels	S53	55.5	9.8
10 °C min^{-1} ramp[a]	Borchardt-Daniels	S54	53.7	9.3
2, 5, 8, 10 °C min^{-1} ramp	Flynn-Wall-Ozawa	S59	63.1	15.9

Recation conditions: $[Sn(Oct)_2]_0$: $[BnOH]_0$: $[LLA]_0$ = 1: 1: 100. $[LLA]_0$ = 1 M in 1,2-dimethoxybenzene Borchardt-Daniels, taken between onset of reaction and peak rate (5-40% conversion), with 20 interval. Reaction order set to 1. Flynn-Wall-Ozawa: calculated from peak temperature
[a] calculated from 5 – 35% conversion

Table S22. Comparison of methods for determination of Δ$H^‡$ and Ln A for the ROP of TMC using a $Sn(Oct)_2$/BnOH catalyst/initiator

DSC method	Model	Figure	Δ$H^‡$	Ln A
Isothermal	Arrenhius isothermal	S42	76.4 ± 4.3	16.0 ± 1.2
2 °C min^{-1} ramp	Borchardt-Daniels	S55	107.1	24.6
5 °C min^{-1} ramp	Borchardt-Daniels	S56	103.2	23.3
8 °C min^{-1} ramp	Borchardt-Daniels	S57	102.6	23.0
10 °C min^{-1} ramp	Borchardt-Daniels	S58	107.9	24.5
2, 5, 8, 10 °C min^{-1} ramp	Flynn-Wall-Ozawa	S60	87.5	16.3

Reaction condutions: $[Sn(Oct)_2]_0$:$[BnOH]_0$:$[TMC]_0$ = 1:1:100. $[TMC]_0$ = 2 M in 1,2-dimethoxybenzene. Borchardt-Daniels, taken between onset of reaction and peak rate (5-55% conversion), with 20 intervals. Reaction order set to 1. Flynn-Wall-Ozawa: calculated from peak temperature

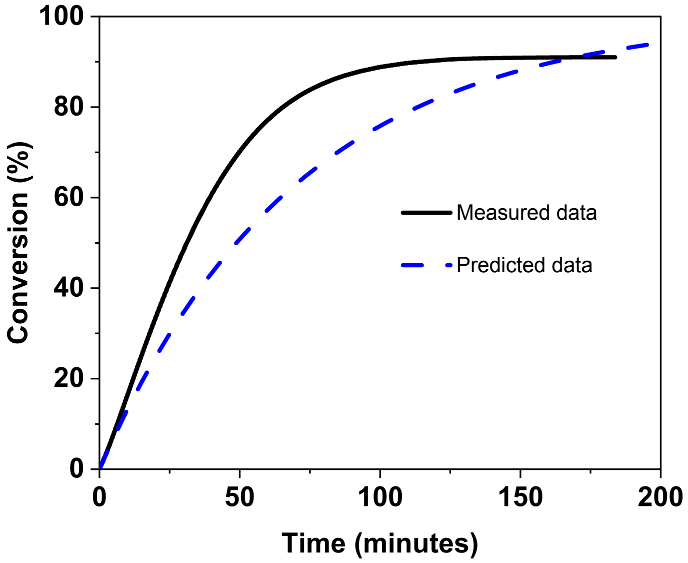

Figure S61. **Conversion *vs* time for the ROP of dVL ([Sn(Oct)$_2$]$_0$: [BnOH]$_0$: [dVL]$_0$ 1: 1: 100, 120 °C, in neat dVL, [dVL]$_0$ = 9.98 M). Dashed black line shows measured data, blue dashed line shows predicted data from acvtivation parameters determined for a 2 °C min^{-1} ramp ($\Delta H^{‡}$ = 85.5, ln A = 19.2)**

3.9 Determination of equilibrium monomer conversions

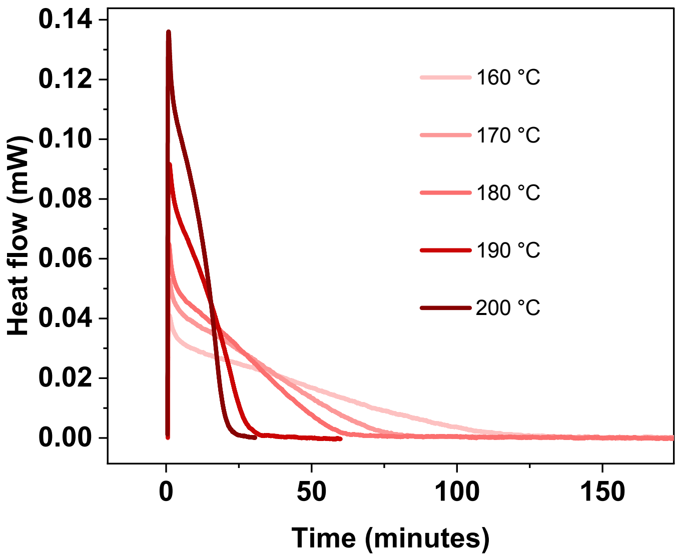

Figure S62. Plots showing heat flow vs time for the polymerization of MDO. Reaction conditions: 160 – 200 °C, $[Sn(Oct)]_2$: [benzyl alcohol]$_0$: [MDO]$_0$ loadings of 1:1:100, in neat MDO, [MDO]$_0$ = 8.61 M.

Table S23. Equilibrium monomer concentrations for the ROP of MDO at 160, 170, 180, 190 and 200 °C

Entry	Temp. (°C)	[Monomer]$_{eq}$[a] (M)	[Monomer]$_{eq}$[b] (M)
1	160	1.145991	1.17 ±0.03
		1.210566	
		1.164072	
2	170	1.337133	1.35 ±0.02
		1.332828	
		1.387071	
3	180	1.43787	1.5 ±0.04
		1.464561	
		1.529997	
4	190	1.539468	1.56 ±0.03
		1.599738	
5	200	1.887312	1.87 ±0.08
		1.775382	
		1.961358	

Reactions conducted in DSC pans with $[Sn(Oct)_2]$: [BnOH]$_0$: [MDO]$_0$ = 1: 1: 100. [MDO]$_0$ = 8.61 M. [a] Determined by ^{1}H NMR spectroscopy. [b]average of at least three repeats. Error taken as standard deviation of the mean.

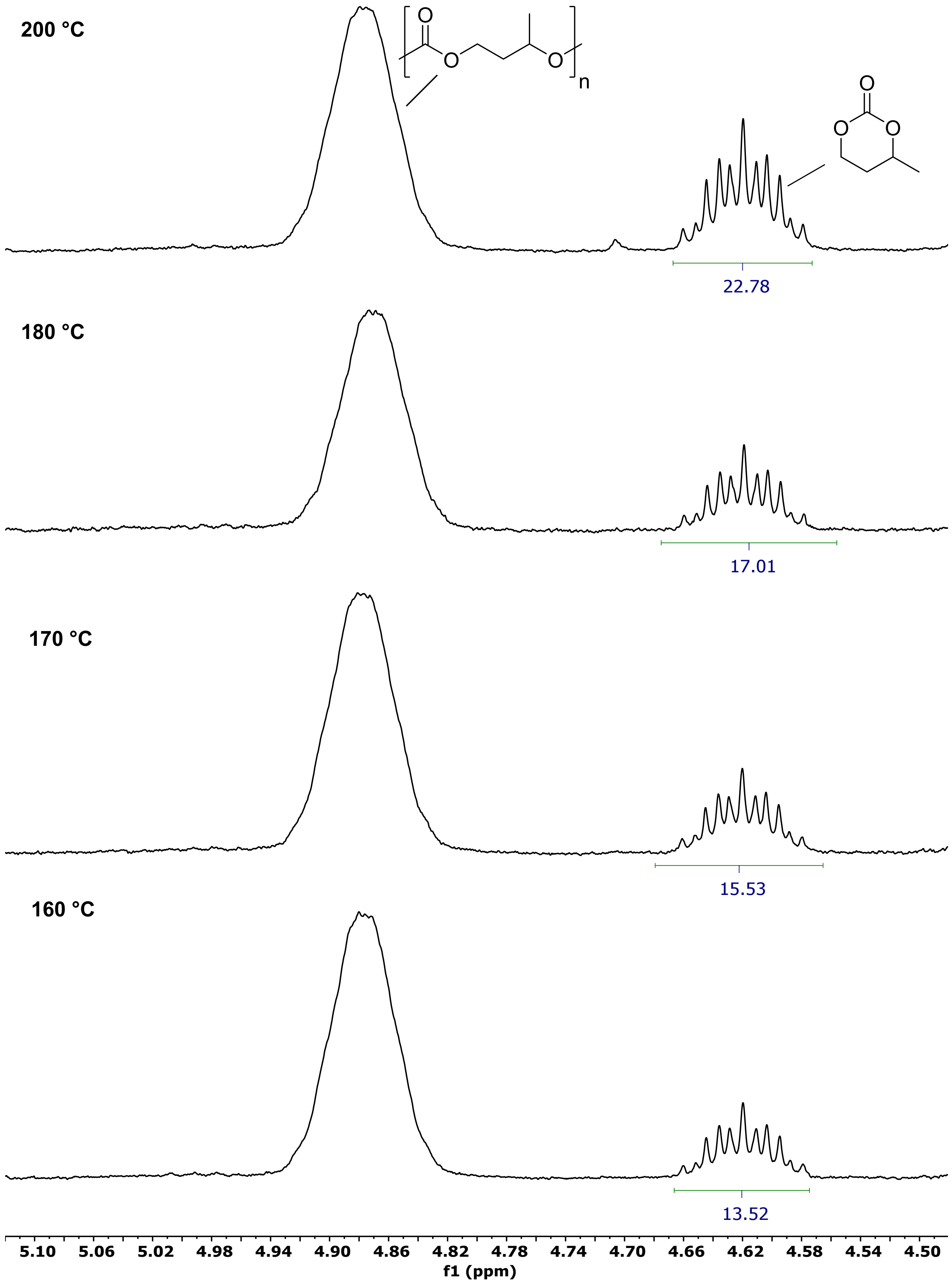

Figure S63. Selected ^{1}H NMR spectra ($CDCl_3$) showing MDO integrals used to determine equilibrium monomer concentration. Reaction conditions: 160 – 200 °C, $[Sn(Oct)]_2$: [benzyl alcohol]$_0$: [MDO]$_0$ = 1:1:100 in neat MDO, [MDO]$_0$ = 8.61 M

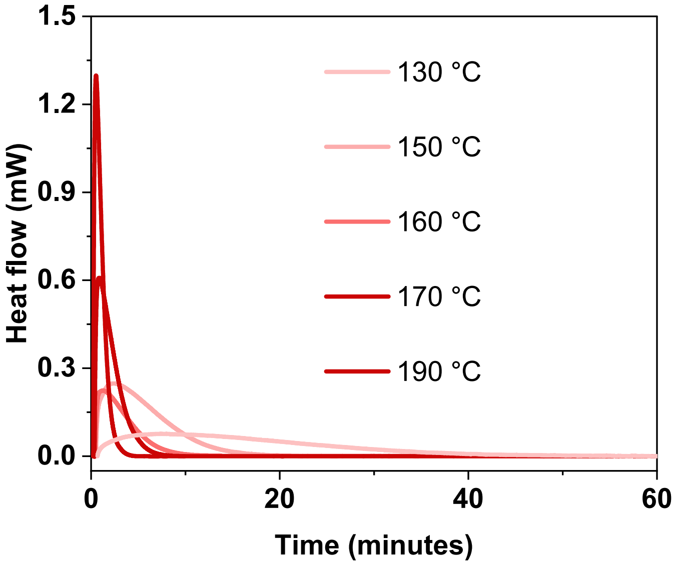

Figure S64. Plots of heat flow vs time for the polymerization of DDO. Reaction conditions: 130 – 190 °C, [Sn(Oct)]$_2$: [benzyl alcohol]$_0$: [DDO]$_0$ loadings of 1:1:500, [DDO]$_0$ = 7.68 M.

Table S24. Equilibrium monomer concentrations for the ROP of DDO at 130, 150, 160, 170 and 190 °C

Entry	Temp. (°C)	[Monomer]$_{eq}$[a] (M)	[Monomer]$_{eq}$[b] (M)
1	130	0.301824	0.31 ±0.01
		0.324096	
2	150	0.457728	0.43 ±0.02
		0.415488	
		0.410112	
3	160	0.507648	0.52 ±0.01
		0.52224	
		0.523008	
4	170	0.615936	0.59 ±0.02
		0.596736	
		0.570624	
5	190	0.807168	0.80 ±0.01
		0.797952	
		0.805632	

Reactions conducted in DSC pans with [Sn(Oct)$_2$]: [BnOH]$_0$: [DDO]$_0$ = 1: 1: 100. [DDO]$_0$ = 7.68 M. [a] Determined by ^{1}H NMR spectroscopy. [b]average of at least three repeats. Error taken as standard deviation of the mean.

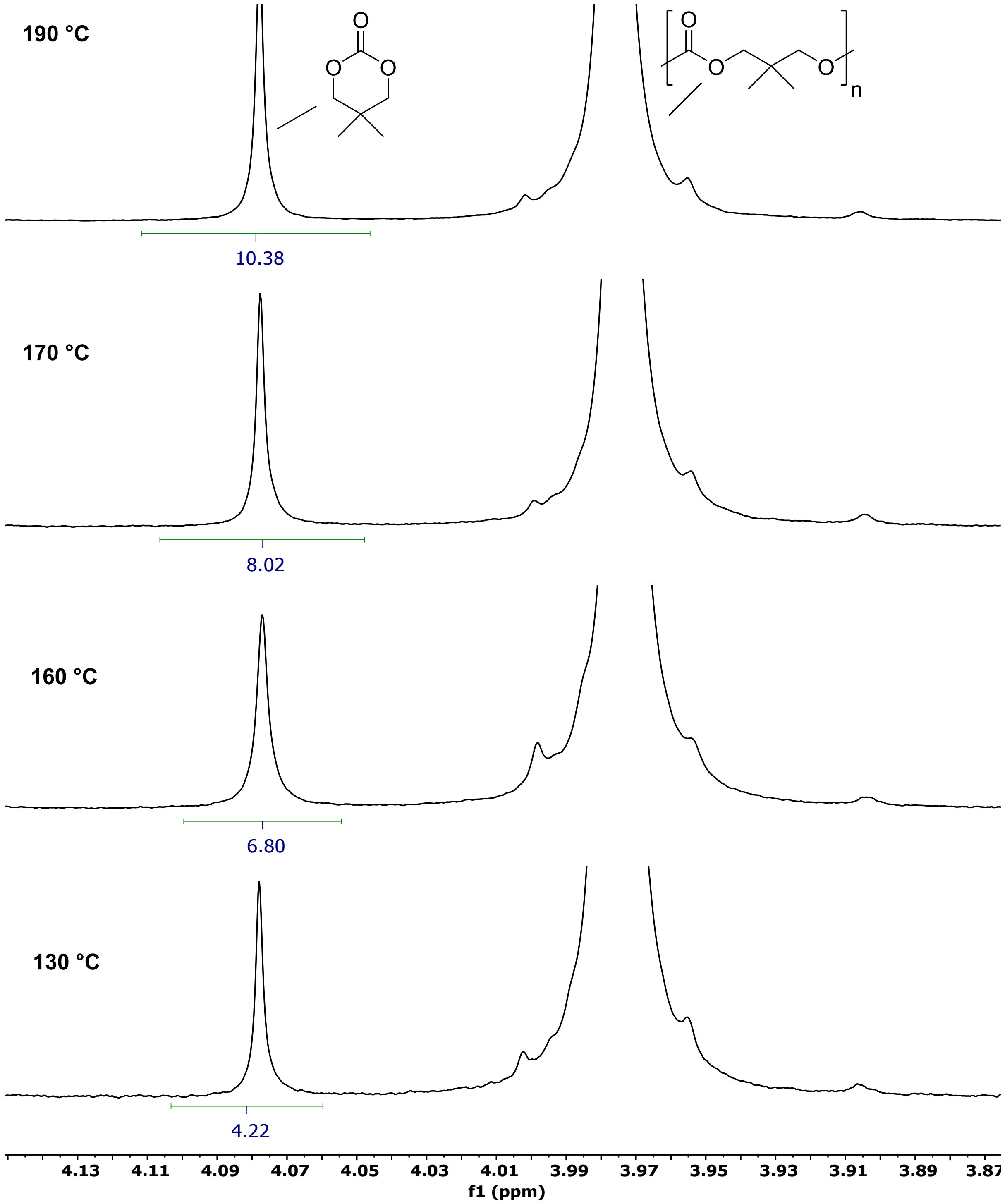

Figure S65. Selected ^{1}H NMR spectra ($CDCl_3$) showing DDO integrals used to determine equilibrium monomer concentration. Reaction conditions: 130 – 190 °C, $[Sn(Oct)]_2$: [benzyl alcohol]$_0$: $[DDO]_0$ = 1:1:100 in neat MDO, $[DDO]_0$ = 7.68 M.

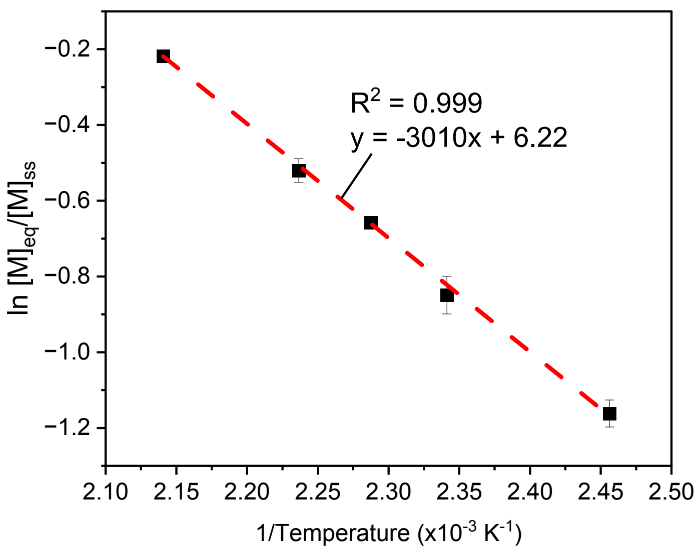

Figure S66. Plot of ln $[M]_{eq}/[M]_{ss}$ *vs* 1/temperature for the ROP of DDO. Reactions conditions: 130 – 190 °C, $[Sn(Oct)_2]_0$: $[BnOH]_0$: $[DDO]_0$ = 1: 1: 100, $[DDO]_0$ = 7.68 M. $[M]_{ss}$ = 1.0 M.

3.10 Examples for new users

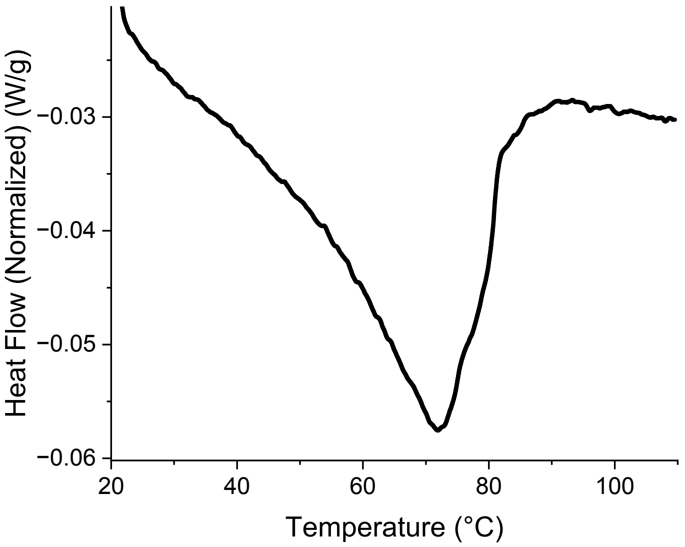

Figure S67. Heatflow *vs* temperature showing the exotherm of dissolving PA in CHO and toluene. The mixture was initially heterogeneous with PA suspended in a solution of $[CHO]_0$ = 5 M in toluene. The initial molar ratio of PA: CHO = 1: 5

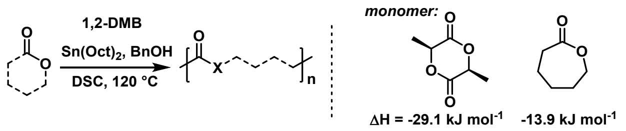

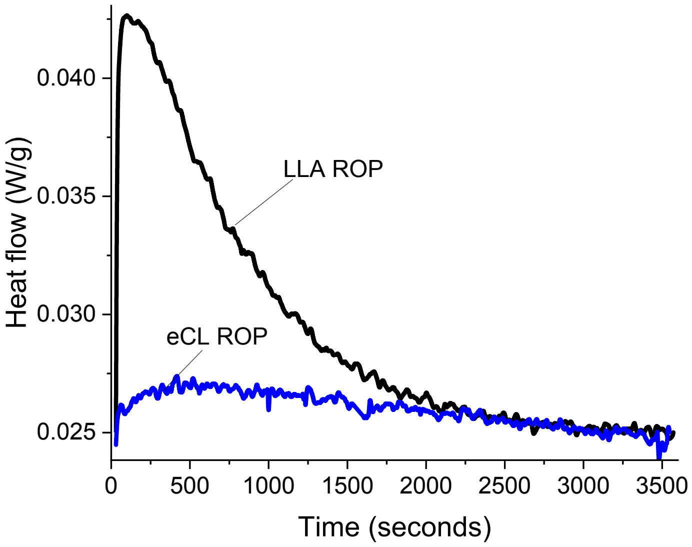

Figure S68. Heatflow *vs* time for the ROP of LLA (black), and *epsilon*-caprolactone (blue). Reactions conditions: 120 °C, $[Sn(Oct)_2]_0$:$[BnOH]_0$:$[monomer]_0$ = 1: 1: 100, $[monomer]_0$ = 1 M in 1,2-dimethoxybenzene. eCL ROP reached 100% conversion in the shown timeframe but the exotherm is not detected owing to the lower enthalpy of the eCL polymerization.

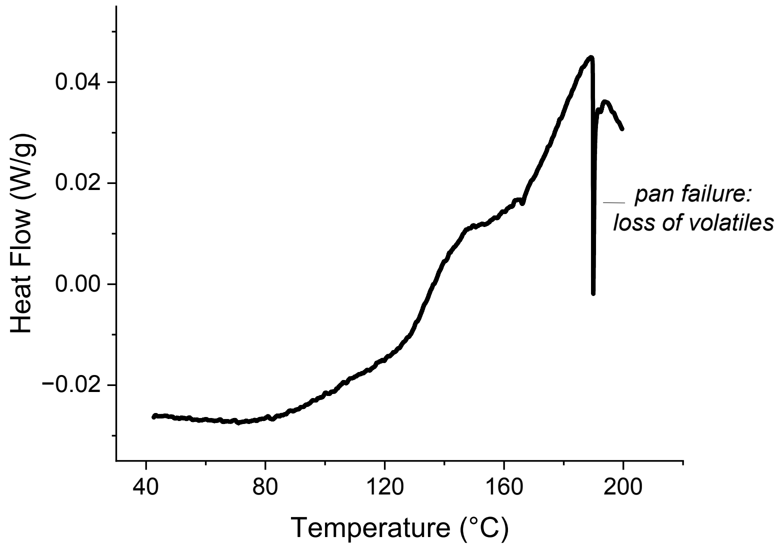

Figure S69. Heatflow *vs* temperature for the ROP of CHO in neat CHO. Reaction conditions: heating rate of 2 °C min^{-1}, [KO^{t}Bu]$_0$: [CHO]$_0$ = 1: 50, [CHO]$_0$ = 10.2 M. Escape of volatiles causes spike in thermogram around 200 °C.

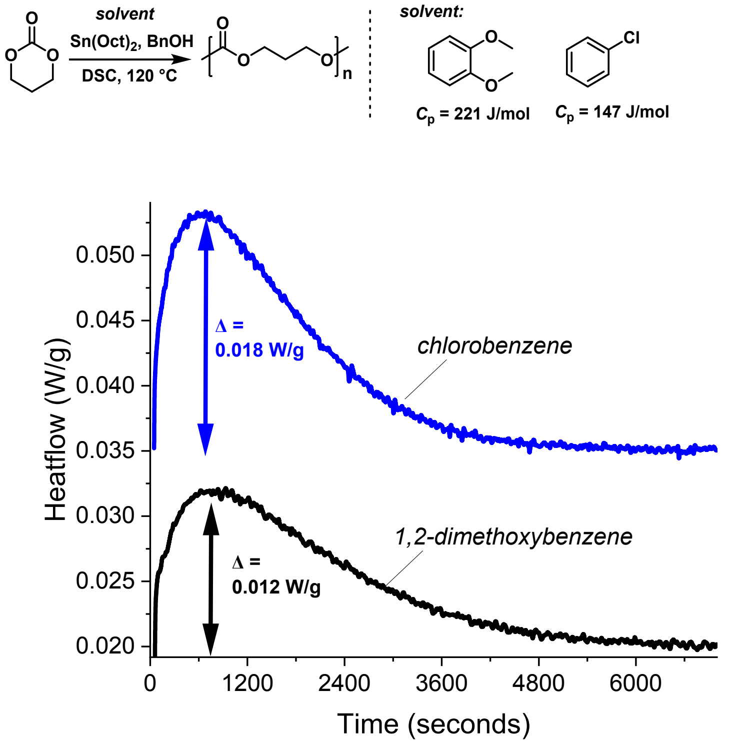

Figure S70. Heatflow *vs* time for the ROP of TMC in 1,2-dimethoxybenzene (black) and chlorobenzene (blue). Reactions conditions: 120 °C, [Sn(Oct)$_2$]$_0$:[BnOH]$_0$:[eCl]$_0$ = 1: 1: 100, [monomer]$_0$ = 2 M in 1,2-dimethoxybenzene.

4. References

(1) Olsson, J. V.; Hult, D.; Cai, Y.; García-Gallego, S.; Malkoch, M. Reactive imidazole intermediates: simplified synthetic approach to functional aliphatic cyclic carbonates. *Polym. Chem.* **2014**, *5* (23), 6651-6655. DOI: https://doi.org/10.1039/C4PY00911H.

(2) Kowalski, A.; Duda, A.; Penczek, S. Polymerization of l,l-Lactide Initiated by Aluminum Isopropoxide Trimer or Tetramer. *Macromolecules* **1998**, *31* (7), 2114-2122. DOI: 10.1021/ma971737k.
